# Efficacy and safety of SQ house dust mite sublingual immunotherapy-tablet (12 SQ-HDM) in children with allergic rhinitis/rhinoconjunctivitis with or without asthma (MT-12): a randomised, double-blind, placebo-controlled, phase III trial

**DOI:** 10.1016/j.lanepe.2024.101136

**Published:** 2024-11-26

**Authors:** Antje Schuster, Davide Caimmi, Hendrik Nolte, Silviya Novakova, Jan Mikler, Majken Hougaard Foss-Skiftesvik, Anne Sofie Østerdal, Andrzej Emeryk, Remi Gagnon, Oliver Pfaar

**Affiliations:** a Center for Paediatric and Adolescent Medicine, University Medical Center, Düsseldorf, Germany; b Allergy Unit, CHU de Montpellier, Université de Montpellier, Montpellier 34295, France; c IDESP, UMR A11-INSERM, Université de Montpellier, Montpellier 34093, France; d ALK-Abellό, Hørsholm, Denmark; e Allergy Unit, Internal Consulting Department, St George University Hospital, Plovdiv, Bulgaria; f Pediatric Clinic, University Hospital in Martin and Jessenius Medical Faculty in Martin, Commenius University, Bratislava, Slovakia; g Department of Pulmonary Diseases and Children Rheumatology, Medical University of Lublin, Lublin, Poland; h Clinique Spécialisée en Allergie de la Capitale, 2600 Boul Laurier, Bureau 880, Quebec, QC G1V 4W2, Canada; i Department of Otorhinolaryngology, Head and Neck Surgery, Section of Rhinology and Allergy, University Hospital Marburg, Philipps-Universität Marburg, Marburg, Germany

**Keywords:** Allergic rhinitis, Allergen immunotherapy, House dust mite, Paediatric, SLIT-tablet

## Abstract

**Background:**

Allergic rhinitis/rhinoconjunctivitis (AR/C) induced by house dust mites (HDM) often begins in childhood and negatively impacts a child’s quality of life. The daily burden can be further compounded by comorbid asthma. Allergen immunotherapy is the only available treatment targeting the underlying cause of allergic disease. Efficacy and safety of the SQ HDM sublingual immunotherapy (SLIT)-tablet has been demonstrated in adults and adolescents with HDM AR/C with or without asthma, but data are lacking for younger children.

**Methods:**

Phase III, randomised, double-blind, placebo-controlled trial in younger children (5–11 years) with HDM AR/C with or without asthma. Eligible subjects were randomised 1:1 to SQ HDM SLIT-tablet or placebo for ∼1 year and had free access to AR/C symptom-relieving medications. The primary outcome was the total combined rhinitis score (TCRS) during the final 8 weeks of the treatment period (∼1 year). Secondary outcomes included the rhinitis daily symptom score (DSS) and medication score (DMS), the rhinoconjunctivitis total combined score (TCS), and the Paediatric Rhinoconjunctivitis Quality of Life Questionnaire (PRQLQ) score. Efficacy analyses were conducted on the full analysis set (observed cases). Asthma-related outcomes were also explored. The trial was registered on ClinicalTrials.gov: NCT04145219 and EudraCT: 2019-000560-22.

**Findings:**

A total of 1460 subjects were randomised to SQ HDM SLIT-tablet (n = 729) or placebo (n = 731). The primary outcome, TCRS, was statistically significantly different for SQ HDM SLIT-tablet (n = 693) versus placebo (n = 706), with an absolute difference of 1.0 (95% CI: 0.5, 1.4; p < 0.0001) corresponding to a relative reduction of 22.0% (95% CI: 12.0, 31.1). Key secondary outcomes (DSS, DMS, TCS, PRQLQ) showed statistically significant reductions in symptoms and medication use, and improved disease-related quality of life for SQ HDM SLIT-tablet versus placebo. Improvements in asthma symptoms and reduced asthma medication use indicated an additional effect of SQ HDM-SLIT tablet versus placebo. The SQ HDM SLIT-tablet showed a higher event rate for treatment-related adverse events (AEs) than placebo. Most events were of mild or moderate severity and few subjects discontinued due to AEs (2.5%).

**Interpretation:**

The trial confirmed the efficacy and safety of the SQ HDM SLIT-tablet for treating HDM AR/C in younger children (5–11 years) with or without asthma. The safety profile supports daily self-administration of the SQ HDM SLIT-tablet in children.

**Funding:**

ALK-Abellό, Hørsholm, Denmark.

**Research in context**

**Evidence before this study**

Symptom-relieving pharmacotherapy (e.g., antihistamine and/or intranasal corticosteroid) is the first-line treatment option for patients suffering from house dust mite (HDM)-induced allergic rhinitis/rhinoconjunctivitis (AR/C). For patients where everyday quality of life remains impaired despite pharmacotherapy, clinical guidelines recommend treatment with allergen immunotherapy (AIT)—the only disease-modifying treatment option for AR/C that targets the underlying cause of allergic disease. According to the literature for phase III trials with SQ HDM sublingual immunotherapy (SLIT)-tablet in HDM AR/C, published from 2016 to 2023 and retrieved from the PubMed database (using the search terms “phase III” OR “trial” AND “house dust mite” AND “SQ SLIT-tablet” OR “SQ sublingual immunotherapy tablet” AND “allergic rhinitis”), the efficacy and safety of SQ HDM SLIT-tablet has been confirmed in four trials involving adults and/or adolescents, and one other trial involving children. However, evidence from large clinical trials of HDM AIT in younger children (5–11 years) is lacking. Consequently, according to clinical guidelines for AIT, there is a need for more clinical evidence from randomised controlled trials demonstrating the efficacy and safety of HDM AIT in younger children with HDM AR/C to support an evidence-based treatment option for those children experiencing impaired quality of life despite the use of symptom-relieving pharmacotherapy.

**Added value of this study**

This phase III trial is the largest double-blind, randomised, placebo-controlled trial of SQ HDM SLIT-tablet in children, and it is also the largest paediatric trial of any HDM AIT product in children published to date. The trial confirmed the efficacy and safety of SQ HDM SLIT-tablet for treating HDM AR/C in younger children (5–11 years) with or without asthma. Statistically significant reductions in clinical symptoms and use of symptom-relieving medications, and improvements in disease-related quality of life, were observed for children treated with SQ HDM SLIT-tablet compared with placebo. These treatment effects were clinically relevant and were achieved in addition to the benefits of existing symptom-relieving pharmacotherapy (subjects had free access to symptomatic treatment throughout the trial). The results from this phase III trial in children complement the existing clinical evidence from previously conducted phase III trials.

**Implications of all the available evidence**

The data generated from this large phase III trial provide the much-needed evidence for the benefits of HDM AIT in children with AR/C that is currently lacking. The confirmed efficacy and safety of the SQ HDM SLIT-tablet in children is important to further support clinical decision-making for treatment with HDM AIT in children, and to inform future AIT guidelines.

## Introduction

Allergic rhinitis (AR) is a common chronic inflammatory disease affecting the upper airways,^1^ and can be accompanied by conjunctivitis (AR/C). The disease often develops early in life with increasing prevalence between childhood and adolescence,^2^ and is triggered by respiratory allergens, such as grass, tree and ragweed pollen, animal dander, and house dust mites (HDM).^1^ Sensitisation to HDM can develop at an early age and is associated with an increased risk of asthma.^3^ Overall, HDM can induce allergic diseases that pose a considerable public health burden, worldwide.^4^

HDM-induced AR/C (HDM AR/C) is usually characterised by persistent symptoms, which are most pronounced during autumn and winter periods.^5^ In an observational study of patients with HDM AR/C, 39.3% (n = 276/702) of children and adolescents reported symptoms that at least moderately impacted their quality of life^6^; symptoms can be experienced as daily physical, social, and emotional challenges.^7^ In particular, AR/C-associated sleep problems have a considerable impact on children^7^ and caregivers through disturbed sleep and subsequent fatigue. Indeed, impaired sleep is one of the main reasons for seeking medical care in two-thirds of children with HDM AR/C.^8^ The effect of HDM allergy is not limited to the upper airways/eyes, but it can also affect the lower airways as allergic asthma,^9^ which has a substantial impact on the burden of disease. An observational study has reported that 42.6% of children with primarily mild or severe persistent HDM AR/C had concomitant asthma.^6^ Furthermore, in children with asthma, the presence of comorbid AR is associated with poor asthma control.^10^

AR/C is managed by avoiding exposure to the allergen(s) causing the symptoms, and/or continuous treatment with symptom-relieving pharmacotherapy, such as antihistamines and intranasal corticosteroids (INCS), to control symptoms.^1^ However, for HDM allergens in particular, the evidence of clinical benefit for various avoidance measures is limited.^1^ Often, despite treatment, patients fail to achieve satisfactory symptom control^5^ and, consequently, quality of life remains impaired. Currently, allergen immunotherapy (AIT) is the only treatment option for AR/C that targets the underlying cause of allergic disease.^11^^,^^12^ AIT has been shown not only to reduce symptoms and the use of symptom-relieving pharmacotherapy and to improve quality of life, but it also has the potential to halt disease progression and prevent the onset of asthma.^12^^,^^13^ Inducing immunological tolerance to the causative allergen is initiated through repeated administration of AIT; for sublingual immunotherapy (SLIT)-tablets, daily administration for 3 years is recommended to achieve a sustained disease-modifying effect.^11^^,^^12^ The onset of clinical effects with AIT is usually observed approximately 2–3 months after initiation, as has been shown in various studies of the SQ HDM SLIT-tablet in adults/adolescents with HDM AR/C.^14^^,^^15^

Efficacy and safety of SQ HDM SLIT-tablet in treating AR/C have been demonstrated in clinical studies in adults and adolescents,^15^, ^16^, ^17^ and in adults with HDM-induced allergic asthma.^18^ Specifically in children, efficacy and safety of SQ SLIT-tablets have been demonstrated for seasonal allergies to different pollen allergens—grass,^19^, ^20^, ^21^ ragweed,^22^ and tree (birch homologous group).^23^ Guidelines from the European Academy of Allergy and Clinical Immunology (EAACI) recommend HDM AIT for AR/C in adults and adolescents, but state that specific paediatric data, especially for younger children, are lacking.^11^ Indeed, for the SQ HDM SLIT-tablet, only one phase III trial in children (aged 5–17 years; n = 458), and a pooled analysis of adolescent (aged 12–17 years; n = 395) data from two phase III trials, have been published.^24^^,^^25^ Consequently, there is a need for more evidence from randomised controlled trials demonstrating the efficacy and safety of HDM AIT in younger children with AR/C.^12^^,^^26^

In clinical practice, the burden of paediatric AR/C and the resulting negative effect of symptoms on a child’s quality of life can be overlooked.^27^ However, awareness is growing,^27^ and there is now greater recognition of the need to initiate treatment early in the disease course.^11^^,^^28^ Early intervention with AIT should be considered as a therapeutic strategy for AR/C and, potentially, as a key preventive approach to reduce the risk of developing asthma.^12^ Achieving such goals would alleviate the burden of allergic disease in children, with subsequent economic benefits through reducing the cost of HDM allergy management. Considering AIT early in the disease course is justified.^11^^,^^28^^,^^29^

This article presents the findings of a large, randomised, double-blind, placebo-controlled, phase III trial conducted to evaluate the efficacy and safety of the SQ HDM SLIT-tablet in younger children (aged 5–11 years) with HDM AR/C with or without asthma.

## Methods

### Study design and participants

The House Dust Mite Allergy Trial In Children (MATIC; also known as MT-12; ClinicalTrials.gov Identifier NCT04145219; EudraCT number 2019-000560-22) was a randomised, parallel-group, double-blind, placebo-controlled, multicentre, phase III trial conducted in 95 sites (hospitals/specialist allergy clinics) across Europe and North America (Appendix p 2). The trial design and conduct were mandated, reviewed, and monitored by European and North American regulatory agencies, and the final trial protocol (Appendix p 8) was approved by both agencies. The trial population comprised younger children aged 5–11 years (at randomisation) with a clinical history of physician-diagnosed HDM AR/C for at least 1 year with or without a clinical history of asthma, and persistent AR symptoms despite receiving symptom-relieving medication. Eligible subjects: 1) had a positive diagnostic test (skin prick test and specific immunoglobulin type E [IgE] test) for HDM sensitisation (*Dermatophagoides pteronyssinus* or *Dermatophagoides farinae*) at screening; 2) had a forced expiratory volume in 1 s (FEV_1_) of at least 70% of predicted value at randomisation; 3) had a rhinitis daily symptom score (DSS) ≥6 points (or ≥5 points with one severe symptom; see Appendix p 3); and 4) were using medication for HDM AR/C during ≥8 of the last 14 days of the baseline period. Subjects with a clinical history of symptomatic non-HDM-related respiratory allergy that potentially could overlap with the baseline and efficacy assessment periods were excluded; this was to avoid confounding, and to ensure optimal data interpretation. If the non-HDM-related symptomatic AR/C was not overlapping with the baseline and/or efficacy assessment periods, the subject was included. Subjects were also excluded if they had a nasal/pharyngeal condition that could interfere with the trial assessments, unstable asthma or asthma requiring >400 μg budesonide or equivalent, daily, and/or a relevant history of systemic allergic reaction. (See Appendix p 5 for the full list of inclusion/exclusion criteria.)

The trial was conducted in accordance with the Declaration of Helsinki and the International Council for Harmonisation of Technical Requirements for Pharmaceuticals for Human Use (ICH) Good Clinical Practice. Relevant ethics committees and national regulatory authorities approved the trial protocol and amendments, plus any other trial documentation. Prior to enrolment, written informed consent was provided by the child’s parents/guardians, and by the child if judicious. An independent Data Monitoring Committee (DMC) monitored trial data to ensure subject safety and trial integrity (data according to treatment allocation [‘A’ or ‘B’] were first generated on May 7, 2020 and reviewed by the DMC on May 18, 2020). Due to the COVID-19 pandemic, recruitment into the first cohort was stopped (ongoing subjects continued), after which an additional recruitment cohort was added to ensure target recruitment (Appendix p 7). Overall, subjects were randomised in three consecutive cohorts; efficacy assessments were conducted in all three cohorts. During the COVID-19 pandemic, subject visits were conducted remotely with no impact on the efficacy assessments.

### Randomisation and masking

Subjects were randomised 1:1 in a double-blind fashion to receive one daily tablet of either SQ HDM SLIT-tablet (12 SQ-HDM dose) or placebo. The SQ HDM SLIT-tablet is a sublingual lyophilisate containing standardised allergen extract from two species of HDM (*D. pteronyssinus and D. farinae*), manufactured by the trial sponsor (ALK-Abellό). Randomisation was stratified by geography and generated by an unblinded trial-independent statistician using random sampling. Subjects/parents/guardians and investigator/site staff/sponsor personnel were blinded to treatment. To maintain blinding, the SQ HDM SLIT-tablet and placebo were similar with regard to appearance, smell, and taste, and were packaged identically. (See the clinical trial protocol in the Appendix [p 8] for full details of randomisation and blinding.)

### Procedures

Following screening and a ∼3-week baseline period, a first dose of SQ HDM SLIT-tablet or placebo was administered under medical supervision at the randomisation visit and the subject was monitored for at least 30 min after intake. Thereafter, one tablet was self-administered daily for approximately 1 year. Compliance with trial medication was assessed at selected visits by SLIT-tablet counts; the aim was 80–100% compliance (calculated as the treatment taken divided by the treatment duration). From baseline, all subjects had free access to symptom-relieving medication (antihistamines [tablets, oral solution, eye drops] and INCS for AR/C, and a short-acting beta agonist [SABA] for asthma), used in accordance with the product label. (See the clinical trial protocol in the Appendix [p 8] for full details of trial medication administration, and of permitted symptom-relieving medications.)

During the trial, AR/C and asthma symptoms and medication use were recorded using an electronic diary (eDiary). Six AR/C symptoms (runny nose, stuffy nose, sneezing, itchy nose, gritty feeling/red/itchy eyes, watery eyes) and four asthma symptoms (chest tightness, wheezing, cough, shortness of breath) were each measured on a scale of 0 (no symptoms) to 3 (severe symptoms). eDiary compliance for the average daily total combined rhinitis score (TCRS) was calculated as the number of daily records of TCRS in the period divided by the expected number of days in the period; the aim was ≥80% compliance.

### Outcomes

The primary efficacy assessment period was the final 8 weeks of the treatment period, which lasted approximately 1 year (Appendix p 107). The primary outcome measure was the average daily TCRS (i.e., the sum of the rhinitis DSS and the rhinitis daily medication score [DMS]). Key secondary outcomes included the average rhinitis DSS, average rhinitis DMS, and the rhinoconjunctivitis average total combined score (TCS; i.e., the sum of the rhinoconjunctivitis DSS and DMS). Briefly, the rhinitis DSS is calculated by combining scores for the four nasal symptoms (runny/stuffy/itchy nose, and sneezing; range 0–12 points); the rhinoconjunctivitis DSS is calculated by also including the scores for the two conjunctivitis symptoms (gritty feeling/red/itchy eyes, and watery eyes; range 0–18 points). Information on the daily use of medication for rhinitis collected in the eDiary was transformed into medication scores to calculate the rhinitis DMS (maximum score 12 points); the rhinoconjunctivitis DMS is calculated by also including the use of medication for rhinoconjunctivitis (maximum score 20 points; higher scores indicate more medication use). Therefore, the daily TCRS ranged from 0 to 24 points, and the daily TCS ranged from 0 to 38 points. (Full details of the scoring algorithms are outlined in the Appendix p 3.)

Additional secondary outcomes included the interview-based Paediatric Rhinoconjunctivitis Quality of Life Questionnaire (PRQLQ) score assessed at the end-of-trial visit (23 questions in five domains; see Appendix p 108 for full details), as well as the average asthma DSS (combined score for chest tightness, wheezing, cough, shortness of breath; range 0–12 points), weekly SABA use (number of puffs), and SABA-free days during the primary efficacy assessment period; also explored were days with nocturnal awakenings due to asthma that required SABA. To evaluate the onset of effect, average daily TCRS, rhinitis DSS, and rhinitis DMS were analysed at Week 8 and Week 16 after treatment initiation, as exploratory outcomes. Immunological outcomes included the change from baseline to the end-of-trial visit in HDM IgE, HDM immunoglobulin type G, class 4 (IgG_4_), and HDM IgE-blocking factor (IgE-BF).

At each subject contact throughout the trial, from screening to up to 3 weeks after the end of trial visit, safety was evaluated through the unsolicited recording of adverse events (AEs) by asking the subject (in an objective manner). Events of special interest (ESIs), such as potential anaphylactic reactions and/or systemic allergic reactions including events requiring treatment with adrenaline, and eosinophilic oesophagitis, were monitored (Appendix p 109). In addition, AEs were collected by solicitation during the first 28 days after treatment initiation through daily completion of the eDiary for 15 pre-specified local allergic reactions (based on recommendations of the World Allergy Organization [WAO]^30^). Pre-specified symptoms/signs captured in the eDiary were evaluated by the investigator and reported as solicited AEs in the subject case report form at the scheduled visit 4 weeks after randomisation (Appendix p 107). All AE reporting during the trial was in accordance with recommendations from the US Food and Drug Administration (FDA) requesting that SLIT-tablet trials use active solicitation to assess WAO-defined local application site reactions.^31^ AE solicitation is known to increase the reporting rate of local allergic reactions in clinical trials.^31^

### Statistical analysis

Full details of the statistical analyses are outlined in the statistical analysis plan (approved May 24, 2023, before database lock and final unblinding of subjects’ treatment allocation; Appendix p 110) and summarised briefly here. The statistical analysis plan (Appendix p 110) was approved by European and North American regulatory agencies. Based on the effect sizes of previous SQ HDM SLIT-tablet trials and assuming a 15% dropout rate, the trial planned to randomise 1370 subjects (685 in each treatment arm) to ensure 90% power to detect superiority of SQ HDM SLIT-tablet versus placebo (plus free access to symptom-relieving medication) at the 5% level of significance for the primary efficacy analysis. The power calculation was based on the point estimate of the relative treatment difference in TCRS (active minus placebo/placebo). The trial was powered for the minimal clinically relevant difference of no more than −15% with an associated upper two-sided 95% confidence limit being −10% at most, which is in line with the FDA considerations on clinically relevant efficacy^32^ (and agreed by the FDA during the scientific advice process to inform the MT-12 trial design) (see the clinical trial protocol in Appendix p 8). There is no defined minimal clinically relevant difference for the secondary endpoints.

Efficacy analyses were conducted on observed data for subjects in the full analysis set (FAS; defined as all randomised subjects who received at least one dose of trial medication) who had at least one eDiary record during the relevant assessment period (observed case analysis). Each outcome was calculated as the average of all the observed daily values during the relevant period. The primary and key secondary efficacy outcomes were analysed using a t-test within a linear mixed effects (LME) model, with square root transformation of the scores during the primary efficacy assessment period as response variable, treatment group and cohort as fixed effects, square root of the average baseline score as a covariate, and country/region within cohort as random effect; different residual errors were specified for each treatment group. The square root transformation was applied to fulfil the assumption of normally distributed residuals and chosen as transformation to handle zero values, which is a common approach for this type of data. For the relative difference, Fieller’s theorem was used to calculate the 95% CI as a first step, then the 95% CI was back transformed by applying a monotone transformation. For the adjusted means and absolute difference, the standard error was approximated by using the first order Delta method and from this, the 95% CI was calculated. The between-group difference in the overall PRQLQ score was analysed similarly to the primary endpoint using the LME model, but without square root transformation. The primary and key secondary efficacy outcomes, and the PRQLQ score at the end of the trial, were controlled for multiplicity using hierarchical testing for superiority of the SQ HDM SLIT-tablet over placebo, using a 0.05 level of significance, following the order: 1) TCRS; 2) rhinitis DSS; 3) rhinitis DMS; 4) rhinoconjunctivitis TCS; 5) PRQLQ. Subgroup analyses were conducted for the primary efficacy outcome by baseline asthma status (asthma yes/no; pre-defined analysis), and by baseline sensitisation status (to HDM only, or to HDM and other allergens; post-hoc analysis). All statistical tests were performed at the 5% significance level, and all tests and CIs were two-sided. Data are presented as adjusted means (standard error [SE]), absolute treatment difference between SQ HDM SLIT-tablet versus placebo with 95% CI and p-value, and relative treatment difference between the two groups with 95% CI. The primary and key secondary outcome analyses were supplemented with analyses using the estimand framework, as well as sensitivity analyses. All statistical analyses were conducted by the study sponsor using SAS (version 9.4; SAS Institute, Cary, NC).

Serum concentrations for HDM IgE and HDM IgG_4_ (but not HDM IgE-BF) were log_10_ transformed, and the difference in the change from baseline to the end-of-trial visit between the SQ HDM SLIT-tablet and placebo was analysed post hoc using an LME model (treatment and cohort as fixed effects, baseline value as a covariate, and different residual errors specified for each treatment); data are presented as means with 95% CI. Safety analyses were conducted on the safety analysis set, defined as all randomised participants who received at least one dose of study medication; the data are summarised descriptively.

### Role of the funding source

This trial was funded by ALK-Abellό, Hørsholm, Denmark, which assumes overall responsibility for the trial design and trial conduct (including the collection of data), the statistical analyses and data interpretation, the writing of the report, and for the decision to submit the paper for publication.

## Results

**Fig. 1:**
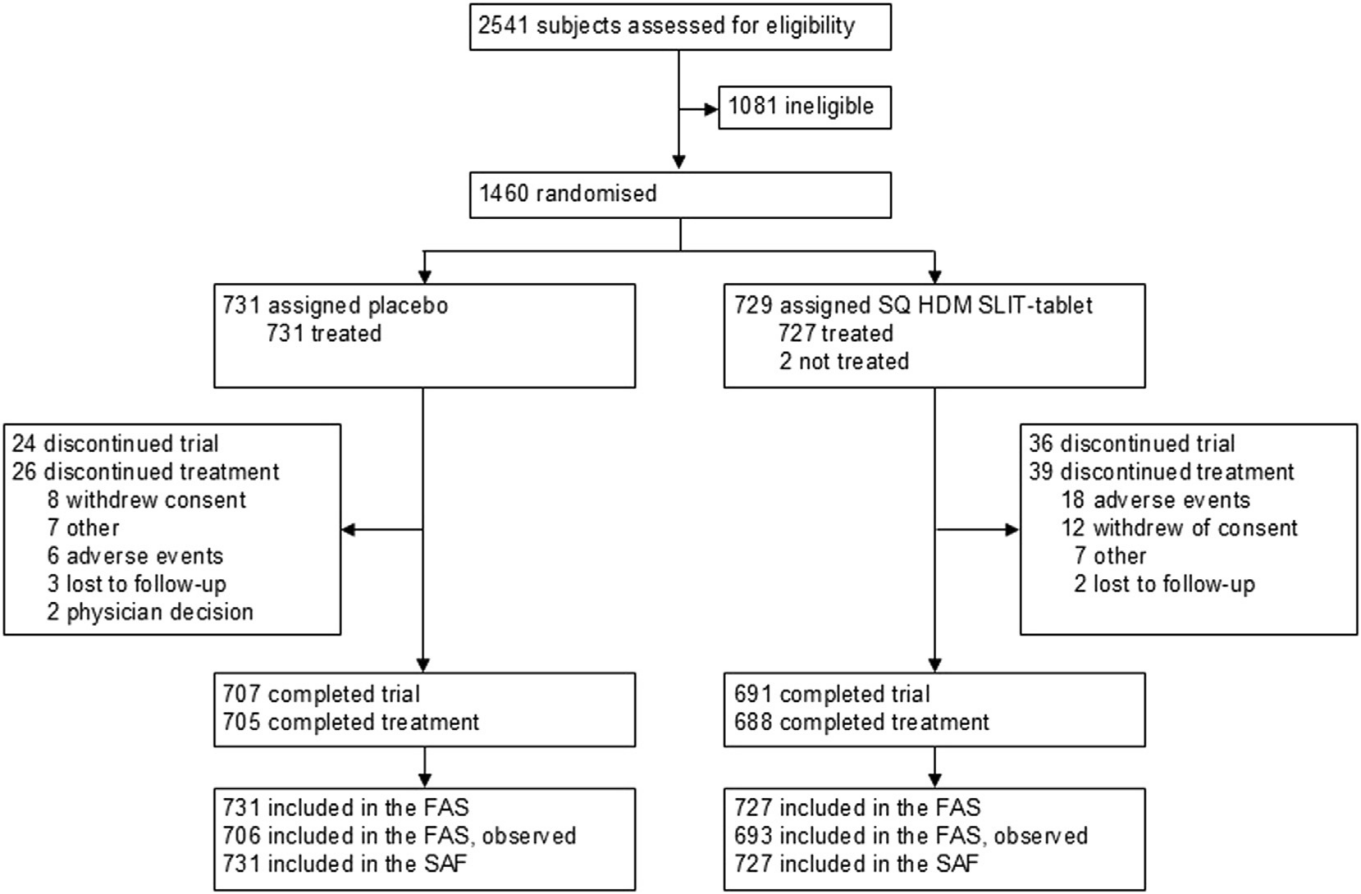
Subject flow throughout the trial. FAS = full analysis set. FAS, observed = FAS with an observed assessment for the primary efficacy outcome. HDM = house dust mite. SAF = safety analysis set. SLIT = sublingual immunotherapy.

Subjects were recruited between September 29, 2019, and April 1, 2022. A total of 1458 subjects were randomised and treated with SQ HDM SLIT-tablet or placebo. During the trial, 95.5% (n = 1393) of subjects completed treatment, and 95.9% of subjects (n = 1398) completed the trial (Fig. 1). The mean treatment compliance was >95.0%, and the mean TCRS eDiary compliance was >94.0%; compliance was similar between treatment groups. Overall, the discontinuation rate due to AEs was low in the two treatment groups.

Baseline demographics and clinical characteristics were balanced across the SQ HDM SLIT-tablet and placebo groups (Table 1; additional parameters are listed in Appendix p 161). The mean age of subjects was 8 years; the mean duration of HDM AR/C was approximately 2.8 years, and 61.2% (n = 893/1458) of subjects had AR at baseline while 38.7% (n = 564/1458) had allergic rhinoconjunctivitis (ARC) (Table 1). The majority of subjects (90.6%; n = 1321/1458) were using antihistamines plus INCS as symptom-relieving medication for AR. More than half of the subjects (52.2%; n = 761/1458) were polysensitised, and 38.2% (n = 557/1458) of subjects had asthma (mainly HDM-induced asthma, intermittent or mild, and over half of those subjects were using inhaled corticosteroids [ICS]).

**Table 1:** Baseline demographics and clinical characteristics (FAS).

	Placebo (N = 731)	SQ HDM SLIT-tablet (N = 727)
Demographics		
Age, years		
Mean (SD)	8.0 (1.9)	8.0 (1.9)
Median (min–max)	8.0 (4–11)	8.0 (4–11)
Male^a^	477 (65.3%)	486 (66.9%)
White	714 (97.7%)	722 (99.3%)
Clinical characteristics		
Mean (SD) duration of HDM AR/C, years	2.8 (1.8)	2.7 (1.8)
Allergy history		
HDM AR	440 (60.2%)	453 (62.3%)
HDM ARC	291 (39.8%)	273 (37.6%)
HDM AR or ARC	731 (100.0%)	726 (99.9%)^b^
Use of medication for AR		
Antihistamines	26 (3.6%)	25 (3.4%)
INCS	43 (5.9%)	43 (5.9%)
Antihistamines + INCS	662 (90.6%)	659 (90.6%)
Sensitisation status		
Monosensitised (HDM only)	360 (49.2%)	337 (46.4%)
Polysensitised (HDM plus other allergens)	371 (50.8%)	390 (53.6%)
AR/C symptom and medication score,^c^ mean (SD)		
TCRS	18.3 (3.3)	18.4 (3.2)
Rhinitis DSS	8.0 (1.7)	8.1 (1.7)
Rhinitis DMS	10.3 (2.4)	10.3 (2.3)
Rhinoconjunctivitis TCS	22.6 (5.6)	22.5 (5.4)
PRQLQ score		
Mean (SD)	2.4 (1.1)^d^	2.4 (1.0)
Median (min–max)	2.4 (0.3–5.5)	2.4 (0.0–5.0)
FEV_1_, % predicted, median (min–max)	96.8% (70–179)	97.1% (58–193)^e^
Asthma	290 (39.7%)	267 (36.7%)
HDM-driven asthma	260 (89.7%)	236 (88.4%)
ICS use	160 (55.2%)	148 (55.4%)
Asthma severity^f^		
Mild (including intermittent^g^)	212 (73.1%)	194 (72.7%)
Moderate	76 (26.2%)^h^	73 (27.3%)
Asthma DSS, mean (SD)	2.0 (2.3)	2.3 (2.5)

Data are n (%), unless otherwise stated.

AR = allergic rhinitis. AR/C = allergic rhinitis with or without conjunctivitis. ARC = allergic rhinoconjunctivitis. DMS = daily medication score. DSS = daily symptom score. eCRF = electronic case report form. FAS = full analysis set. FEV_1_ = forced expiratory volume in 1 s. HDM = house dust mite. ICS = inhaled corticosteroid. INCS = intranasal corticosteroid. LABA = long-acting beta agonist. N = number of subjects in the FAS. NAEPP = National Asthma Education and Prevention Program. PRQLQ = Paediatric Rhinoconjunctivitis Quality of Life Questionnaire. SABA = short-acting beta agonist. SD = standard deviation. SLIT = sublingual immunotherapy. TCRS = total combined rhinitis score. TCS = total combined score.

a Sex of subjects was reported by the investigator.

b All subjects had rhinitis or rhinoconjunctivitis at baseline; one subject included in the trial had ‘rhinitis’ entered in the wrong eCRF form.

c Scoring: TCRS, 0–24 points; rhinitis DSS, 0–12 points; rhinitis DMS, maximum 12 points; rhinoconjunctivitis TCS, 0–38 points.

d n = 730.

e One non-asthmatic subject below 7 years of age in the SQ HDM SLIT-tablet group reported an FEV_1_ value of 58%, which is lower than the protocol-defined inclusion criteria (FEV_1_ ≥ 70% of predicted value at randomisation). However, the protocol states that this criterion is not required for subjects without asthma who are 7 years or younger, if they cannot accurately perform the procedure, despite coaching.

f Asthma severity was graded according to the use of asthma controller medication (ICS, LABA) or SABA use at baseline, adapted from the NAEPP guidelines.^33^

g Subjects who used SABA only as asthma reliever medication at baseline were considered to have intermittent asthma.

h Two subjects using medium-dose ICS were erroneously reported (in the eCRF) as using high-dose ICS and are not included in the table.

**Fig. 2:**
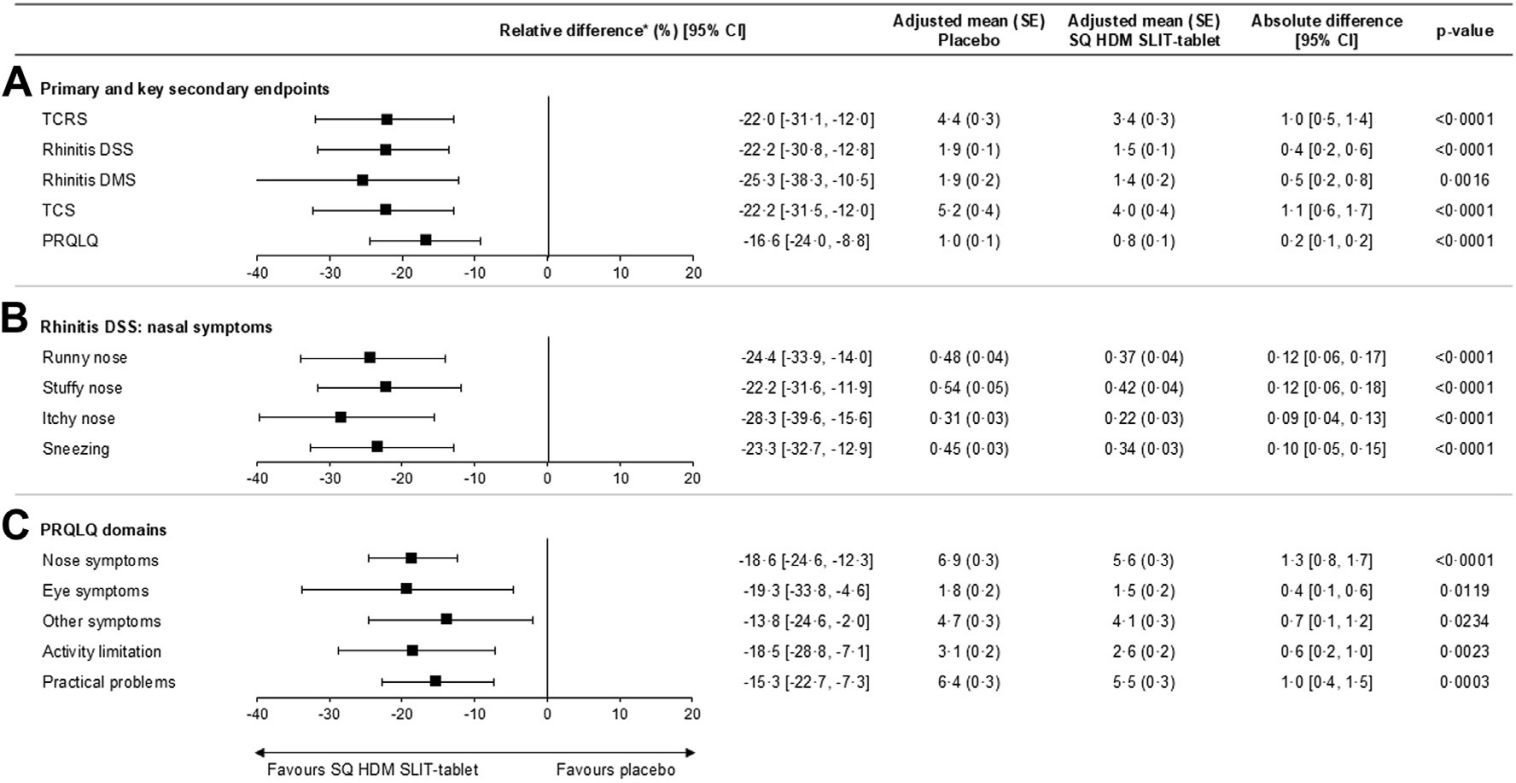
Key efficacy outcomes for the SQ HDM SLIT-tablet versus placebo (FAS, observed case analysis). (A) Primary and secondary efficacy outcomes assessed in the hierarchical statistical testing (primary endpoint: TCRS; key secondary endpoints: rhinitis DSS and rhinitis DMS; secondary endpoint: PRQLQ). (B) Individual nasal components of the rhinitis DSS. (C) Individual PRQLQ domains. TCRS, rhinitis DSS, rhinitis DMS, and TCS were assessed during the 8-week primary efficacy assessment period; PRQLQ was assessed at the end of the trial. The FAS included 731 subjects in the placebo group and 727 subjects in the SQ HDM SLIT-tablet group. Number of subjects included in the FAS, observed case analysis, for TCRS, DSS, DMS, and TCS: placebo, n = 706; SQ HDM SLIT-tablet, n = 693. Number of subjects included in the FAS, observed case analysis for PRQLQ: placebo, n = 690; SQ HDM SLIT-tablet, n = 695. ∗Calculated as SQ HDM SLIT-tablet minus placebo divided by placebo. CI = confidence interval. DMS = daily medication score. DSS = daily symptom score. FAS = full analysis set. HDM = house dust mite. n = number of subjects in the FAS with data contributing to the analysis. PRQLQ = Paediatric Rhinoconjunctivitis Quality of Life Questionnaire. SE = standard error. SLIT = sublingual immunotherapy. TCRS = total combined rhinitis score. TCS = total combined score.

Hierarchical testing for the average daily TCRS (primary outcome), the rhinitis DSS, rhinitis DMS, rhinoconjunctivitis TCS (key secondary outcomes), and the PRQLQ (secondary outcome) showed that the absolute treatment difference in the average daily TCRS for the SQ HDM SLIT-tablet versus placebo was 1.0 (95% CI: 0.5, 1.4; p < 0.0001), which corresponds to a relative reduction of 22.0% (95% CI: 12.0, 31.1) (Fig. 2). For the other outcomes, the SQ HDM SLIT-tablet statistically significantly reduced symptoms and medication use, and improved disease-related quality of life, versus placebo (plus free access to symptom-relieving medication) (Fig. 2). Sensitivity and estimand analyses consistently provided similar results to the primary and key secondary outcomes (Appendix p 162).

The statistically significantly lower average daily TCRS for the SQ HDM SLIT-tablet over placebo was consistent across subgroups of subjects with and without asthma at baseline, and of subjects who were monosensitised (to HDM) and polysensitised (to HDM and other allergens) at baseline (Supplementary Fig. S2; Appendix page 163).

**Fig. 3:**
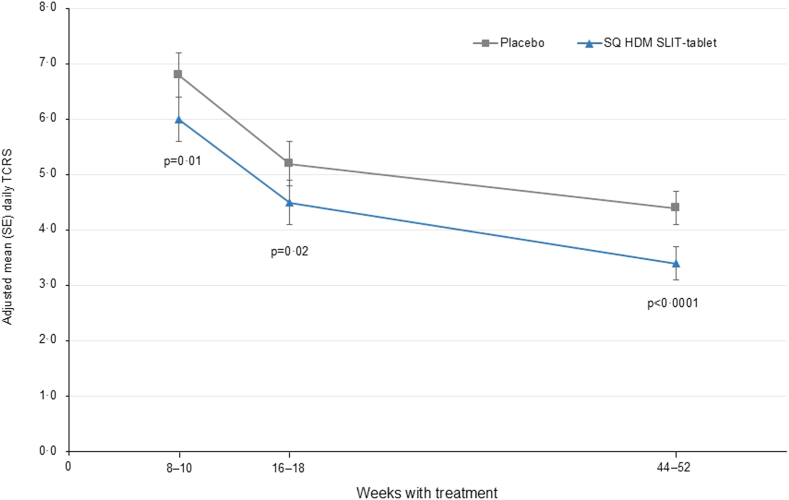
Mean daily TCRS over time (FAS, observed case analysis). Baseline TCRS scores, mean (SD): placebo, 18.3 (3.3); SQ HDM SLIT-tablet, 18.4 (3.2). p-values reflect the between-group differences. The FAS included 731 subjects in the placebo group and 727 subjects in the SQ HDM SLIT-tablet group. Number of subjects included in the FAS, observed case analyses: Week 8—placebo n = 705, SQ HDM SLIT-tablet n = 695; Week 16—placebo n = 709, SQ HDM SLIT-tablet n = 690; Weeks 44–52—placebo n = 706, SQ HDM SLIT-tablet n = 693. TCRS was calculated as an average over a 2-week efficacy assessment period corresponding with Week 8 and Week 16, and during the primary efficacy assessment period, approximately Weeks 44–52 (primary outcome). FAS = full analysis set. HDM = house dust mite. n = number of subjects in the FAS with data contributing to the analysis. SD = standard deviation. SE = standard error. SLIT = sublingual immunotherapy. TCRS = total combined rhinitis score.

The TCRS for the SQ HDM SLIT-tablet was statistically significantly lower than placebo from Week 8 (absolute treatment difference of 0.8 [95% CI: 0.2, 1.5]; p = 0.01; relative difference of 12.0% [95% CI: 3.0, 20.4) onwards (Fig. 3; Appendix p 164). Similar statistically significant results were consistently observed for the SQ HDM SLIT-tablet versus placebo from Week 8 on all the key secondary outcomes—rhinitis DSS (absolute treatment difference of 0.3 [95% CI: 0.1, 0.6]; p = 0.02), rhinitis DMS (absolute treatment difference of 0.5 [95% CI: 0.0, 0.9]; p = 0.03), and rhinoconjunctivitis TCS (absolute treatment difference of 1.0 [95% CI: 0.2, 1.7]; p = 0.01) (Appendix p 164).

For all individual nasal symptoms, treatment with the SQ HDM SLIT-tablet showed consistent statistically significantly lower scores versus placebo, with relative treatment reductions greater than 22.0% (Fig. 2). PRQLQ scores were also statistically significantly lower for the SQ HDM SLIT-tablet group versus the placebo group—overall and for each individual domain—indicating a better disease-related quality of life at the end of the trial with the SQ HDM SLIT-tablet (Fig. 2).

For the four asthma-related secondary outcomes (assessed in subjects with asthma at baseline)—asthma DSS, weekly use of SABA, SABA-free days, and nocturnal awakenings due to asthma requiring SABA use—the data were in favour of the SQ HDM SLIT-tablet; statistically significant benefits over placebo were observed for the asthma DSS and nocturnal awakenings requiring SABA use (Appendix p 165).

Treatment with the SQ HDM SLIT-tablet for approximately 1 year resulted in treatment-induced changes in relevant immune parameters (HDM IgE, HDM IgG_4_, and HDM IgE-BF) that were statistically significantly different from results in the placebo group (p < 0.0001; Appendix p 166).

Generally, the SQ HDM SLIT-tablet showed a favourable safety profile. Treatment-related AEs (TRAEs) (i.e., treatment-emergent AEs [TEAEs] reported as ‘possibly’ related to trial medication by the investigator) were reported in >50% of subjects in each group, and with a higher frequency for the SQ HDM SLIT-tablet than for placebo (Table 2). However, few TRAEs resulted in treatment discontinuation—1.8% (n = 13/727) of subjects in the SQ HDM SLIT-tablet group discontinued due to TRAEs (Table 2). In both treatment groups, all TRAEs were non-serious, and most were mild in severity; most subjects recovered and did not require a change in trial medication (Table 2). For 95.4% of events, TRAEs were solicited AEs reported during the first 28 days of treatment. A similar pattern was noted for TEAEs—there was a slightly higher frequency with the SQ HDM SLIT-tablet and, in both groups, a low rate of consequent treatment discontinuations, events were mostly mild in severity, few serious events were reported, and most subjects recovered without medication changes (see Appendix p 168 for an overall summary of TEAEs).

**Table 2:** Summary of TRAEs (safety analysis set).

	Placebo (N = 731)		SQ HDM SLIT-tablet (N = 727)	
	Number of subjects	Number of events	Number of subjects	Number of events
All events	391 (53.5%)	2183 (100.0%)	548 (75.4%)	5220 (100.0%)
Severity				
Mild	379 (51.8%)	2068 (94.7%)	542 (74.6%)	4894 (93.8%)
Moderate	47 (6.4%)	113 (5.2%)	96 (13.2%)	314 (6.0%)
Severe	2 (0.3%)^a^	2 (<0.1%)^a^	4 (0.6%)^b^	12 (0.2%)^b^
Serious	0 (0.0%)	0 (0.0%)	0 (0.0%)	0 (0.0%)
Outcome				
Recovered/resolved	391 (53.5%)^c^	2182 (100.0%)	548 (75.4%)^c^	5216 (99.9%)
Not recovered/not resolved	0 (0.0%)	0 (0.0%)	3 (0.4%)	3 (<0.1%)
Unknown	1 (0.1%)^d^	1 (<0.1%)	1 (0.1%)	1 (<0.1%)
Changes to trial medication due to TRAE^e^				
None	381 (52.1%)	2143 (98.2%)	545 (75.0%)	5109 (97.9%)
Trial medication interrupted	23 (3.1%)	32 (1.5%)	36 (5.0%)	82 (1.6%)
Trial medication withdrawn	7 (1.0%)	8 (0.4%)	13 (1.8%)	29 (0.6%)

TRAEs are TEAEs reported as ‘possibly’ related to trial medication by the investigator.

HDM = house dust mite. N = number of subjects in the safety analysis set. SLIT = sublingual immunotherapy. TEAE = treatment-emergent adverse event. TRAE = treatment-related adverse event.

a Abdominal pain (one event in one subject) and rhinitis allergic (one event in one subject).

b One subject experienced three events of oral pruritus, three events of ear pruritus, and two events of glossodynia; one subject experienced two events of lip swelling; one subject experienced one event of abdominal pain; one subject experienced one event of lip swelling.

c Percentages calculated from the total number of subjects (placebo, N = 731; SQ HDM SLIT-tablet, N = 727).

d One subject experienced two events with different outcomes (e.g., one event recovered/resolved, and one event with unknown status).

e Some subjects reported several events, each with different outcomes in relation to medication changes (e.g., some events required no change, some required treatment interruption, and some required treatment withdrawal).

The three most frequent TRAEs (i.e., reported in at least 2.0% of subjects in the SQ HDM SLIT-tablet group) were local application–site reactions, including oral pruritus, throat irritation, and ear pruritus (Appendix p 169). The TEAE profile was similar, showing that local application–site reactions were most commonly reported, albeit with a higher frequency in the SQ HDM SLIT-tablet group than in the placebo group (Appendix p 170). Generally, the most common TRAEs occurred at the beginning of treatment and resolved within a few days (median duration: 1–2 days) (Appendix p 169). The ESI rate was very low (0.5%; n = 8/1458). Systemic allergic reactions were reported by three subjects in the SQ HDM SLIT-tablet group and two subjects in the placebo group—all events were mild or moderate in severity and were not considered serious (see Appendix p 171 for narratives of all events). For the SQ HDM SLIT-tablet, two events were considered possibly related to treatment: symptoms of mild urticaria on chest and abdomen (one subject; treatment unchanged), and of moderate hypersensitivity with symptoms of epigastric pain and malaise (one subject; treatment was discontinued). No event was treated with adrenaline, and all subjects with systemic allergic reactions fully recovered. No event of eosinophilic oesophagitis was reported. No clinically relevant changes in laboratory parameters, vital signs, physical examinations, or lung function were observed in either treatment group during the trial, and no deaths were reported.

## Discussion

This dedicated paediatric phase III trial is the largest randomised, double-blind, placebo-controlled trial of the SQ HDM SLIT-tablet in children, and it is also the largest such investigation of HDM AIT in children published to date. The trial enrolled a clinically relevant population of children with moderate-to-severe HDM AR/C reflected by high symptom scores and medication use alongside quality of life impairment at baseline. Being a well-conducted clinical study with a high compliance and completer rate, the trial confirms the efficacy and safety of the SQ HDM SLIT-tablet in children with HDM AR/C with or without asthma. The SQ HDM SLIT-tablet consistently demonstrated a clinically relevant effect on reducing AR/C symptoms and medication use, and improving disease-related quality of life, across all key efficacy outcomes (including individual symptoms and quality of life domains). The effect was similar across subgroups of mono- or polysensitised subjects, and positive treatment effects were observed for subjects with and without asthma.

In relative terms, the difference of at least 22% between the SQ HDM SLIT-tablet and placebo groups observed across the primary and all key secondary outcomes exceeds the recommendation for a clinically relevant difference of 20% according to the WAO.^34^ In line with other SQ HDM SLIT-tablet trials,^16^ clinical relevance was also achieved according to the protocol-defined recommendations from the FDA for a relative treatment difference versus placebo of ≥ 15% (with a lower bound 95% CI of ≥ 10%). Together, these findings confirm the clinically relevant effect of the SQ HDM SLIT-tablet to improve symptoms and reduce medication use in children with HDM AR/C with or without asthma. Furthermore, our present data show comparable effect sizes to previous SQ HDM SLIT-tablet trials in subjects with HDM AR/C (with or without asthma)—reductions in TCRS of 20% in a European trial (adults),^15^ 17% in a North American trial (adults and adolescents),^16^ and up to 24% in two Japanese trials (children, adolescents, and adults).^24^^,^^35^ The data are also comparable to the findings from paediatric trials of SQ SLIT-tablets for specific seasonal pollen allergies (grass, ragweed, and tree [birch homologous group]),^19^, ^20^, ^21^, ^22^, ^23^ as well as relevant trials of other SLIT-tablet formulations for HDM and grass AR/C.^36^^,^^37^ Most recently, evidence for a consistent effect of SLIT-tablets across allergens (HDM, and pollen from grass, tree, and ragweed), geographic regions (North America, Europe, and Japan), and age ranges (children, adolescents, and adults), has been published.^38^ The onset of effect for the SQ HDM SLIT-tablet was statistically significant from 8 weeks following treatment initiation, consistent with the findings from the controlled HDM chamber exposure study in adults.^14^ The effect continued to increase over time and was maintained when assessed approximately 1 year after starting treatment. It is important to note that the observed treatment effect reported for the SQ HDM SLIT-tablet versus placebo is in addition to the benefits of AR/C symptom-relieving medications. Considering the high burden of disease at baseline as reflected in the TCRS, according to our results, clinicians can expect to see a marked improvement over time in both clinical symptoms and medication use when children with HDM AR/C are treated with the SQ HDM SLIT-tablet in addition to symptom-relieving medication. The reduction in symptom and medication scores observed in the placebo group aligns with the findings of previous placebo-controlled AIT trials where the improvement in the placebo group is 23–68%.^15^^,^^16^^,^^37^^,^^39^ In the present (and previous) trials, subjects were permitted to use symptom-relieving medication and were trained in using the eDiary to monitor symptoms and medication use. Hence, subject awareness and compliance with the trial protocol are possible reasons for the observed symptomatic improvement in the placebo group. An effect during placebo treatment is a recognised phenomenon in such studies and can be further influenced by regression to the mean, changes in patient behaviour (i.e., the Hawthorne effect^40^), patient expectation of the benefit of a specific treatment, or by using patient-reported endpoints^39^ (e.g., based on clinical symptoms and medication use) that provides closer patient follow up. Consequently, it is important to conduct randomised, double-blind, placebo-controlled trials to establish the safety and efficacy of AIT products, especially in paediatric clinical development programmes.^41^

In support of the efficacy findings, subjects receiving the SQ HDM SLIT-tablet also showed significant improvements in rhinoconjunctivitis-related quality of life versus placebo. The absolute differences observed are in line with the quality-of-life data from previous SQ HDM SLIT-tablet trials,^15^^,^^16^ and from a paediatric trial of the SQ grass SLIT-tablet.^21^ In the present trial, treatment with the SQ HDM SLIT-tablet showed a significant benefit over placebo in alleviating the negative impact of nasal symptoms on quality of life and in reducing limitations to important daily activities, across all quality-of-life domains, including sleep (reflected in the activity limitation domain of the PRQLQ; Appendix p 108). Currently, the minimal important difference (MID) in PRQLQ score is undefined for HDM AIT trials. However, data for the adult version of the Rhinoconjunctivitis Quality of Life Questionnaire (RQLQ) may provide a helpful substitute. Based on RQLQ data from adult trials of SQ grass and SQ tree SLIT-tablets, Blaiss and colleagues^42^ published values for the between-group differences in RQLQ score that represent a clinical meaningful change for grass and tree pollen allergy—the values were 0.22 and 0.26, respectively, for the entire pollen season.^42^ In the present trial, the absolute difference between the SQ HDM SLIT-tablet and placebo groups was 0.2 points in overall PRQLQ score—in line with the published values for the between-group MID on the RQLQ. However, it is uncertain whether the MID for RQLQ in seasonal allergies is directly comparable to PRQLQ in perennial HDM AR/C and also whether the data from adults can be extrapolated to paediatric patients.

The MT-12 trial was designed to evaluate the efficacy of the SQ HDM SLIT-tablet in children with HDM AR/C, primarily. Children with a clinical history of concomitant asthma were also permitted and, consequently, the trial was limited in that it was not designed to demonstrate efficacy in the asthma subgroup. A limitation was that most children with concomitant asthma had mild or intermittent asthma (with SABA use only) and a low mean asthma DSS at baseline, indicating low disease severity; consequently, observable effect sizes for the asthma outcomes showed relatively small numerical benefits for the SQ HDM SLIT-tablet versus placebo across all asthma-related outcomes (asthma DSS, SABA use, nocturnal awakenings). Two previous trials of the SQ HDM SLIT-tablet in patients with HDM allergic asthma have been conducted showing benefits on asthma-related outcomes. The first trial recruited adults and adolescents (aged ≥ 14 years) with HDM AR and mild-to-moderate asthma, and reported a moderate statistically significant reduction in the SQ HDM SLIT-tablet versus the placebo group regarding the dose of ICS required to maintain asthma control.^43^ The second trial recruited adults with HDM AR and asthma not well controlled by ICS or combination products, and reported that the addition of SQ HDM SLIT-tablet treatment to maintenance medications reduced the risk of moderate-to-severe asthma exacerbations for SQ HDM SLIT-tablet versus placebo.^18^

The SLIT-tablet releases the causative allergen under the tongue and, therefore, local application–site reactions are expected and, indeed, are common adverse drug reactions. Safety analyses showed that, local application–site reactions were the most common TRAEs of the SQ HDM SLIT-tablet in children with HDM AR/C with or without asthma. Local application–site reactions usually occurred in the first week after the start of treatment and resolved within a few days. Overall, the safety findings are in accordance with the established safety profile for the SQ HDM SLIT-tablet in adolescents and adults,^15^^,^^16^ and similar to the safety profile reported in other paediatric SQ SLIT-tablet trials.^19^, ^20^, ^21^, ^22^

The key strengths of the trial lie in its design and in the consistency of outcomes. Almost 1500 children were randomised, making it the largest paediatric trial of the SQ HDM SLIT-tablet conducted to date and, currently, the largest randomised controlled trial of AIT in children. In addition, safety and tolerability were adequately assessed given that all subjects treated were included in the safety analyses. Furthermore, the data generated from this trial provide the much-needed evidence for the benefits of HDM AIT in children with AR/C that is currently lacking, and which can be used to inform clinical guidelines. A limitation of the analysis is that the trial was not designed to confirm efficacy on asthma. However, the data indicate a favourable safety profile for the SQ HDM SLIT-tablet in subjects with or without asthma as a comorbidity. Further investigation into asthma is warranted.

The SQ HDM SLIT-tablet is an important treatment for managing HDM AR/C in children for whom current management is insufficient to control symptoms, complete allergen avoidance is unrealistic, and quality of life often remains impaired despite treatment with symptom-relieving medications. AIT is a causal treatment with the potential to halt disease progression,^12^^,^^13^ which is particularly relevant for HDM AR/C where the progression to asthma is a serious concern.

In summary, this large, double-blind, placebo-controlled trial of SQ HDM SLIT-tablet in children aged 5–11 years confirms its efficacy and safety for treating HDM AR/C with or without asthma, based on the statistically significant and clinically relevant improvement in symptoms and medication use, and in disease-related quality of life. The safety profile of the SQ HDM SLIT-tablet in children was similar to the established safety profile in adults and adolescents, and supports daily self-administration in children.

## Contributors

All authors contributed to the study concept and design, and/or to the acquisition, analysis, or interpretation of data. All authors had access to the trial data, reviewed the manuscript, revised the content, and approved the final version for submission.

Professional medical writing and editorial support was provided by Emma Court PhD and colleagues within Cambridge—a division of Prime (Knutsford, UK), according to Good Publication Practice guidelines, and funded by ALK-Abelló.

## Data sharing statement

The study protocol and statistical analysis plan have been submitted with the manuscript. Further requests for data relating to the MT-12 study can be made through the ALK-Abelló website, ‘Sharing our clinical trials data’ at https://www.alk.net/our-science/clinical-data-sharing.

## Declaration of interests

AS has received personal fees/clinical study fees from ALK-Abelló. Unpaid board member for German paediatric scientific societies (Gesellschaft für Pädiatrische Pneumologie [GPP] and Westdt. AG für pädiatrische Pneumologie und Allergologie [WAPPA]; unpaid co-author on scientific guidelines.

DC has received fees for oral presentations from ALK-Abelló, Stallergenes Greer, AstraZeneca, GlaxoSmithKline, and Sanofi-Genzyme. Scientific board member for ALK-Abelló, Stallergenes Greer, AstraZeneca, and Sanofi-Genzyme.

HN is an employee of ALK-Abelló.

SN has received fees for oral presentations from Stallergenes Greer, TEVA. Chie, Berlin–Chemie, and Chiesi.

JM was a clinical investigator in clinical studies MT-12, MT-18, and TT-06 sponsored by ALK-Abelló, and YOBI-SL79.22 sponsored by Stallergenes.

MHF-S is an employee of ALK-Abelló.

ASØ was an employee of ALK-Abelló when the work was conducted, but has since moved to Novo Nordisk.

AE has received lecture fees from ALK-Abelló.

RG has conducted research for ALK-Abelló, AstraZeneca, GSK, DBV, Regeneron, Moderna, Novartis, and Sanofi, and has received consultancy fees from ALK-Abelló, Regeneron, Bausch Lomb, and Sanofi-Genzyme; received lecture fees from Bausch Lomb, and Novartis.

OP reports grants from ALK-Abelló, Denmark, during the conduct of the study; furthermore, he reports grants and/or personal fees and/or travel support from ALK-Abelló, Allergopharma, Stallergenes Greer, HAL Allergy Holding B.V./HAL Allergie GmbH, Bencard Allergie GmbH/Allergy Therapeutics, Laboratorios LETI/LETI Pharma, GSK, ROXALL Medizin, Novartis, Sanofi-Aventis and Sanofi-Genzyme, Med Update Europe GmbH, streamedup! GmbH, Pohl-Boskamp, Inmunotek S.L., John Wiley and Sons/AS, Paul-Martini-Stiftung (PMS), Regeneron Pharmaceuticals Inc., RG Aerztefortbildung, Institut für Disease Management, Springer GmbH, AstraZeneca, IQVIA Commercial, Ingress Health, Wort&Bild Verlag, Verlag ME, Procter&Gamble, ALTAMIRA, Meinhardt Congress GmbH, Deutsche Forschungsgemeinschaft, Thieme, Deutsche AllergieLiga e.V., AeDA, Alfried-Krupp Krankenhaus, Red Maple Trials Inc., Königlich Dänisches Generalkonsulat, Medizinische Hochschule Hannover, ECM Expo & Conference Management GmbH, Technical University Dresden, Lilly, Japanese Society of Allergy, Forum für Medizinische Fortbildung, Dustri-Verlag, Pneumolive, ASIT Biotech, LOFARMA, Almirall, Paul-Ehrlich-Institut, all outside the submitted work; and he is member of EAACI Excom, member of ext. board of directors DGAKI; coordinator, main- or co-author of different position papers and guidelines in rhinology, allergology and allergen-immunotherapy; he is associate editor (AE) of *Allergy* and *Clinical Translational Allergy*.
