# Supplementary material for: Efficacy and safety of SQ house dust mite sublingual immunotherapy-tablet (12 SQ-HDM) in children with allergic rhinitis/rhinoconjunctivitis with or without asthma (MT-12): a randomised, double-blind, placebo-controlled, phase III trial
Source: Lancet Reg Health Eur. 2024 Nov 26;48:101136. doi: 10.1016/j.lanepe.2024.101136 (PMC11638617; doi:10.1016/j.lanepe.2024.101136)
Supplement: Supplementary Figs. S1–S3 and Tables S1–S13 [file mmc1.pdf]

SUPPLEMENTARY APPENDIX

Efficacy and safety of SQ house dust mite sublingual immunotherapy-tablet (12 SQ-HDM) in children with allergic rhinitis/rhinoconjunctivitis with or without asthma: a randomised, double-blind, placebo-controlled, phase III trial

Table of contents

Table S1: Site information	**2**
Table S2: Construction of daily symptom scores for rhinitis, conjunctivitis, rhinoconjunctivitis, and asthma	**3**
Table S3: Scoring of rhinoconjunctivitis medication use	**4**
Table S4: Inclusion and exclusion criteria	**5**
Table S5: Description of recruitment cohorts	**7**
Clinical trial protocol	**8**
Figure S1: Trial design	**107**
The Paediatric Rhinoconjunctivitis Quality of Life Questionnaire (PRQLQ)	**108**
Definition of an event of special interest (ESI)	**109**
Statistical analysis plan	**110**
Table S6: Baseline demographics and clinical characteristics (FAS)	**161**
Table S7: Sensitivity and estimand analyses of the primary and key secondary outcomes during the primary efficacy assessment period (FAS)	**162**
Figure S2: Primary efficacy outcome (TCRS) for the SQ HDM SLIT-tablet versus placebo, by asthma and sensitisation status at baseline (FAS, observed cases)	**163**
Table S8: Key efficacy outcomes at Week 8 and Week 16 (FAS, observed cases)	**164**
Table S9: Pre-specified asthma-related outcomes during the primary efficacy assessment period (FAS, observed cases)	**165**
Figure S3: Mean change from baseline to the end-of-trial visit in immunological parameters (FAS, Canada and Poland)	**166**
Table S10: Summary of the most frequent TRAEs reported in at least 2% of subjects in the SQ HDM SLIT-tablet group (safety analysis set)	**167**
Table S11: Summary of TEAEs (safety analysis set)	**168**
Table S12: Onset and duration of the three most frequent TRAEs occurring in at least 2% of subjects in the SQ HDM SLIT-tablet group (safety analysis set)	**169**
Table S13: Summary of the most frequent TEAEs reported in at least 2% of subjects in the SQ HDM SLIT-tablet group (safety analysis set)	**170**
Narratives for subjects reporting anaphylactic/systemic allergic reactions possibly related to trial medication	**171**

Table S1: Site information

**Country**	**Number of sites that randomised subjects**
Bulgaria	10
Canada	8
France	1
Germany	5
Lithuania	5
Poland	12
Russia	17
Slovakia	4
Spain	3
Ukraine	13
United States	17
**Total**	**95**

Table S2: Construction of daily symptom scores for rhinitis, conjunctivitis, rhinoconjunctivitis, and asthma

	Rhinitis DSS	Conjunctivitis DSS	Rhinoconjunctivitis DSS	Asthma DSS
**Rhinitis symptoms**				
Runny nose	0–3	··	0–3	··
Stuffy nose	0–3	··	0–3	··
Sneezing	0–3	··	0–3	··
Itchy nose	0–3	··	0–3	··
**Conjunctivitis symptoms**				
Gritty feeling/red/itchy eyes	··	0–3	0–3	··
Watery eyes	··	0–3	0–3	··
**Asthma symptoms**				
Chest tightness	··	··	··	0–3
Wheezing	··	··	··	0–3
Cough	··	··	··	0–3
Shortness of breath	··	··	··	0–3
**Total range**	**0–12**	**0–6**	**0–18**	**0–12**

Each symptom was measured on a scale of 0 (no symptoms) to 3 (severe symptoms). DSS=daily symptom score.

Table S3: Scoring of rhinoconjunctivitis medication use

Medication	Subject dosing	Score/dose unit	Maximum daily score
**Rhinitis medication score**			
Desloratadine oral solution, 0·5 mg/ml	5 years old: 2·5 ml (1·25 mg) once daily<br>6–11 years old: 5 ml (2·5 mg) once daily<br>≥12 years old: 10 ml (5 mg) once daily	4	4
Loratadine tablets, 10 mg*	6–12 years old and >30 kg: 1 tablet (10 mg) once daily<br>>12 years old: 1 tablet (10 mg) once daily		
Mometasone furoate nasal spray, 50 µg/dose	<12 years old: 1 puff in each nostril once daily<br>≥12 years: 2 puffs in each nostril once daily	4<br>2	8
	**Maximum daily rhinitis medication score†**		**12**
**Conjunctivitis medication score**			
Desloratadine oral solution 0·5 mg/ml	5 years old: 2·5 ml (1·25 mg) once daily<br>6–11 years old: 5 ml (2·5 mg) once daily<br>≥12 years old: 10 ml (5 mg) once daily	2	2
Loratadine tablets, 10 mg*	6–12 years old and >30 kg: 1 tablet (10 mg) once daily<br>>12 years old: 1 tablet (10 mg) once daily		
Olopatadine eye drops, 1 mg/ml	1 drop in each eye twice daily	1·5	6
	**Maximum daily conjunctivitis medication score†**		**8**
	**Maximum daily rhinoconjunctivitis medication score†**		**20**

*Loratadine scored 4 points in the rhinitis score and 2 points in the conjunctivitis score, based on assumed equal efficacy of antihistamine on the four nasal symptoms and two eye symptoms. †If any subject exceeded the recommended daily dose of symptomatic medication, the maximum daily score was used.

Table S4: Inclusion and exclusion criteria

**Inclusion criteria**	
**Code**	**Criteria**
I1	Written informed consent obtained from parents/guardians before any trial-related procedures are performed[*]
I2	Male or female of any race/ethnicity and weighing 15 kg or more on the day of screening
I3	5–11 years old at randomisation
I4	A clinical history of HDM AR/C when exposed to HDM (diagnosed by a physician) of 1 year duration or more (with or without asthma) and with AR symptoms despite having received allergy pharmacotherapy during the previous year prior to the screening visit[†]
I5	Have a rhinitis DSS of ≥6, or a score of ≥5 with one symptom being severe, on ≥8 of the last 14 days of the baseline period
I6	Use symptomatic medication for the treatment of HDM AR during ≥8 of the last 14 days of the baseline period
I7	Presence of one or more of the following ARIA quality of life items due to HDM AR/C during the last 14 days of the baseline period:<br>1) sleep disturbance<br>2) impairment of daily activities, leisure and/or sport<br>3) impairment of school<br>4) troublesome symptoms
I8	Positive SPT to *D. pteronyssinus* or *D. farinae* at screening[‡]
I9	Positive *D. pteronyssinus* or *D. farinae* specific IgE (defined as ≥class 3, ≥3·5 kU/l) at screening
I10	Lung function measured by FEV_1 ≥70% of predicted value or according to local requirements while on subject's usual asthma medication following ≥6-hour washout of SABA at screening, baseline visit, and randomisation. The criteria must be fulfilled, unless subject is ≤7 years old and not able to perform reproducible FEV_1 manoeuvres despite coaching and is not considered as having a diagnosis of asthma as defined in section 11·5 of the clinical trial protocol[§]
I11	Subject willing and able to comply with trial protocol
**Exclusion criteria**	
E1	A clinically relevant history of symptomatic perennial AR/C caused by a perennial allergen source, such as animal hair and dander and/or mould, to which the subject is exposed during the baseline and/or efficacy assessment periods
E2	A clinically relevant history of symptomatic seasonal ARC and/or asthma caused by an allergen to which the subject is exposed, and which could potentially overlap with the baseline and/or efficacy assessment periods
E3	Any nasal or pharyngeal condition that could interfere with the safety or efficacy evaluation (eg, clinically relevant nasal polyps, a history of paranasal sinus surgery, or surgery of nasal turbinates).[¶] Surgery of the palatine or pharyngeal tonsils in the past is not an exclusion criterion
E4	SLIT treatment with *D. pteronyssinus* or *D. farinae* for >1 month within the last 5 years. In addition, any SLIT treatment with *D. pteronyssinus* or *D. farinae* within the previous 12 months
E5	SCIT treatment with *D. pteronyssinus* or *D. farinae* reaching the maintenance dose within the last 5 years. In addition, any SCIT treatment with *D. pteronyssinus* or *D. farinae* within the previous 12 months
E6	Ongoing treatment with any AIT product
E7	Severe chronic oral inflammation
E8	A diagnosis or history of eosinophilic oesophagitis
E9	Any immunosuppressive treatment within 3 months prior to screening
E10	Asthma requiring daily use of more than 400 µg budesonide or equivalent (see table 4 of the clinical trial protocol) at screening
E11	Any clinical deterioration of asthma that resulted in emergency treatment, hospitalisation, or treatment with systemic corticosteroids within 3 months prior to randomisation
E12	Any clinically relevant chronic disease, including malignancy that, in the opinion of the investigator, would interfere with the trial evaluations or the safety of the subject
E13	A history of chronic urticaria (>6 weeks) and/or chronic angioedema (>6 weeks) within the last 2 years prior to screening that in the opinion of the investigator may constitute an increased safety concern
E14	A relevant history of systemic allergic reaction eg, anaphylaxis with cardiorespiratory symptoms, generalised urticaria, or severe facial angioedema that, in the opinion of the investigator, may constitute an increased safety concern
E15	Active or poorly controlled autoimmune diseases, immune defects, immunodeficiencies, immunosuppression, or malignant neoplastic diseases with current disease relevance
E16	Treatment with medications with potential impact on efficacy endpoints (eg, treatment with anti-IgE drugs within 130 days/5 half-lives of the drug [whichever is longest] or treatment with antidepressant or antipsychotic medications with antihistaminergic effect)
E17	Known history of allergy, hypersensitivity or intolerance to any of the excipients or active substances of the IMP (except for *D. pteronyssinus* and/or *D. farinae*) or to any excipient of the rescue medication provided in this trial
E18	Female with positive urine pregnancy test
E19	Sexually active female of childbearing potential without medically accepted contraceptive method (appropriate methods are outlined in the clinical trial protocol)
E20	A business or personal relationship with trial staff or sponsor who is directly involved with the conduct of the trial
E21	Previously been randomised into this trial, is participating in this trial at another investigational site or is participating or planning to participate in any other clinical trial during the duration of this trial
E22	A history or current evidence of any condition, treatment, laboratory values out of range, or other circumstance that, in the opinion of the investigator, are clinically relevant and might expose the subject to risk by participating in the trial, confound the results of the trial, or interfere with the subject's participation for the full duration of the trial
E23	Has a condition or requires treatment that may increase the risk of the subject developing severe adverse reactions after adrenaline/epinephrine administration
E24	Unable to or will not comply with the use of adrenaline/epinephrine auto-injectors for countries where this is a regulatory requirement

[*]Assent from subject must be obtained according to national requirements. [†]If medical records are not available, verbal history from subject/parent/caregiver can be used to fulfil this criterion, and this must be documented in the medical records by the investigator. [‡]For subjects in North America, a positive SPT is defined as a wheal size ≥5 mm. For

subjects in Europe, a positive SPT is defined as a wheal size of ≥3 mm. If medication that could interfere with the SPT (according to table 8 of the clinical trial protocol) has not been washed out, the SPT must be repeated after the interfering medication has been washed out. [§]Subjects are considered as having a diagnosis of asthma if at least one of the following four criteria are met: 1) at least one episode of wheeze, cough, shortness of breath or chest tightness and a change in FEV_1 ≥12% after beta agonist administration; 2) recurrent wheeze with or without prolonged phase of forced exhalation observed at physical examination and an intake of asthma medication which resulted in a clinically relevant effect; 3) wheezing with or without prolonged phase of forced exhalation observed at physical examination and a change in FEV_1 ≥12% after beta agonist administration; 4) using asthma medication. [¶]If in doubt, nasal endoscopy should be performed. AIT=allergy immunotherapy. AR=allergic rhinitis. ARC=allergic rhinoconjunctivitis. AR/C=allergic rhinitis with or without rhinoconjunctivitis. ARIA=Allergic Rhinitis Impact on Asthma. *D. farinae*=*Dermatophagoides farinae*. *D. pteronyssinus*=*Dermatophagoides pteronyssinus*. DSS=daily symptom score. FEV_1=forced expiratory volume in 1 second. HDM=house dust mite. IgE= immunoglobulin type E. IMP=investigational medicinal product. SABA=short-acting beta agonist. SCIT=subcutaneous immunotherapy. SLIT=sublingual immunotherapy. SPT=skin prick test.

Table S5: Description of recruitment cohorts

The COVID-19 pandemic started on 20 March 2020, after the first subject first visit on 12 October 2019. Screening and randomisation of Cohort 1 was stopped as a consequence of the pandemic. Cohort 3 was added to recruit a sufficient number of subjects. The COVID-19 pandemic concluded on 5 May 2023, after the last contact with a subject on 21 April 2023. An overview of the timelines for the different cohorts is presented in the table below.

	Cohort 1	Cohort 2	Cohort 3
**Number of subjects randomised and treated**			
Placebo	114	313	304
SQ HDM SLIT-tablet	110	322	295
**First subject first visit**	12-Oct-2019	07-Jul-2020	07-Jul-2021
**Last subject randomised**	20-Mar-2020	01-Apr-2021	01-Apr-2022
**Last subject last visit**	On-site (V7):* 08-Jul-2021<br>Telephone (TC3):† 2-Jul-2021	On-site (V7):* 07-Apr-2022<br>Telephone (TC3):† 25-Apr-2022	On-site (V7):* 07-Apr-2023<br>Telephone (TC3):† 21-Apr-2023

*V7 refers to the end of trial. †TC3 refers to the last contact (54–59 weeks after randomisation) with the subject. HDM=house dust mite. SLIT=sublingual immunotherapy. TC=telephone call. V=visit.

Clinical trial protocol

Clinical Trial Protocol

Trial ID: MT-12

Title of Trial

A one-year placebo-controlled phase III trial evaluating the efficacy and safety of the house dust mite (HDM) SLIT-tablet in children (5-11 years of age) with HDM allergic rhinitis/rhinoconjunctivitis with or without asthma

Investigational Medicinal Product:	HDM SLIT-tablet
Phase:	Paediatric phase III
EudraCT No.:	2019-000560-22
IND No.:	015015
Sponsor:	Global Pharmacovigilance & Clinical Development<br>ALK-Abelló A/S<br>DK-2970 Hørsholm<br>Denmark<br>Phone: +45 4574 7576
Document Status:	Final
Date:	[redacted]
Version:	5.0

Confidential

Property of ALK
May not be used, divulged, published or otherwise disclosed without the written consent of ALK

Protocol synopsis

**Title of trial:**	A one-year placebo-controlled phase III trial evaluating the efficacy and safety of the house dust mite (HDM) SLIT-tablet in children (5-11 years of age) with HDM allergic rhinitis/rhinoconjunctivitis with or without asthma
**Trial ID:**	MT-12
**Development phase:**	Paediatric phase III
**Regulatory trial identifier:**	EudraCT No.: 2019-000560-22<br><br>IND No.: 015015
**Trial objectives:**	The primary objective is to demonstrate the efficacy of the HDM SLIT-tablet compared to placebo in the treatment of HDM allergic rhinitis (AR) in children (5-11 years of age) based on total combined rhinitis symptoms and medication use (TCRS) during the primary efficacy assessment period.<br><br>The key secondary objectives are to demonstrate the efficacy of the HDM SLIT-tablet compared to placebo during the primary efficacy assessment period based on:<br><br>1. Rhinitis symptoms (based on DSS)<br>2. Rhinitis medication use (based on DMS)<br>3. Combined rhinoconjunctivitis symptoms and medication use (based on TCS)<br><br>The additional secondary objectives are to evaluate the HDM SLIT-tablet compared to placebo during the primary efficacy assessment period with respect to:<br><br>• Safety and tolerability<br>• Rhinoconjunctivitis symptoms<br>• Rhinoconjunctivitis medication use<br>• Rhinoconjunctivitis quality of life (QoL)<br>• Asthma symptoms and medication use<br>• Changes in immunological parameters<br><br>The exploratory objectives are to evaluate:<br><br>• Combined rhinitis symptoms and medication use during pollen season in

	spring and summer<br>• Conjunctivitis symptoms, conjunctivitis medication use and combined conjunctivitis symptoms and medication use during the efficacy assessment period<br>• Rhinitis combined symptoms and medication use after 8 and 16 weeks of treatment<br>• Rhinoconjunctivitis combined symptoms and medication use after 8 and 16 weeks of treatment<br>• Nocturnal awakenings due to asthma requiring SABA use
**Trial endpoints:**	Primary efficacy endpoint during the efficacy assessment period is:<br>• The average daily TCRS<br>The daily TCRS is the sum of the rhinitis daily symptoms score (DSS) and the rhinitis daily medication score (DMS).<br>Key secondary efficacy endpoints during the efficacy assessment period are:<br>• The average rhinitis DSS<br>• The average rhinitis DMS<br>• The average total combined score (TCS)<br>The daily TCS is the sum of the rhinoconjunctivitis DSS (rhinitis DSS + conjunctivitis DSS) and the rhinoconjunctivitis DMS (rhinitis DMS + conjunctivitis DMS).<br>The safety and tolerability endpoints are:<br>• Treatment-emergent adverse events (TEAEs), solicited AEs, IMP-related AEs, treatment-emergent serious adverse events (SAEs), event of special interest (ESI), TEAEs leading to discontinuation, time to discontinuation due to TEAEs<br>• Vital signs, physical examination, FEV1 and clinical laboratory values during treatment and at the final visit (visit 7)<br>Additional secondary efficacy endpoints during the efficacy assessment period are:<br>• Average rhinoconjunctivitis DSS<br>• Average rhinoconjunctivitis DMS<br>• Paediatric rhinoconjunctivitis quality of life questionnaire (PRQLQ)

	• Average asthma DSS<br>• Average daily use of short-acting β_2-agonist (SABA)<br>• Rhinitis mild days<br>• Rhinitis exacerbation days (days with a rhinitis DSS of 6 or of 5 with one individual symptom scored 3 (symptom that is hard to tolerate; causes interference with activities of daily living and/or sleeping))<br>• Average rhinitis combined symptom and medication score (CSMS)<br><br>The immunologic endpoints are:<br><br>• Change from baseline in specific IgE and IgG4 to D. *pteronyssinus* and D. *farina*, change in baseline for HDM IgE-Blocking factor (IgE-BF) and change in baseline for total IgE measured at end of trial (visit 7)<br><br>The exploratory endpoints during the efficacy assessment period are:<br>• Average total combined conjunctivitis score (TCCS; the sum of the conjunctivitis DSS and the conjunctivitis DMS)<br>• Average conjunctivitis DSS<br>• Average conjunctivitis DMS<br>• Rhinitis symptom-free days<br>• Nocturnal awakenings due to asthma requiring SABA use<br><br>The exploratory endpoints derived from the 2 weeks daily eDiary collected 8 and 16 weeks after treatment initiation are:<br>• Average TCRS<br>• Average TCS<br>• Average rhinitis DSS<br>• Average rhinitis DMS<br>• TCRS during pollen season in spring and summer

**Trial design:**	This trial is an approximately one-year randomised, parallel-group, double-blind, placebo-controlled, multi-national phase III trial conducted in Europe and North America. Approximately 1370 subjects will be randomised (1:1) to receive treatment with the HDM SLIT-tablet or placebo.<br><br>The trial consists of 4 periods: screening (Period 1), a baseline period (Period 2), a treatment initiation and maintenance period (Period 3), and a primary efficacy assessment period during the last 8 weeks (period 4) of the approximately 12 months treatment.<br><br>Period 1 is the screening period. Subjects may be screened up to 12 weeks before randomisation.<br><br>Period 2 is the baseline period that lasts 21 days plus 7 days for eligibility check. Subjects' baseline must be recorded between 1 September and 1 April. In addition, pollen allergic subjects must have their baseline period outside the season of their pollen allergy. During the baseline period, subjects are allowed to take rhinoconjunctivitis and asthma rescue medication and their rhinoconjunctivitis symptoms and use of rescue medication will be rated on a daily basis in their eDiary by the parent/caregiver. To be eligible for inclusion, subjects will be required to have a rhinitis DSS of at least 6, or a score of at least 5 with one symptom being severe, on 8 of the last 14 days of the baseline period and will be required to use rescue medication for treatment of HDM allergic rhinitis during at least 8 of the last 14 days of the baseline period. The baseline period must end 1 week before randomisation to allow for eligibility check.<br><br>Period 3 is the treatment initiation and maintenance period. It begins at randomisation (visit 3) and lasts for approximately 44-49 weeks. During the first 28 days of period 3, 15 pre-specified symptoms/signs occurring after IMP intake will be recorded by the parent/caregiver together with the subject in the eDiary. If pre-specified symptoms/signs are reported in the eDiary, the investigator will evaluate the reported symptoms and report them in the eCRF as solicited AEs at visit 4.<br><br>To evaluate efficacy, there will be a 2 weeks efficacy assessment period recorded in the eDiary at week 8 and week 16.<br><br>Period 4 is the 8-week primary efficacy assessment period beginning at visit 6 (approximately 44-49 weeks after randomisation) and lasting until visit 7. Subjects' primary efficacy assessment period must be between 1 September and 1 April. The 8-week primary efficacy assessment period must be planned to include the dates that were included in the 3-week baseline period the previous year. In addition, pollen allergic subjects must have their primary efficacy assessment period outside the season of their pollen allergy. During period 4, the parent/caregiver together with the subject will rate the rhinoconjunctivitis symptoms and use of rescue medication on a daily basis in the eDiary.<br><br>A schematic of the trial design is depicted in Figure 1.

<table>
<tr><td></td><td>

Figure 1 Trial design

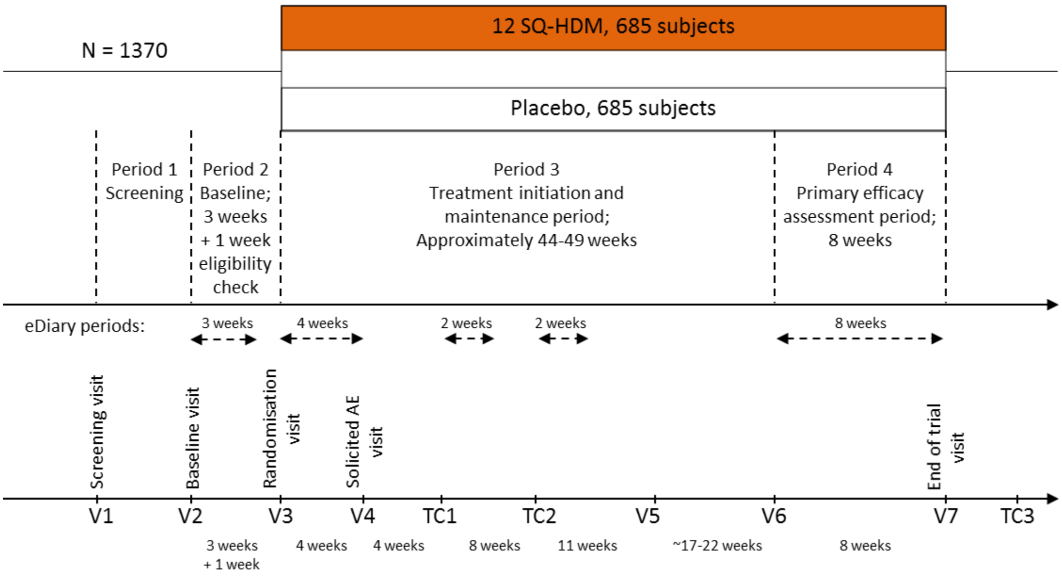

</td></tr>
<tr><td>

Trial schedule:

</td><td>

First subject first visit (FSFV): H2 Year 1

Last subject randomised: Q1 Year 4

The trial will include 3 cohorts;

- Randomisation of cohort 1: Q4 Year 1 - 01-April Year 2
- Randomisation of cohort 2: Q3 Year 2 - 01-April Year 3
- Randomisation of cohort 3: Q3 Year 3 - 01-April Year 4

Last subject last visit (LSLV): approximately 12 months after last subject randomised.

Duration of treatment per subject: approximately 12 months.

</td></tr>
<tr><td>

Trial population:

</td><td>

Subjects randomised in this trial will be children 5-11 years of age, with HDM AR with or without conjunctivitis (AR/C), and with or without asthma. Approximately 1370 subjects will be randomised (1:1) to receive treatment with the HDM SLIT-tablet or placebo.

Subjects who meet all of the inclusion criteria and none of the exclusion criteria will be considered eligible for the trial.

</td></tr>
</table>

**Inclusion criteria**	I1. Written informed consent obtained from parents/guardians before any trial related procedures are performed[1]<br>I2. Male or female of any race/ethnicity and weighing 15 kg or more on the day of screening<br>I3. 5-11 years old at randomisation<br>I4. A clinical history of AR/C when exposed to HDM (diagnosed by a physician) of 1 year duration or more (with or without asthma) and with allergic rhinitis symptoms despite having received allergy pharmacotherapy during the previous year prior to the screening visit[2]<br>I5. Have a rhinitis DSS of at least 6, or a score of at least 5 with one symptom being severe, on at least 8 of the last 14 days of the baseline period<br>I6. Use symptomatic medication for treatment of HDM allergic rhinitis during at least 8 of the last 14 days of the baseline period<br>I7. Presence of one or more of the following Allergic Rhinitis Impact on Asthma (ARIA) quality of life items due to HDM AR/C during the last 14 days of the baseline period:<br>1) Sleep disturbance<br>2) Impairment of daily activities, leisure and/or sport<br>3) Impairment of school<br>4) Troublesome symptoms<br>I8. Positive skin prick test (SPT) to *D. pteronyssinus* or *D. farinae* at screening[3]<br>I9. Positive *D. pteronyssinus* or *D. farinae* specific IgE (defined as ≥class 3, ≥3.5 kU/l) at screening<br>I10. Lung function measured by FEV1 ≥ 70% of predicted value or according to local requirements at randomisation (visit 3). For subjects having a diagnosis of asthma, FEV1 is measured while on subject's usual asthma medication following at least a 6-hour washout of SABA. The criteria must be fulfilled, unless subject is ≤7 years old and not able to perform reproducible FEV_1 manoeuvres despite coaching and is not considered as

[1] Assent from the subject must be obtained according to national requirements.
[2] If medical records are not available, verbal history from subject/parent/caregiver can be used to fulfill this criterion and this must be documented in the medical records by the investigator.
[3] A positive SPT is defined in the SPT Guideline. Briefly, for subjects in North America, a positive SPT is defined as a wheal size ≥5 mm. For subjects in Europe, a positive SPT is defined as a wheal size of ≥3 mm. If medication that could interfere with the SPT, according to Table 8, has not been washed out, the SPT must be repeated after the interfering medication has been washed out.

	having a diagnosis of asthma as defined in section 11.5 of this protocol[4].<br>I11. Subject willing and able to comply with trial protocol
**Exclusion criteria**	E1. A clinically relevant history of symptomatic perennial AR/C caused by a perennial allergen source such as animal hair and dander and/or mould to which the subject is exposed during the baseline and/or efficacy assessment periods<br>E2. A clinically relevant history of symptomatic seasonal allergic rhinoconjunctivitis and/or asthma caused by an allergen to which the subject is exposed, and which could potentially overlap with the baseline and/or efficacy assessment periods<br>E3. Any nasal or pharyngeal condition that could interfere with the safety or efficacy evaluation, (e.g., clinically relevant nasal polyps, a history of paranasal sinus surgery or surgery of nasal turbinates)[5]. Surgery of the palatine or pharyngeal tonsils in the past is not an exclusion criterion<br>E4. SLIT treatment with *D. pteronyssinus* or *D. farinae* for more than 1 month within the last 5 years. In addition, any SLIT treatment with *D. pteronyssinus* or *D. farinae* within the previous 12 months<br>E5. SCIT treatment with *D. pteronyssinus* or *D. farinae* reaching the maintenance dose within the last 5 years. In addition, any SCIT treatment with *D. pteronyssinus* or *D. farinae* within the previous 12 months<br>E6. Ongoing treatment with any allergy immunotherapy product<br>E7. Severe chronic oral inflammation<br>E8. A diagnosis or history of eosinophilic oesophagitis<br>E9. Any systemic immunosuppressive treatment, other than glucocorticosteroids, within 130 days prior to visit 1 (screening). Any oral glucocorticosteroids from 60 days prior to visit 2 (baseline). Any other systemic glucocorticosteroids (depot or parenteral) from 90 days prior to visit 2 (baseline) (see Table 2)<br>E10. Asthma requiring daily use of more than 400 mcg budesonide or equivalent (see Table 4) at screening<br>E11. Any clinical deterioration of asthma that resulted in emergency treatment, hospitalisation or treatment with systemic corticosteroids within 3 months prior to randomisation

[4] As defined in section 11.5 subjects are considered as having a diagnosis of asthma if at least one of the following 4 criteria are met:

- At least one episode of wheeze, cough, shortness of breath or chest tightness and a change in FEV_1 ≥12% after beta2-agonist administration
- Recurrent wheeze with or without prolonged phase of forced exhalation observed at physical examination and an intake of asthma medication which resulted in a clinically relevant effect
- Wheezing with or without prolonged phase of forced exhalation observed at physical examination and a change in FEV_1 ≥12% after beta2-agonist administration
- Using asthma medication

[5] If in doubt, nasal endoscopy should be performed

	E12. Any clinically relevant chronic disease, including malignancy, that in the opinion of the investigator would interfere with the trial evaluations or the safety of the subject<br>E13. A history of chronic urticaria (> 6 weeks) and/or chronic angioedema (> 6 weeks) within the last 2 years prior to screening that in the opinion of the investigator may constitute an increased safety concern<br>E14. A relevant history of systemic allergic reaction e.g. anaphylaxis with cardiorespiratory symptoms, generalised urticaria or severe facial angioedema that in the opinion of the investigator may constitute an increased safety concern<br>E15. Active or poorly controlled autoimmune diseases, immune defects, immunodeficiencies, immunosuppression or malignant neoplastic diseases with current disease relevance<br>E16. Treatment with medications with potential impact on efficacy endpoints (e.g. treatment with anti-IgE drugs within 130 days/5 half-lives of the drug (which ever longest) or treatment with antidepressant or antipsychotic medications with antihistaminergic effect)<br>E17. Known history of allergy, hypersensitivity or intolerance to any of the excipients or active substances of the IMP (except for *D. pteronyssinus* and/or *D. farinae*) or to any excipient of the rescue medication provided in this trial<br>E18. Female with positive urine pregnancy test<br>E19. Sexually active female of childbearing potential without medically accepted contraceptive method[6]<br>E20. A business or personal relationship with trial staff or sponsor who is directly involved with the conduct of the trial<br>E21. Previously been randomised into this trial, is participating in this trial at another investigational site or is participating or planning to participate in any other clinical trial during the duration of this trial<br>E22. A history or current evidence of any condition, treatment, laboratory values out of range or other circumstance that in the opinion of the investigator are clinically relevant and might expose the subject to risk by participating in the trial, confound the results of the trial, or interfere with the subject's participation for the full duration of the trial<br>E23. Has a condition or requires treatment that may increase the risk of the subject developing severe adverse reactions after adrenaline/epinephrine administration

[6] For the purpose of this protocol the following contraceptive methods are considered appropriate: oral contraceptives, trans dermal patches or depot injection of a progestogen drug (starting at least 4 weeks prior to IMP administration); double barrier method: condom or occlusive cap (diaphragm or cervical/vault caps) plus spermicidal agent; intrauterine device (IUD), intrauterine system (IUS), implant, or vaginal ring (placed at least 4 weeks prior to IMP administration); or male partner sterilisation (vasectomy with documentation of azoospermia) prior to the female subject's entry into trial and is the sole sexual partner for that female subject. Sexual abstinence is acceptable as contraceptive if it is true abstinence and in line with the usual lifestyle of the subject. Periodic abstinence (e.g., calendar, ovulation, symptothermal, post-ovulation methods) and withdrawal are not acceptable methods of contraception. However, national requirements regarding contraception should always be followed.

	E24. Unable to or will not comply with the use of adrenaline/epinephrine auto-injectors for countries where this is a regulatory requirement
**Assessments:**	The following data will be collected:<br>• Demographics<br>• Medical history (including rhinitis, conjunctivitis, asthma, atopic dermatitis, and food allergy)<br>• Concomitant and previous medication<br>• AEs<br>• Vital signs<br>• Physical examination, including oropharyngeal examinations<br>• Height and weight<br>• Lung function (FEV_1)<br>• Pregnancy test results (only applicable for female subjects of childbearing potential)<br>• Clinical safety laboratory (blood chemistry, hematology, urinalysis)<br>• SPT<br>• Serum IgE against *D. pteronyssinus* and *D. farinae*<br>Biomarker assessment:<br>• Immunological and serological parameters such as IgE, total IgE, IgG_4, and IgE-BF and other antibody isotypes against allergens as well as other serological components (only Canada and Poland)<br>• Pharmacogenetics<br>Efficacy assessments (eDiary):<br>• AR/C symptoms<br>• AR/C medication use<br>• Asthma symptoms and medication use<br>• Nocturnal awakenings due to asthma requiring SABA use<br><br>Quality of life assessments:<br>• Rhinoconjunctivitis quality of life (PRQLQ 5-11 years)<br>• Subjects' absence from school/work due to allergy and/or asthma

<table>
<tr><td></td><td>

Parents/caregivers' absence from work due to subjects' allergy and/or asthma
Health care utilisation (visits to hospitals, emergency rooms and doctors) due to subjects' allergy and/or asthma

</td></tr>
<tr><td>Trial medication</td><td>
<table>
<tr><th>IMP:</th><th></th></tr>
<tr><td>Active ingredients:</td><td>Standardised allergen extract from the HDMs D. pteronyssinus and D. farinae</td></tr>
<tr><td>Dosage form:</td><td>Oral lyophilisate</td></tr>
<tr><td>Dose/Strength</td><td>12 SQ-HDM</td></tr>
<tr><td>Excipients:</td><td>Gelatine (fish source), mannitol and sodium hydroxide</td></tr>
</table>
<table>
<tr><th>Placebo:</th><th></th></tr>
<tr><td>Active ingredients:</td><td>None</td></tr>
<tr><td>Dosage form:</td><td>Oral lyophilisate</td></tr>
<tr><td>Dose/Strength</td><td>Placebo</td></tr>
<tr><td>Excipients:</td><td>Gelatine (fish source), mannitol and sodium hydroxide</td></tr>
</table>
Rescue medication:
Rescue medication must be used according to the product's labelling (e.g. SmPC or USPI).

Rhinoconjunctivitis:
<ol type="a">
Loratadine tablets, 10 mg or
Desloratadine, oral solution 0.5 mg/ml
Olopatadine eye drops, 1 mg/ml
Mometasone furoate nasal spray, 50 microgram/dose

Asthma:
<ol type="a">
SABA (i.e., albuterol/salbutamol inhaler)

Severe allergic reaction for countries where this is a regulatory requirement:
<ol type="a">
Adrenaline/epinephrine auto-injector

</td></tr>
<tr><td></td><td>The use of leukotriene receptor antagonists (LTRA) is permitted as concomitant medication for continued use on same dose only but will not be provided.</td></tr>
</table>

Table of contents

Protocol synopsis 2
Table of contents 12
Table of figures 15
Table of tables 15
List of appendices 16
Table of abbreviations 16
Table of definitions 19
Table of revisions 20
Table 1 - Flow chart 21
1 Introduction 24
1.1 Disease background and current treatment modalities 24
1.2 Stage of development 24
1.3 Trial rationale 26
1.4 Benefit-risk assessments and ethical considerations 26
2 Objectives and endpoints 29
2.1 Primary objective 29
2.2 Key secondary objectives 29
2.3 Secondary objectives 29
2.4 Explorative objectives 29
2.5 Primary efficacy endpoint 30
2.6 Key secondary efficacy endpoints 30
2.7 Additional secondary efficacy endpoints 30
2.8 Exploratory endpoints 31
3 Trial design 31
3.1 Summary of trial design 31
3.2 Trial schedule 33
3.3 Discussion of design 33
4 Trial population 35
4.1 Inclusion criteria 35
4.2 Exclusion criteria 36
5 Subject discontinuation 38
5.1 Discontinuation from IMP treatment 38
5.2 Discontinuation from trial 38
6 Randomisation and treatment blinding/unblinding 39

6.1 Subject ID number ... 39
6.2 Randomisation ... 39
6.3 Subject card ... 39
6.4 Treatment blinding/unblinding ... 40
7 Restricted and prohibited concomitant medication ... 40
8 Trial products ... 43
8.1 IMP ... 43
8.2 Rescue medication ... 44
8.3 Packaging and labelling ... 46
8.4 Handling and storage ... 46
8.5 IMP and rescue medication accountability ... 46
8.6 Reporting of technical complaints ... 47
9 Treatment ... 47
9.1 Posology and method of administration ... 47
9.2 Precautions in relation to first dosing ... 47
9.3 Rescue medication for severe allergic reactions ... 48
9.4 Temporary interruption and discontinuation of treatment ... 48
9.5 IMP compliance ... 48
9.6 Post-trial treatment ... 49
10 Visit schedule ... 50
11 Assessments ... 56
11.1 Informed consent ... 57
11.2 Consent for long-term storage of biobank samples for Canada and Poland ... 57
11.3 Consent for collection of blood samples for pharmacogenetic biobank ... 58
11.4 Demographics ... 58
11.5 Medical history ... 58
11.6 Concomitant and previous medication ... 59
11.7 Height and weight ... 59
11.8 Vital signs ... 59
11.9 Lung function ... 59
11.10 Physical examination ... 59
11.11 Pregnancy test ... 60
11.12 Skin prick test ... 60
11.13 Blood and urine sampling ... 62
11.14 Laboratory assessments ... 63

11.15 Adverse event assessment ... 65
11.16 Assessment of rhinoconjunctivitis and asthma symptoms ... 66
11.17 Medication assessment. ... 67
11.18 Local and systemic allergic reaction emergency plan ... 69
11.19 Solicited AEs ... 69
11.20 Eosinophilic oesophagitis ... 70
11.21 Subject reported outcomes ... 71
11.21.1 eDiary assessments ... 71
11.21.2 Paediatric quality of life questionnaire ... 71
12 Adverse events ... 71
12.1 Definitions ... 72
12.2 Assessments ... 73
12.3 Collection, recording and reporting of adverse events ... 74
12.4 Data monitoring committee ... 77
13 Early termination of trial ... 77
14 Data handling ... 78
14.1 eCRF ... 78
14.2 eDiary ... 78
14.3 Query handling ... 79
14.4 Laboratory data ... 79
14.5 Database lock ... 79
15 Statistical methods ... 80
15.1 Sample size and power considerations ... 80
15.2 Analysis data sets ... 82
15.3 Subject disposition ... 82
15.4 Demographic and baseline characteristics ... 82
15.5 Extent of exposure ... 82
15.6 Previous and concomitant therapy ... 82
15.7 Efficacy analyses ... 83
15.8 Safety analyses ... 84
15.9 Multiplicity ... 84
15.10 Interim analyses ... 84
15.11 Handling of missing data ... 85
16 Quality assurance and control ... 85
16.1 Monitoring ... 85

16.2 Source data and access to source documents 85
16.3 Investigator site file – and other trial documentation 87
16.4 Protocol compliance 87
16.5 Audit 87
17 Ethics and regulatory procedures 88
17.1 Statement of compliance 88
17.2 Disclosure and confidentiality 88
17.3 Subject confidentiality 88
17.4 Data protection 89
17.5 IEC/IRB/regulatory authorities 90
17.6 Inspections 90
17.7 Protocol amendment and other changes in trial conduct 90
18 Reporting and publication 91
18.1 Integrated clinical trial report 91
18.2 Publication of results 91
19 Finance and insurance 92
20 Trial organisation 92
21 Reference list 93

Table of figures

Figure 1 Trial design 6
Figure 2 Trial design 32

Table of tables

Table 1 Flow chart 21
Table 2 Restricted and prohibited concomitant medications 40
Table 3 Schedule for rhinitis and conjunctivitis rescue medication 44
Table 4 Definition of low, medium and high dose ICS for subjects aged 5-11 years 45
Table 5 Visit schedule 50
Table 6 Physical examination 60
Table 7 Skin prick test 61
Table 8 Medication with a possible interference with SPT 62
Table 9 Subject's symptom scoring 66
Table 10 Construction of symptom scores[1] 67
Table 11 Scoring of rhinoconjunctivitis rescue medication 68
Table 12 Scoring of rhinoconjunctivitis medication for the CSMS (Pfaar et al. 2014) 69
Table 13 Mean and standard deviation of TCRS by treatment group in MT-06 81
Table 14 Effects size of TCRS in MT-06, P001 and PO-203-3-3 81
Table 15 Power calculations based on different assumptions of the effect size 81

List of appendices

Appendix 1: Rationale(s) for amendment(s)

Appendix 2: Investigator agreement on clinical trial protocol

Appendix 3: Signature page

Appendix 4: Trial organisation

Appendix 5: Patient reported outcome questionnaire

Appendix 6: COVID-19 Pandemic Documents

Table of abbreviations

AA	Allergic asthma
AE	Adverse event
AIT	Allergy immunotherapy
ALK	ALK-Abelló A/S
ALT	Alanine aminotransferase
AMP	Auxiliary medicinal product
AR	Allergic rhinitis
AR/C	Allergic rhinitis/rhinoconjunctivitis
ARIA	Allergic Rhinitis Impact on Asthma
ASaT	All-subjects-as-treated analysis set
ATC	Anatomical Therapeutical Chemical classification system
ATS	American Thoracic Society
BP	Blood pressure
CBER	Center for Biologics Evaluation and Research
CHMP	The Committee for Medicinal Products for Human Use
CRA	Clinical research associate
CRF	Case report form
CSMS	Combined symptom and medication score (recommended by European Academy of Allergy and Clinical Immunology task force (Pfaar et al. 2014))
COVID-19	Coronavirus disease 2019
D. farinae	*Dermatophagoides farinae*
D. pteronyssinus	*Dermatophagoides pteronyssinus*
DMC	Data monitoring committee
DMS	Daily medication score
DNA	Deoxyribonucleic acid
DSS	Daily symptom score
eCRF	Electronic case report form
eDiary	Electronic diary
EMA	European Medicines Agency

EMEA	European Medicines Agency (previous abbreviation)
ERS	European Respiratory Society
ESI	Event of special interest
EU	European Union
EudraCT	European Union drug regulating authorities clinical trials database
FAS	Full analysis set
FDA	Food and Drug Administration
FEV_1	Forced expiratory volume in 1 second
FSFV	First subject first visit
FVC	Forced vital capacity
GCP	Good clinical practice
GDPR	General Data Protection Regulation
GINA	Global initiative for asthma
GLMM	Generalised linear mixed model
HDM	House dust mite
IB	Investigator’s brochure
ICH	International council on harmonisation of technical requirements for registration of pharmaceuticals for human use
ICS	Inhaled corticosteroid
ICTR	Integrated clinical trial report
IEC	Independent ethics committee
IgE	Immunoglobulin E
IgE-BF	Immunoglobulin E blocking factor
IgG4	Immunoglobulin G4
IMP	Investigational medicinal product
IRB	Institutional review board
IRT	Interactive response technology
LABA	Long-acting β_2-agonist
LME	Linear mixed effect
LSLV	Last subject last visit
LTRA	Leukotriene receptor antagonist
MedDRA	Medical dictionary for regulatory activities
MI	Multiple imputation
MMRM	Mixed-effect model for repeated measurement
PDCO	Paediatric committee
PEF	Peak expiratory flow
PRQLQ	Paediatric rhinoconjunctivitis quality of life questionnaire
QoL	Quality of life
RNA	Ribonucleic acid
SABA	Short-acting β_2-agonist
SAE	Serious adverse event
SAF	Safety analysis set
SAP	Statistical analysis plan

SCIT	Subcutaneous immunotherapy
CDISC SDTM	Clinical data interchange standards consortium study data tabulation model
SLIT	Sublingual immunotherapy
SmPC	Summary of product characteristics
SOP	Standard operating procedure
SPT	Skin prick test
SSR	Sample size reassessment
SUSAR	Suspected unexpected serious adverse reaction
TC	Telephone call
TCCS	Total combined conjunctivitis score
TC-FU	Follow-up telephone call
TCRS	Total combined rhinitis score
TCS	Total combined score (of rhinoconjunctivitis symptoms and medication)
TEAE	Treatment-emergent adverse event
TNSS	Total nasal symptom score
USPI	United States prescribing information
UV	Unscheduled visit
WAO	World Allergy Organization
WHO	World Health Organization

Table of definitions

AE	An AE is any untoward medical occurrence in a clinical trial subject and which does not necessarily have a causal relationship with the administered IMP
Completed subject	A randomised subject is considered as completed if he/she has not discontinued the trial
Concomitant medication	All medications being continued by a subject on entry into the trial and all medications given in addition to IMP and rescue medication
End of trial	The end of trial is defined as the date of the last subject last visit
IMP	A pharmaceutical form of the active substance or placebo being tested
Solicited AE	A pre-specified AE recorded by the investigator based on the symptoms reported in the subject's eDiary during the first 28 days after randomisation
Source documents	Source documents are original documents, data, and records from which the subjects' eCRF data are obtained. These include, but are not limited to, hospital records (from which medical history and concomitant medication may be summarised into the eCRF), clinical and office charts, laboratory and pharmacy records, diaries, microfiches, radiographs, and correspondence
Trial completion	The trial is completed once the ICTR is signed
Treatment-emergent AE	An AE that starts on or after the first IMP administration and no later than 7 days after the last IMP administration

Table of revisions

Date	Version	Description of document
08-Mar-2019	1.0	Final Protocol
15-Mar-2019	2.0	Final Protocol (including Protocol Amendment no. 1)
21 May 2019	3.0	Final Protocol (including Protocol Amendment no. 2)<br>Method of contraception added<br>Exploratory endpoint (daily SABA use) changed to secondary endpoint<br>Few administrative/typos changed
13 Dec 2019	4.0	Final Protocol (including Protocol Amendment no. 3)<br>Documentation of HDM AR/C diagnosis<br>Spirometry reference<br>Spirometry in subjects ≤7 years and without asthma (inclusion criteria 10)<br>GDPR<br>Several administrative/typos changed
19 Mar 2021	5.0	Final Protocol (including Protocol Amendment no. 4)<br><br><u>Changes due to the COVID-19 pandemic:</u><br>• For inclusion criterion 10 (I10), lung function assessment has been removed from visit 1 (screening) and visit 2 (baseline). Lung function assessment has also been removed from visit 4, visit 5, and visit 6<br>• The observation period at the clinic after the first IMP intake (visit 3) has been reduced from 60 minutes to 30 minutes. Due to this change, the oropharyngeal examination has also been changed from 60 minutes to 30 minutes after IMP intake<br>• Section 1.4 has been updated with considerations concerning the COVID-19 pandemic<br>• Appendix 6 has been added to provide an overview of documents concerning the COVID-19 pandemic<br><br><u>Other changes:</u><br>• The oropharyngeal examination window has been extended with ± 5 minutes<br>• Clarifying wordings have been added regarding; use of immunosuppressive treatment, oral tablet (glucocorticosteroids), loratadine dispensation and for which subjects rescue medication for asthma (SABA) can be dispensed<br>• LABA has been removed from the prohibited medication list (Table 2)<br>• Several administrative changes and typos

Table 1 - Flow chart

Table 1 Flow chart[7]

**Visit ID:**	**V1**	**V2**	**V3**	**V4**	**TC1**	**TC2**	**V5**	**V6**	**V7**	**TC 3**	**UV**
Visit	Screening	Base-line	Randomi-sation	Solicited AEs					End of trial	Follow-up	Unschedul-ed visit
Time from randomisation (IMP initiation)	Max -12 weeks	-4 weeks -7 d		4 weeks + 7 d	8 weeks ± 7 d	16 weeks ± 7 d	27 weeks ± 7 d	44-49 weeks ± 7 d	52-57 weeks ± 7 d[8]	+ 2 weeks from V7 + 7 d	
Informed consent	X										
Demography	X										
Medical history	X										
Assess symptoms of eosinophilic oesophagitis	X	X	X	X	X	X	X	X	X	X	(X)
Record previous and concomitant medication	X	X	X	X	X	X	X	X	X	X	X
Physical examination	X								X		(X)
Oropharyngeal examination		X	X[9]	X			X	X			(X)
Height and weight[10]	X	X	X	X			X	X	X		X
Vital signs	X	X	X	X			X	X	X		(X)
Body temperature			X								
FEV_1			X						X		(X)
Urine pregnancy test, if applicable[11]	X	X	X	X			X	X	X		(X)
SPT	X	(X)[12]									
In-/exclusion criteria	X	X	X								
Blood and urine samples for safety laboratory assessments	X								X		(X)
Blood sample for specific IgE[13]	X[14]										(X)

[7] To the extent possible, all examinations scheduled for the final visit must be performed on subjects who receive IMP but do not complete the trial according to the protocol. If possible, the TC follow-up (TC 3) should be performed and the corresponding eCRF pages should be filled in

[8] V7 to be performed 8 weeks +14 days after V6 and no later than 1st April

[9] Oropharyngeal examinations will be done before and 30 ±5 mins after IMP administration at Visit 3, see Table 5

[10] If applicable, adjust the local and systemic allergic reaction emergency plan

[11] For female subjects of childbearing potential

[12] SPT to be performed at visit 2, if not possible at visit 1, due to necessary wash-out of concomitant medication

[13] IgE against *D pteronyssinus and D farinae*

[14] Inform subjects that continued participation in trial depends on the result of the blood sample for specific IgE against *D. pteronyssinus* and *D. farinae*

**Visit ID:**	**V1**	**V2**	**V3**	**V4**	**TC1**	**TC2**	**V5**	**V6**	**V7**	**TC 3**	**UV**
Assess and record AEs in eCRF	X	X	X	X	X	X	X	X	X	X	X
Randomisation			X								
Issue and review local and systemic allergic reaction emergency plan			X								
PRQLQ			X				X	X	X		
Record absence from school/work for subject and parent/caregiver			X	X	X	X	X	X	X		(X)
Record health care utilisation			X	X	X	X	X	X	X		(X)
Blood sample for pharmacogenetics biobank[15]									X		
Intake of IMP at clinic			X								(X)
Dispense IMP			X	X			X	X			(X)
Dispense rhinoconjunctivitis and asthma rescue medication and instruct in the use		X	X	X			X	X			(X)
Dispense adrenaline/ epinephrine auto-injectors[16]			X	X			X	X			(X)
Collect unused adrenaline/ epinephrine auto-injector									X		
Collect rescue medication as applicable and perform drug accountability			X	X			X	X	X		
Collect IMP, perform drug accountability and IMP compliance check				X			X	X	X		
Show and discuss trial video		X						X			
Issue and instruct parent/caregiver in use and activation of eDiary		X	X		X	X		X			X

[15] For subjects where the subject/parent/guardian has given consent
[16] For countries where this is a regulatory requirement

Visit ID:	V1	V2	V3	V4	TC1	TC2	V5	V6	V7	TC 3	UV
Instruct in the recording of pre-specified symptoms in eDiary			X								
Review eDiary and record solicited AEs in eCRF				X							
eDiary recording		3 weeks	-4 weeks		2 weeks	2 weeks		8 weeks			
Check eDiary compliance			X	X					X		
Collect eDiary									X		

For Canada and Poland only:

Visit ID:	V1	V2	V3	V4	TC1	TC2	V5	V6	V7	TC 3	UV
Blood sample for immunological assessments	X								X		
Biobank blood sample[17]	X								X		

[17] For subjects where the subject/parent/guardian has given consent

1 Introduction

1.1 Disease background and current treatment modalities

HDM allergy is an IgE-mediated hypersensitivity reaction to allergens contained in HDM body fragments and faeces. These particles are airborne and therefore inhaled reaching the human immune system primarily through the mucosal membrane of the airways, causing perennial rhinitis and/or asthma **(Arlian & Platts-Mills 2001)**. In Europe and North America, patients typically have symptoms all year round but with an increase in symptoms during the winter months **(Bousquet et al. 2001)**.

HDM is a prominent cause of inhalant allergy in Europe and North America, with a prevalence of IgE sensitisation as high as 49% **(Arbes, Jr. et al. 2005; Bauchau & Durham 2004)**. HDM is also an important allergen among children and adolescents, with HDM sensitisation rates in inner city metropolitan areas in the US showing up to 62% of all inner city children sensitised to HDM **(Gruchalla et al. 2005)**. Allergy symptoms can have a significant negative impact on quality of life. A survey of children and adolescents 4 to 17 years of age and their parents or caregivers indicated that allergies decreased school performance, interfered with daily activities, and disrupted sleep patterns **(Meltzer et al. 2009)**.

Currently, treatment of allergic diseases is largely based on allergen avoidance, pharmacotherapy and AIT **(Bacharier et al. 2008; Bousquet et al. 2008; Bousquet et al. 1998; GINA Executive Committee 2017; van Cauwenberge et al. 2000)**. However, evidence shows that allergen avoidance is not possible to an extent that relieves patients from their symptoms **(Gotzsche & Johansen 2008; Valovirta et al. 2008)**, and international treatment guidelines question whether the effect justifies the cost and effort **(Brozek et al. 2010; GINA Executive Committee 2017)**.

HDM AR/C can be treated with pharmacotherapy such as oral/ocular antihistamines or nasal steroids; however, a large proportion of the sufferers are inadequately controlled by these medications **(Calderon et al. 2015)**. AIT modifies the underlying immunological mechanisms responsible for allergic inflammation and represents a treatment option for HDM allergy that is complementary to pharmacotherapy. AIT is performed by repeated subcutaneous or sublingual administration of specific allergen to an allergic person in order to gradually induce immunological tolerance towards the allergen. Considerations for initiating AIT include disease severity, lack of efficacy of pharmacotherapy, side effects of pharmacotherapy, patient preference and the presence of more than one manifestation of the underlying allergic disease, and for children also the potential to modify the underlying progression of allergic disease **(Bousquet et al. 2008; Jacobsen et al. 2007; Novembre et al. 2004)**.

1.2 Stage of development

The HDM SLIT-tablet is formulated as a fast-dissolving pharmaceutical formulation (oral lyophilisate) containing standardised HDM allergen extract. Treatment involves once daily sublingual administration to provide clinical benefit in both HDM AR and HDM AA.

The HDM SLIT-tablet has obtained marketing authorisation in 14 European countries, the United States, Canada, Japan, Australia, New Zealand, South Korea, Malaysia, Philippines, Hong Kong, and Thailand.

The HDM SLIT-tablet is indicated for the treatment of HDM AR in adults in all countries mentioned above, and for the treatment of HDM AR in adolescents (12-17 years of age) in Europe, Japan, Australia, South Korea, Malaysia, Philippines, and Thailand. In addition, the 6 SQ-HDM dose is indicated for the treatment of HDM AR in young children (<12 years of age) in Japan. Finally, the HDM SLIT-tablet is indicated for the treatment of HDM AA in adults in Europe, Australia, New Zealand, South Korea, Malaysia, Philippines, and Thailand.

To date, the HDM SLIT-tablet has been investigated in 15 randomised, double-blind, placebo-controlled trials worldwide. This includes 2 paediatric phase I trials with doses up to 12 SQ-HDM (1 in children 5-14 years of age; 1 in adolescents 12-17 years of age) and 1 paediatric phase III trial with the 6 SQ-HDM dose in children and adolescents 5-17 years of age. In addition, adolescent subjects were included in 3 adult phase III trials.

A total of 1245 paediatric subjects (<18 years) have been included in the completed clinical trials, 271 of whom were treated with the 12 SQ-HDM dose. The majority of paediatric subjects exposed to 12 SQ-HDM (n=265; 98%) were adolescents.

The efficacy and safety of treatment with the HDM SLIT-tablet in HDM AR have been investigated in 3 very similar phase III trials in adults/adolescents and in 1 paediatric phase III trial, as described in the following. In addition, 1 paediatric asthma trial is currently ongoing.

The 3 phase III trials MT-06 **(Demoly et al. 2016)**, P001 **(Nolte et al. 2016)**, and TO-203-3-2 **(Okubo et al. 2016)** were conducted in Europe, North America, and Japan to demonstrate efficacy in adults with HDM AR. Adolescent subjects were included in the P001 and TO-203-3-2 trials. The 3 trials had a very similar design; all 3 were randomised, parallel-group, double-blind, placebo-controlled trials assessing primary efficacy based on average TCRS during the last 8 weeks of approximately 1 year of daily treatment. Across trials, a consistent treatment benefit was demonstrated, with statistically significantly lower TCRS reported by subjects on active treatment compared to placebo. Absolute TCRS differences from placebo were approximately 1 in all trials, corresponding to relative effect sizes between 17% and 22%. Similar numbers were seen for adolescent subpopulations. The HDM SLIT-tablet (6 and 12 SQ-HDM doses) was generally well tolerated with a safety profile characterised by frequent but transient local allergic events (e.g. oral pruritus, throat irritation, mouth oedema and oral paraesthesia) that were typically assessed as mild or moderate in intensity. Severe allergic reactions were uncommon. No treatment-related SAEs were reported, one SAE of anaphylactic reaction occurred in a subject on placebo in the TO-203-3-2 trial. Stratified safety analyses showed no differences in the safety profile between subjects <18 years of age and subjects ≥18 years of age. Across the adolescent subpopulation, no severe local swellings were reported and no adolescent subjects were treated with adrenaline due to AEs. A single event of anaphylactic reaction in an adolescent subject was reported: a subject in the 12 SQ-HDM group experienced moderate throat swelling after consuming a cookie; the event occurred 2 days after IMP discontinuation and was assessed as unrelated to treatment **(Matsuoka et al. 2017)**.

The paediatric phase III trial TO-203-3-3 was a randomised, parallel-group, double-blind, placebo-controlled trial conducted in Japan to investigate the efficacy and safety of the HDM SLIT-tablet (dose 6 SQ-HDM) in children and adolescents (5 to 17 years) with moderate-to-severe HDM AR **(Masuyama et al. 2018)**. 458 subjects were randomised to once daily treatment with 6 SQ-HDM or placebo for 1 year. The primary endpoint was the average TCRS during the last 8 weeks of treatment. The primary efficacy analysis showed that TCRS was statistically significantly lower in the 6 SQ-HDM group compared to placebo. The absolute

TCRS difference from placebo was 1.22 (p<0.0001), corresponding to a relative difference of 23%. The HDM SLIT-tablet (dose 6 SQ-HDM) was generally well tolerated. Most treatment-related AEs were mild or moderate in intensity and included predominantly the local application site AEs. No treatment-related SAEs were reported.

In addition, a paediatric asthma trial (MT-11) investigating the efficacy and safety of the HDM SLIT-tablet in children and adolescents (5-17 years of age) with HDM allergic asthma is currently ongoing. MT-11 is a phase III, double-blind, parallel-group, placebo-controlled trial conducted in Europe and North America. The trial will include 600 paediatric subjects with HDM allergic asthma and a history of frequent exacerbations, who will be randomised to once daily treatment with 12 SQ-HDM or placebo for an intended treatment period of approximately 2 years. The primary endpoint is the rate of protocol-defined clinically relevant asthma exacerbations assessed after at least 4 months of treatment. Recruitment was inititated in 2018. While the trial is ongoing and therefore still blinded, there have not been any unexpected safety findings.

Please refer to the current IB for more details.

1.3 Trial rationale

Currently, there is no approved SLIT product in Europe or North America for the treatment of HDM allergy in children (5 to 11 years old) with a history of AR/C symptoms when exposed to house dust mites.

The key purpose of this trial is to investigate whether treatment with the HDM SLIT-tablet has an acceptable safety profile and will provide a relevant treatment benefit by improving AR symptoms and reduce use of AR pharmacotherapy in children with HDM AR/C with or without asthma.

Subjects will be randomised (1:1) to treatment with the HDM SLIT-tablet (12 SQ-HDM) or placebo for a treatment period of approximately 1 year. The primary efficacy objective will be assessed based on the combined rhinitis symptom and medication score during the 8-week efficacy assessment period.

The 12 SQ-HDM dose of the HDM SLIT-tablet was selected based on data from the paediatric phase I trial and the subsequent phase III trials including adolescents **(Corzo et al. 2014; Matsuoka et al. 2017)**, and based on the fact that 12 SQ-HDM is the approved dose in adults in Europe and North America. Using the same dose for adults and children is in line with clinical practice in AIT, as supported by treatment guidelines stating that the immune response in children and adolescents is no different from that of adults **(Cox et al. 2011; EMEA 2008)**.

The design of the trial complies with the FDA recommendations and the EMA guideline for development of products for specific immunotherapy for treatment of allergic diseases **(EMEA 2008; FDA 2018)**.

1.4 Benefit-risk assessments and ethical considerations

As described in section 1.1, AR/C is a disease with considerable impact on the quality of life of patients, not only in adults but also in children and adolescents **(Meltzer et al. 2009;**

Terreehorst et al. 2002). AR/C symptoms may compromise quality of sleep leading to daytime fatigue, and AR/C has been found to result in missed school days as well as decreased performance when in the classroom. In addition, AR/C is an independent risk factor in co-morbid diseases such as asthma, sinusitis, eczema, eustachian tube dysfunction, and otitis media (Mir et al. 2012; Nathan 2007).

Among inhalant allergens, HDM sensitisation was found to be the most important risk factor for asthma development in children (Arshad et al. 2001; Lodge et al. 2011; Sears et al. 1989; Sporik et al. 1990; Terreehorst et al. 2002).

While HDM AR/C can be treated with pharmacotherapy, such as oral antihistamines or nasal steroids, many patients remain inadequately controlled by these medications (Canonica 2007). As such, there is an unmet medical need for improved treatment options for paediatric patients with HDM AR/C.

Treatment with the HDM SLIT-tablet targets the underlying HDM allergy to provide clinical benefit in both HDM AR/C and HDM AA. Thus, in the present trial, subjects in the active treatment group may experience treatment benefits in terms of a reduced burden of symptoms related to HDM AR/C as well as HDM AA.

The HDM SLIT-tablet is an orolyophilisate to be placed under the tongue. The sublingual route provides an advantage over subcutaneous injection, as it has a safety profile allowing for at-home administration. This results in improved convenience for patients and caters to those who have a fear of injections or who cannot set aside time off work or school for frequent doctor's visits over an extended treatment period. In addition, orodispersible dosage forms hold great promise for children as they are easy to administer and, as long as dispersion is rapid, are difficult to spit out or aspirate (EMEA 2006).

The most prevalent AEs observed with the HDM SLIT-tablet include local reactions in the mouth and throat (e.g. oral pruritus, throat irritation, and mouth oedema) of mild or moderate severity. There is a risk of more significant adverse events, such as systemic allergic reactions or larger swellings in the oral cavity. Across the adolescent subpopulation included in the HDM SLIT-tablet development programme, no severe local swellings have been reported and no adolescent subjects were treated with adrenaline due to AEs. A single anaphylactic reaction in an adolescent subject in the 12 SQ-HDM group was reported; the event occurred 2 days after IMP discontinuation and was assessed as unrelated to treatment (Matsuoka et al. 2017).

While the risk of systemic allergic reactions applies to any AIT, the risk of severe systemic allergic reactions is considered minimised for the HDM SLIT-tablet, as sublingual administration has not been found to result in relevant absorption into the systemic circulation. The risk of severe swellings in the oral cavity is low, and there have been no local allergic swelling that compromised the airways in completed clinical trials. Cases of systemic allergic reactions, including anaphylactic reactions have been reported post marketing and are considered a class effect. The medical supervision at first IMP intake is therefore an important precaution. In some cases, the serious systemic allergic reaction has occurred at doses subsequent to the initial dose. In the present trial, all subjects/parents/caregivers will be provided with educational information regarding symptoms of local and systemic allergic reactions and treatment, including a written local and systemic allergic reaction emergency plan.

This clinical trial will follow the principles of the Helsinki Declaration 1964 and subsequent amendments and clarifications (**World Medical Association 2013**). The trial will be approved by the local IEC/IRB and regulatory authorities before initiation.

A placebo group will be used as a control group in the trial. A placebo group is considered ethically justifiable since subjects in both treatment groups will be provided with rescue medication to treat respiratory allergy symptoms during the trial. Furthermore, subjects will be medically monitored during the trial and provided with appropriate treatment, if warranted. Finally, subjects can discontinue from the trial at any time without penalty or loss of benefits to which the subject was otherwise entitled, if they find the treatment intolerable and without giving a reason.

The population selected for this trial includes children with symptomatic AR/C when exposed to HDM. In order to minimise the burden of the disease for subjects enrolled in the trial, and to minimise the risk related to trial activities, the following measures have been included in the trial protocol:

- Each subject will receive a supply of rhinoconjunctivitis and asthma rescue medication to be used as needed. In countries where it is a regulatory requirement, adrenaline/epinephrine auto-injectors will be dispensed
- Each subject will be provided with a pocket-size patient card containing investigator contact information
- An independent DMC will be established for safety monitoring of the trial
- Regarding COVID-19 pandemic, it is important to highlight that subjects' safety is always first priority. Participating in this trial, including receiving treatment with AIT, is not expected to increase subjects' risk of contracting communicable diseases, including COVID-19. Appendix 6 gives an overview of documents covering:
    - COVID-19 pandemic measure and local requirements
    - Conduct of remote visit if an on-site visit is not possible due to COVID-19 pandemic
    - Shipment of IMP and rescue medication directly to patient if an on-site visit is not possible due to COVID-19 pandemic

In summary, the benefits of treatment with the HDM SLIT-tablet, which are expected to include reduced rhinitis symptoms and a reduced need for rescue medication, are deemed to outweigh the risks and inconveniences associated with the treatment. The safety profile of the HDM SLIT-tablet in the paediatric population is expected to be favourable with a low risk of systemic adverse reactions. The most frequent adverse reactions are expected to be mild or moderate local reactions in the oral cavity, which makes the HDM SLIT-tablet suitable for at-home administration. Additional details regarding specific benefits and risks for subjects participating in this clinical trial can be found in the accompanying IB and informed consent documents.

In conclusion, the risks and benefits of trial participation are considered to be well balanced.

2 Objectives and endpoints

2.1 Primary objective

The primary objective is to demonstrate the efficacy of the HDM SLIT-tablet compared to placebo in the treatment of HDM allergic rhinitis (AR) in children (5-11 years of age) based on total combined rhinitis symptoms and medication use (TCRS) during the primary efficacy assessment period.

2.2 Key secondary objectives

The key secondary objectives are to demonstrate the efficacy of the HDM SLIT-tablet compared to placebo during the primary efficacy assessment period based on:

1. Rhinitis symptoms (based on DSS)
2. Rhinitis medication use (based on DMS)
3. Combined rhinoconjunctivitis symptoms and medication use (based on TCS)

2.3 Secondary objectives

The additional secondary objectives are to evaluate the HDM SLIT-tablet compared to placebo during the primary efficacy assessment period based on:

- Safety and tolerability
- Rhinoconjunctivitis symptoms
- Rhinoconjunctivitis medication use
- Rhinoconjunctivitis QoL
- Asthma symptoms and medication use
- Changes in immunological parameters

2.4 Explorative objectives

The explorative objectives are to evaluate:

- Combined rhinitis symptoms and medication use during pollen season in spring and summer
- Conjunctivitis symptoms, conjunctivitis medication use and combined conjunctivitis symptoms and medication use during the efficacy assessment period
- Rhinitis combined symptoms and medication use after 8 and 16 weeks of treatment
- Rhinoconjunctivitis combined symptoms and medication use after 8 and 16 weeks of treatment
- Nocturnal awakenings due to asthma requiring SABA use

2.5 Primary efficacy endpoint

The primary efficacy endpoint during the efficacy assessment period is:

- The average daily TCRS

The daily TCRS is the sum of the rhinitis DSS, and the rhinitis DMS.

2.6 Key secondary efficacy endpoints

The key secondary efficacy endpoints during the efficacy assessment period are:

- The average rhinitis DSS
- The average rhinitis DMS
- The average daily TCS

The daily TCS is the sum of the rhinoconjunctivitis DSS (rhinitis DSS + conjunctivitis DSS) and the rhinoconjunctivitis DMS (rhinitis DMS + conjunctivitis DMS).

2.7 Additional secondary efficacy endpoints

The safety and tolerability endpoints are:

- TEAEs, solicited AEs, ESI, IMP-related AEs, treatment-emergent SAEs, TEAEs leading to discontinuation, and time to discontinuation due to TEAEs
- Vital signs, physical examination, FEV_1 and clinical laboratory values during treatment and at the final visit (visit 7)

Additional secondary efficacy endpoints during the efficacy assessment period are:

- Average rhinoconjunctivitis DSS
- Average rhinoconjunctivitis DMS
- PRQLQ score
- Average asthma DSS
- Average daily use of SABA
- Rhinitis mild days
- Rhinitis exacerbation days (days with a rhinitis DSS of 6 or of 5 with one individual symptom scored 3 (symptom that is hard to tolerate; causes interference with activities of daily living and/or sleeping))
- Average CSMS (EAACI task force recommended combined symptom and medication score **(Pfaar et al. 2014)**)

The immunology endpoints are:

- Change from baseline in specific IgE and IgG4 to D. *pteronyssinus* and D. *farina*, change in baseline for HDM IgE-Blocking factor (IgE-BF) and change in baseline for total IgE measured at end of trial (visit 7)

2.8 Exploratory endpoints

The exploratory endpoints during the efficacy assessment period are:

- Average TCCS (the sum of the conjunctivitis DSS and the conjunctivitis DMS)
- Average conjunctivitis DSS
- Average conjunctivitis DMS
- Rhinitis symptom-free days
- Nocturnal awakenings due to asthma requiring SABA use

The exploratory endpoints derived from the 2 weeks daily eDiary collected 8 and 16 weeks after treatment initiation are:

- Average TCRS
- Average TCS
- Average rhinitis DSS
- Average rhinitis DMS
- TCRS during pollen season in spring and summer

3 Trial design

3.1 Summary of trial design

This trial is an approximately one-year randomised, parallel-group, double-blind, placebo-controlled multi-national phase III trial conducted in Europe and North America. Approximately 1370 subjects will be randomised (1:1) to receive treatment with the HDM SLIT-tablet or placebo, see Figure 2.

The trial evaluates the efficacy and safety of the HDM SLIT-tablet in children (5-11 years) with HDM AR/C with or without asthma. The treatment duration is approximately 12 months for all subjects. The last 8 weeks of the treatment phase will be used as the primary efficacy assessment period, which is anticipated to occur when allergy symptoms due to HDM exposure are predominant due to limited interference by pollen exposure.

Figure 2 Trial design

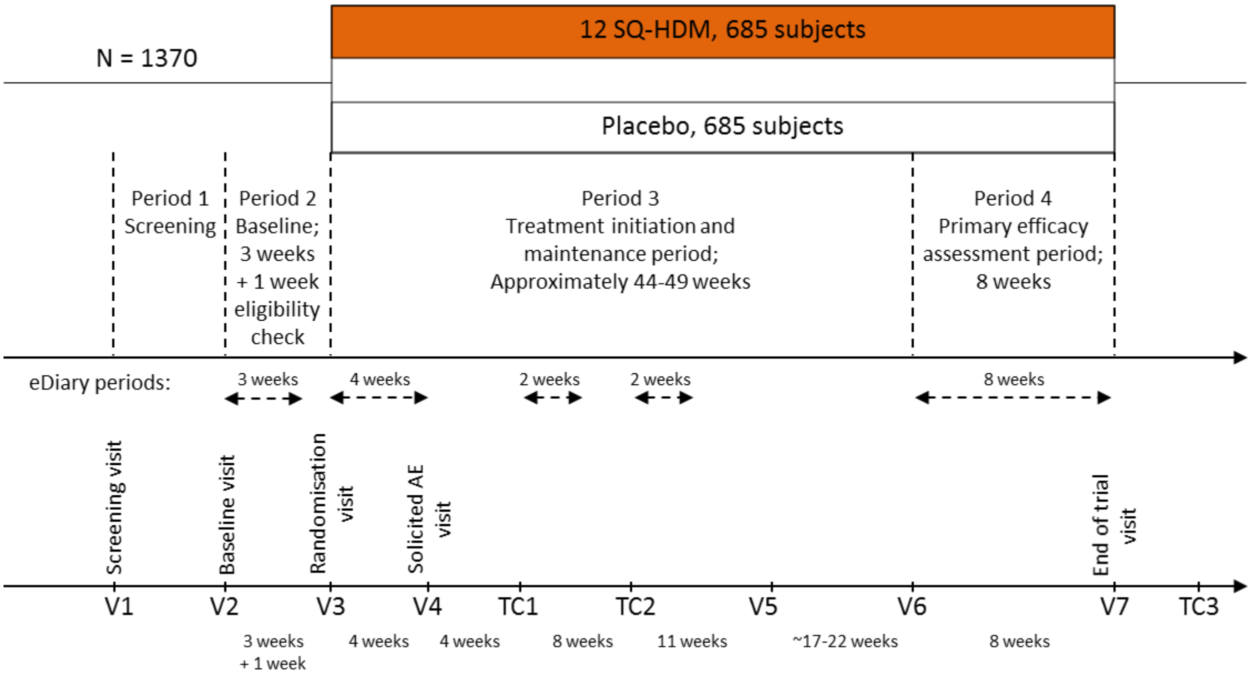

The trial consists of 4 periods: screening (Period 1), a baseline period (Period 2), a treatment initiation and maintenance (Period 3), and a primary efficacy assessment period during the last 8 weeks (period 4) of the approximately 12 months treatment.

Period 1 is the screening period. Subjects may be screened up to 12 weeks before randomisation.

Period 2 is the baseline period and lasts 21 days plus 7 days for eligibility check. Subjects' baseline must be recorded between 1 September and 1 April. In addition, pollen allergic subjects must have their baseline period outside the season of their pollen allergy. During the baseline period, subjects are allowed to take sponsor provided rhinoconjunctivitis and asthma rescue medication, and their rhinoconjunctivitis symptoms and use of rescue medication will be rated on a daily basis in their eDiary. To be eligible for inclusion, subjects will be required to have a rhinitis DSS of at least 6, or a score of at least 5 with one symptom being severe, on 8 of the last 14 days of the baseline period and will be required to use rescue medication for treatment of HDM allergic rhinitis during at least 8 of the last 14 days of the baseline period. The baseline period must end 1 week before randomisation to allow for eligibility check. Subjects with evidence of current, clinically significant, intercurrent illness (e.g., significant common cold or influenza) during the baseline period may be rescheduled for a repeat of the baseline on resolution of their illness. Please note that the blood samples for safety testing needs to be repeated if the results are more than 12 weeks old.

Period 3 is the treatment initiation and maintenance period. It begins at randomisation (visit 3) and lasts for approximately 44-49 weeks. During the first 28 days of period 3, 15 pre-specified symptoms/signs (based on recommendation by the WAO **(Passalacqua et al. 2013)**) occurring after IMP intake will be recorded by the parent/caregiver together with the subject in the eDiary. If pre-specified symptoms are reported in the eDiary, the investigator will evaluate these and

report them in the eCRF as solicited AEs at visit 4. To evaluate efficacy, there will be a 2 weeks efficacy assessment period recorded in the eDiary at week 8 and week 16.

Period 4 is the 8-week primary efficacy assessment period beginning at visit 6 (approximately 44-49 weeks after randomisation) and lasting until visit 7. Subjects' primary efficacy assessment period must be between 1 September and 1 April. The 8-week primary efficacy assessment period must be planned to include the dates that were included in the 3-week baseline period the previous year. In addition, pollen allergic subjects must have their primary efficacy assessment period outside the season of their pollen allergy. During period 4, the parent/caregiver together with the subject will rate the rhinoconjunctivitis symptoms and use of rescue medication on a daily basis in the eDiary.

3.2 Trial schedule

	Cohort 1	Cohort 2	Cohort 3
First subject first visit (FSFV)	H2 Year 1	Q3 Year 2	Q3 Year 3
Last subject randomised	01-April Year 2	01-April Year 3	01-April Year 4
Last subject last visit (LSLV)	Q2 Year 3	Q2 Year 4	Q2 Year 5

Duration of treatment per subject: approximately 12 months.

End of trial is defined as LSLV for the last cohort initiated.

3.3 Discussion of design

This trial is a randomised, parallel-group, double-blind, placebo-controlled multi-national trial based on the EMA CHMP guideline on the clinical development of medicinal products for the treatment of allergic rhinoconjunctivitis (**EMEA 2004**) and the FDA guidance for industry on developing drug products for treatment of allergic rhinitis **(FDA 2018)**, and incorporates the recommendations of the EMA CHMP guideline on the clinical development of products for specific immunotherapy for the treatment of allergic diseases **(EMEA 2008)**, the needs stated in the World Health Organisation (WHO) position paper on allergen immunotherapy for development of sublingual immunotherapy (**Bousquet et al. 1998**), the EMA/PDCO standard paediatric investigation plan for allergen products for specific immunotherapy (**EMA & PDCO 2015**), and the FDA feedback on the trial protocol.

The trial design and clinical efficacy endpoints are based on the guidelines referenced above, and on the experience from previous ALK trials with allergy immunotherapy, in general, and with the HDM SLIT-tablet in particular for treatment of AR.

The trial population comprises children (5-11 years) with a clinical history of persistent HDM AR/C with or without asthma. This trial population reflects a population that is suitable for treatment with allergy immunotherapy. Subjects must report a level of symptoms consistent with HDM AR during the baseline period to be eligible for the trial. The required symptom level is based on the previous European MT-06 trial that assessed the efficacy of the HDM SLIT-tablet in adult subjects **(Demoly et al. 2016)**, and is similar to what was used in the North American P001 trial that assessed the efficacy in adults and adolescents **(Nolte et al. 2016)**.

The duration of treatment is approximately 12 months for all subjects. The duration of treatment is related to the fact that HDM AR/C is a perennial allergy, typically with an increase in symptoms during the winter months. In addition, the treatment duration was chosen to mirror

the design of previous phase III rhinitis trials with the HDM SLIT-tablet (MT-06, P001, TO-203-3-2, TO-203-3-3), in which primary efficacy was assessed after approximately 12 months of treatment **(Demoly et al. 2016; Nolte et al. 2015)**. The approximately 12-month treatment period will also provide long-term safety assessment.

For the primary objective, efficacy measurements are to be performed during the autumn, winter or spring where HDM allergic patients typically have more symptoms (**Bousquet et al. 2008**) and where the effect of potentially confounding pollen seasons is limited. Hence, the primary efficacy assessment period is 8 weeks at the end of the 12-month treatment period, while 2-weeks efficacy measurement periods will be placed after telephone visit 1 (8 weeks after randomisation) and telephone visit 2 (16 weeks after randomisation). The 8-week primary efficacy assessment period must not overlap with the local pollen season if the subject is allergic to pollen.

The primary endpoint is the TCRS, which is the sum of the rhinitis DSS and rhinitis DMS, during the 8-week primary efficacy assessment period at the end of the approximately 12-month treatment period. This combined symptom and medication score is considered to provide a relevant measure of clinical benefit experienced by the subject **(Canonica et al. 2007)**. The primary endpoint, presented as a single combined variable, is consistent with current global regulatory requirements for appropriate reporting of results from allergy immunotherapy trials. Specifically, EMA has stated in their guidance on allergy immunotherapy that the primary endpoint for trials should be reflective of both the severity of symptoms and the intake of rescue medications. In addition, WAO has stated that symptoms and rescue medication utilisation are interdependent variables. Therefore, in assessing the effect of allergy immunotherapy, a single pre-defined combined daily symptom and medication score more accurately reflects the clinical benefit experienced by the subject.

The minimal clinically relevant difference between active and placebo for the primary endpoint is defined as 15% in this trial, which is similar to what was observed in the North American phase III trial (P001) in adolescents and adults **(Nolte et al. 2016)** and in line with FDA considerations on clinical relevant efficacy **(Massie 2011)**.

Safety endpoints include AEs, including ESIs such as eosinophilic oesophagitis, vital signs and laboratory values that are normally used to characterise the safety profile of an IMP. In addition, 15 specific symptoms, identified as local side effects of sublingual immunotherapy (based on WAO recommendations (**Passalacqua et al. 2013**)), will be collected in the subjects' eDiary for the first 28 days after randomisation. The reported symptoms will be evaluated by investigators and reported in the eCRF as solicited AEs.

The active dosage chosen in the trial is 12 SQ-HDM, which is the approved dose for adults in the United States, Canada, Europe, Australia, New Zealand, and Southeast Asia, and for adolescents (12-17 years) in Europe, Australia, and Southeast Asia. The dose is based on phase I safety data in children and the appropriate dose established in adults as the dose providing the highest efficacy with the fastest onset of effect and with a safety profile that supports daily at-home administration **(Demoly et al. 2016; Nolte et al. 2015; Virchow et al. 2016**). This dose (12 SQ-HDM) will constitute the relevant dose for all populations ≥5 years of age, in accordance with the general practice for AIT (**Alvarez-Cuesta et al. 2006**).

Randomisation introduces a deliberate element of chance into the assignment of treatments to subjects in a clinical trial. During subsequent analysis of the trial data, it provides a sound statistical basis for the quantitative evaluation of the evidence relating to treatment effects. Randomisation also tends to produce treatment groups in which the distributions of prognostic

factors, known and unknown, are similar. In combination with blinding, randomisation helps to avoid potential bias in the selection and allocation of subjects arising from the predictability of treatment assignments (**ICH Harmonised Tripartite Guideline 1998**). It is desirable for statistical purposes to compare groups of equal size and therefore the randomisation procedure is planned as a 1:1 randomisation.

A double-blinded set-up has been chosen to minimise the potential biases resulting from differences in management, treatment, or assessment of subject, or interpretation of results that could arise as a result of subject or investigator knowledge of the assigned treatment.

A placebo arm has been introduced to establish the superiority of the HDM SLIT-tablet compared to placebo with respect to reducing the TCRS. Placebo is used as comparator since no well-established active comparator exists. All subjects will have access to open-label rescue medication which is the standard of care for the treatment of allergic AR/C.

4 Trial population

The trial population will include approximately 1370 children aged 5-11 years with HDM AR/C with or without asthma. The subjects will be randomised 1:1 to receive treatment with the HDM SLIT-tablet (12 SQ-HDM) or placebo.

Subjects who meet all of the inclusion criteria listed in section 4.1 and none of the exclusion criteria listed in section 4.2 will be considered eligible for the trial.

4.1 Inclusion criteria

I1. Written informed consent obtained from parents/guardians before any trial related procedures are performed[18]

I2. Male or female of any race/ethnicity and weighing 15 kg or more on the day of screening

I3. 5-11 years old at randomisation

I4. A clinical history of HDM AR/C when exposed to HDM (diagnosed by a physician) of 1 year duration or more (with or without asthma) and with allergic rhinitis symptoms despite having received allergy pharmacotherapy during the previous year prior to the screening visit[19]

I5. Have a rhinitis DSS of at least 6, or a score of at least 5 with one symptom being severe, on at least 8 of the last 14 days of the baseline period

I6. Use symptomatic medication for treatment of HDM allergic rhinitis during at least 8 of the last 14 days of the baseline period

I7. Presence of one or more of the following Allergic Rhinitis Impact on Asthma (ARIA) quality of life items due to HDM AR/C during the last 14 days of the baseline period:

1) Sleep disturbance

2) Impairment of daily activities, leisure and/or sport

[18] Assent from subject must be obtained according to national requirements.
[19] If medical records are not available, verbal history from subject/parent/caregiver can be used to fulfill this criterion and this must be documented in the medical records by the investigator.

3) Impairment of school

4) Troublesome symptoms

I8. Positive SPT to *D. pteronyssinus* or *D. farinae* at screening[20]

I9. Positive *D. pteronyssinus* or *D. farinae* specific IgE (defined as ≥class 3, ≥3.5 kU/l) at screening

I10. Lung function measured by FEV1 ≥ 70% of predicted value or according to local requirements at randomisation (visit 3). For subjects having a diagnosis of asthma, FEV1 is measured while on subject's usual asthma medication following at least a 6-hour washout of SABA. The criteria must be fulfilled, unless subject is ≤7 years old and not able to perform reproducible FEV_1 manoeuvres despite coaching and is not considered as having a diagnosis of asthma as defined in section 11.5 of this protocol[21].

I11. Subject willing and able to comply with trial protocol

4.2 Exclusion criteria

E1. A clinically relevant history of symptomatic perennial AR/C caused by a perennial allergen source such as animal hair and dander and/or mould to which the subject is exposed during the baseline and/or efficacy assessment periods

E2. A clinically relevant history of symptomatic seasonal allergic rhinoconjunctivitis and/or asthma caused by an allergen to which the subject is exposed, and which could potentially overlap with the baseline and/or efficacy assessment periods

E3. Any nasal or pharyngeal condition that could interfere with the safety or efficacy evaluation, (e.g., clinically relevant nasal polyps, a history of paranasal sinus surgery or surgery of nasal turbinates)[22]. Surgery of the palatine or pharyngeal tonsils in the past is not an exclusion criterion

E4. SLIT treatment with *D. pteronyssinus* or *D. farinae* for more than 1 month within the last 5 years. In addition, any SLIT treatment with *D. pteronyssinus* or *D. farinae* within the previous 12 months

E5. SCIT treatment with *D. pteronyssinus* or *D. farinae* reaching the maintenance dose within the last 5 years. In addition, any SCIT treatment with *D. pteronyssinus* or *D. farinae* within the previous 12 months

E6. Ongoing treatment with any allergy immunotherapy product

E7. Severe chronic oral inflammation

---

[20] A positive SPT is defined in the SPT Guideline. Briefly, for subjects in North America, a positive SPT is defined as a wheal size ≥5 mm. For subjects in Europe, a positive SPT is defined as a wheal size of ≥3 mm. If medication that could interfere with the SPT, according to Table 8 has not been washed out, the SPT must be repeated after the interfering medication has been washed out.

[21] As defined in section 11.5 subjects are considered as having a diagnosis of asthma if at least one of the following 4 criteria are met:

- At least one episode of wheeze, cough, shortness of breath or chest tightness and a change in FEV_1 ≥12% after beta2-agonist administration
- Recurrent wheeze with or without prolonged phase of forced exhalation observed at physical examination and an intake of asthma medication which resulted in a clinically relevant effect
- Wheezing with or without prolonged phase of forced exhalation observed at physical examination and a change in FEV_1 ≥12% after beta2-agonist administration
- Using asthma medication

[22] If in doubt, nasal endoscopy should be performed

E8. A diagnosis or history of eosinophilic oesophagitis

E9. Any systemic immunosuppressive treatment, other than glucocorticosteroids, within 130 days prior to visit 1 (screening). Any oral glucocorticosteroids from 60 days prior to visit 2 (baseline). Any other systemic glucocorticosteroids (depot or parenteral) from 90 days prior to visit 2 (baseline) (see Table 2)

E10. Asthma requiring daily use of more than 400 mcg budesonide or equivalent (see Table 4) at screening

E11. Any clinical deterioration of asthma that resulted in emergency treatment, hospitalisation or treatment with systemic corticosteroids within 3 months prior to randomisation

E12. Any clinically relevant chronic disease, including malignancy, that in the opinion of the investigator would interfere with the trial evaluations or the safety of the subject

E13. A history of chronic urticaria (> 6 weeks) and/or chronic angioedema (> 6 weeks) within the last 2 years prior to screening that in the opinion of the investigator may constitute an increased safety concern

E14. A relevant history of systemic allergic reaction e.g. anaphylaxis with cardiorespiratory symptoms, generalised urticaria or severe facial angioedema that in the opinion of the investigator may constitute an increased safety concern

E15. Active or poorly controlled autoimmune diseases, immune defects, immunodeficiencies, immunosuppression or malignant neoplastic diseases with current disease relevance

E16. Treatment with medications with potential impact on efficacy endpoints (e.g. treatment with anti-IgE drugs within 130 days/5 half-lives of the drug (which ever longest) or treatment with antidepressant or antipsychotic medications with antihistaminergic effect)

E17. Known history of allergy, hypersensitivity or intolerance to any of the excipients or active substances of the IMP (except for *D. pteronyssinus* and/or *D. farinae*) or to any excipient of the rescue medication provided in this trial

E18. Female with positive urine pregnancy test

E19. Sexually active female of childbearing potential without medically accepted contraceptive method[23]

E20. A business or personal relationship with trial staff or sponsor who is directly involved with the conduct of the trial

E21. Previously been randomised into this trial, is participating in this trial at another investigational site or is participating or planning to participate in any other clinical trial during the duration of this trial

E22. A history or current evidence of any condition, treatment, laboratory values out of range or other circumstance that in the opinion of the investigator are clinically relevant and might expose the subject to risk by participating in the trial, confound the results of the trial, or interfere with the subject's participation for the full duration of the trial

---

[23] For the purpose of this protocol the following contraceptive methods are considered appropriate: oral contraceptives, trans dermal patches or depot injection of a progestogen drug (starting at least 4 weeks prior to IMP administration); double barrier method: condom or occlusive cap (diaphragm or cervical/vault caps) plus spermicidal agent; intrauterine device (IUD), intrauterine system (IUS), implant, or vaginal ring (placed at least 4 weeks prior to IMP administration); or male partner sterilisation (vasectomy with documentation of azoospermia) prior to the female subject's entry into trial and is the sole sexual partner for that female subject. Sexual abstinence is acceptable as contraceptive if it is true abstinence and in line with the usual lifestyle of the subject. Periodic abstinence (e.g., calendar, ovulation, symptothermal, post-ovulation methods) and withdrawal are not acceptable methods of contraception. However, national requirements regarding contraception should always be followed.

E23. Has a condition or requires treatment that may increase the risk of the subject developing severe adverse reactions after adrenaline/epinephrine administration

E24. Unable to or will not comply with the use of adrenaline/epinephrine auto-injectors for countries where this is a regulatory requirement

5 Subject discontinuation

5.1 Discontinuation from IMP treatment

Subjects must be discontinued from IMP treatment and should continue in the trial for safety assessments under the following circumstances:

- If the subject becomes pregnant
- The subject experiences severe or persistent symptoms of oesophagitis or have a confirmed diagnosis of eosinophilic oesophagitis
- If, in the investigator’s opinion, continuation with IMP treatment would be detrimental to the subject’s well-being

5.2 Discontinuation from trial

The subject and the subject's parent/guardian will be advised in the informed consent form that he/she has the right to withdraw from the trial at any time without prejudice. Where discontinuation from the trial is initiated by the subject/parent/guardian, the investigator is to ascertain the primary reason for discontinuation from the list below:

- An AE for which the investigator did not consider discontinuation from the trial necessary
- Perceived insufficient therapeutic effect
- Co-existing disease
- Withdrawal of consent
- Other reasons

Additionally, the subject may at any time be discontinued from the trial at the discretion of the investigator or sponsor.

Subjects must be discontinued from the trial under the following circumstances:

- If, in the investigator’s opinion, continuation in the trial would be detrimental to the subject’s well-being
- If subject is lost to follow-up
- If informed consent is withdrawn
- If treatment is unblinded for the subject or investigator

If found required by the sponsor after discussion with the investigator, subjects may be discontinued from the trial under the following circumstances:

- If subject is treated with prohibited medication as defined in Table 2
- In case of protocol deviation, violation of eligibility criteria or deviation from the treatment plan specified in the protocol (e.g. incorrect administration of the IMP)

In case a subject discontinues from the trial, the discontinuation page in the eCRF should be completed. On the discontinuation page the investigator should record the date of the discontinuation from trial, the person initiating the discontinuation and the primary reason for discontinuation. If an AE is involved in a discontinuation, this must be recorded as the primary reason. In all cases, the primary reason for discontinuation must be recorded in the eCRF and in the subject's medical records. Follow-up on the subject is necessary to establish whether the reason was an AE. If so, this must be reported in accordance with the appropriate procedures.

To the extent possible, all examinations scheduled for the final visit must be performed on subjects who receive IMP but will not complete the trial according to the protocol. In addition, the TC follow-up (TC 3) should be performed and the corresponding eCRF pages should be filled in.

Reasonable effort should be made to contact any subject lost to follow-up during the course of the trial in order to complete assessments and retrieve any outstanding data and medication/supplies. If the subject is lost to follow-up, the effort taken to contact the subject must be documented in the subject's medical record.

Data obtained until discontinuation will be used for statistical analyses.

6 Randomisation and treatment blinding/unblinding

6.1 Subject ID number

All subjects enrolled must be identifiable throughout the trial. Thus, at the screening visit all subjects will receive a unique subject number. The subject number will be generated when the subjects' data are entered into the eCRF.

6.2 Randomisation

The randomisation list will be generated by a trial-independent statistician and will not be accessible to trial personnel involved in the conduct of the trial, until the database has been locked.

When a subject is randomised a unique number is assigned for the first dispensing of IMP.

6.3 Subject card

All randomised subjects who have signed the informed consent form will be given a subject card by the investigator or qualified designee, identifying them as participants in a clinical trial. The card will contain trial site information (including direct telephone number) to be utilised in the event of an emergency.

6.4 Treatment blinding/unblinding

A double-blind set-up will be used. The HDM SLIT-tablet and the matching placebo will be similar in appearance and taste and will be packaged identically to maintain the treatment blind. The subject/parents/guardian, the investigator/site staff and sponsor personnel or delegate(s) who are involved in the treatment administration or clinical monitoring of the subject data will be blinded in terms of which IMP treatment group (active or placebo) the subject is allocated. DMC members will be unblinded to treatment.

The randomisation code for a particular subject can be broken in a medical emergency if knowledge of the IMP is necessary for the optimal treatment of the subject. If possible, the trial site must contact sponsor prior to unblinding the subject's treatment. However, in case of an emergency necessitating the knowledge of the IMP, unblinding and appropriate treatment should be the very first action by the site.

The randomisation code breaking will be performed via Interactive response technology (IRT) for this trial. The time, date and reason for unblinding as well as the initials of the person breaking the randomisation code must be recorded.

The IRT will notify the CRA and the sponsor safety department immediately after the randomisation code is broken.

The subject must be discontinued from the trial if subject or investigator is unblinded.

It may also be necessary to unblind an individual subject’s treatment by the sponsor safety department for the purposes of expedited reporting to the authorities and/or ethics committees/review boards (e.g. in case of a suspected unexpected serious adverse reactions (SUSAR)). In that situation, blinding of other sponsor personnel should be maintained during the trial.

7 Restricted and prohibited concomitant medication

Restricted and prohibited concomitant medications are listed below.

All concomitant medications must be appropriately documented in the eCRF. The use of any concomitant medication must relate to the documented medical history or an AE of the subject.

The medication listed in Table 2 is restricted or prohibited unless it is provided by the sponsor.

Table 2 Restricted and prohibited concomitant medications

Drug	Time window	Reason
An investigational drug other than the IMP	30 days/5 half-lives of the drug (whichever longest) before visit 1 and until end of trial	Possible interaction between IMPs.<br>Interferes with efficacy and safety evaluations
Anti-IgE treatment, e.g. omalizumab	< 130 days/5 half-lives of the drug (whichever longest) prior to visit 1 and until end of trial	Interferes with rhinoconjunctivitis and/or asthma efficacy assessments

Drug	Time window	Reason
Antihistamine, unless provided by the sponsor		
• Oral, intravenous, nasal or ocular	From visit 2 and until end of trial	Interferes with rhinoconjunctivitis efficacy assessments<br>Interferes with safety evaluation
• Long-acting (astemizole)	≤ 90 days before visit 2 and until end of trial	Interferes with rhinoconjunctivitis efficacy assessments.<br>Interferes with safety evaluation
Antidepressant medications:<br>• Antidepressant medication with antihistaminic effect (e.g. doxepin, mianserine)<br>• Tricyclic antidepressants (e.g. amitriptyline, clomipramine)	≤ 14 days before visit 1 and until end of trial	Interferes with rhinoconjunctivitis efficacy assessments<br>Interferes with safety evaluation. Tricyclic antidepressants may potentiate the effect of adrenaline/epinephrine
Antipsychotic medications with antihistaminic effects (e.g. chlorpromazine, levomepromazine, clozapine, olanzapine, tioridazine)	≤ 7 days before visit 1 and until end of trial	Interferes with rhinoconjunctivitis efficacy assessments due to antihistaminic effect<br>Interferes with safety evaluation
Glucocorticosteroids, unless provided by the sponsor		
• topical (nasal or ocular)	From visit 2 and until end of trial	Interferes with rhinoconjunctivitis efficacy assessments.<br>Interferes with safety evaluation
• oral (tablets)	From 60 days before visit 2 and until end of trial	Interferes with asthma and rhinoconjunctivitis efficacy assessments.

Drug	Time window	Reason
		Interferes with safety evaluation
• systemic (depot formulations parenteral administration, regardless of treatment days and dose)	≤ 90 days before visit 2 and until end of trial	Interferes with asthma and rhinoconjunctivitis efficacy assessments.<br>Interferes with safety evaluation
Systemic immunosuppressive treatment, other than glucocorticosteroids (ATC code L04, L01, R03, D11) (e.g. dupilumab, mepolizumab, reslizumab, lebrikizumab)	≤ 130 days or 5 half-lives (which ever longest) before visit 1 and until end of trial	Interferes with rhinoconjunctivitis and/or asthma efficacy assessments
Immunotherapy with any other allergen(s)	From visit 1 and until end of trial	Interferes with rhinoconjunctivitis and/or asthma efficacy assessments.<br>Interferes with safety evaluation
Inhaled, topical or oral nedocromil or cromolyn sodium	≤ 14 days before visit 2 and until end of trial	Interferes with rhinoconjunctivitis and/or asthma efficacy assessments
Long-acting muscarinic antagonists (e.g. Tiotropium)	From 30 days before visit 2 and until end of trial	Interferes with asthma efficacy assessments
Methylxantin (e.g. theophylline)	From visit 2 and until end of trial	Interferes with asthma efficacy assessments
LTRA (e.g. montelukast, zafirlukast)	From visit 1 and until end of trial. If treatment has begun at least 30 days prior to visit 1 dose can be kept unchanged during trial	Interferes with rhinitis and asthma efficacy assessments
Nasal decongestants	From 7 days before each eDiary period and during the eDiary period, beginning from visit 2 and until	Interferes with rhinitis efficacy assessments

Drug	Time window	Reason
	end of trial	
Pizotifene	≤ 7 days before visit 1 and until end of trial	Interferes with rhinoconjunctivitis efficacy assessments due to antihistaminic effect
SABA, unless provided as rescue medication in the trial	From visit 2 and until end of trial	Interferes with asthma efficacy assessments<br>Interferes with safety evaluation

8 Trial products

8.1 IMP

The IMP provided in the trial is HDM SLIT-tablet or placebo.

The IMP will be provided by the sponsor. The treatment will begin at visit 3 (randomisation visit), where the first IMP will be dispensed. Hereafter, IMP will be dispensed at the visits according to the flow chart. When the first dose is administered, the subject will be under medical supervision for at least 30 minutes after the tablet intake. For the recording of onset and duration of solicited AEs that occur on the first day of IMP administration, the subject should remain in the clinic for 30 minutes after IMP intake.

The IMP is manufactured by the sponsor. The placebo tablets are similar to the active tablet with respect to appearance, smell and taste.

Active treatment

Active ingredients:	Standardised allergen extract from the HDMs *D. pteronyssinus* and *D. farinae*
Dosage form:	Oral lyophilisate
Dose/strength:	12 SQ-HDM
Excipients:	Gelatine (fish source), mannitol and sodium hydroxide

Placebo treatment

Active ingredients:	None
Dosage form:	Oral lyophilisate
Excipients:	Gelatine (fish source), mannitol and sodium hydroxide

8.2 Rescue medication

During the trial, subjects may experience allergy symptoms that require additional treatment. The rescue medication must be used in addition to the IMP to which the subjects have been randomised.

Rescue medication will be dispensed at the baseline visit. If needed, spacer will be re-imbursed by sponsor. The rescue medication will be re-supplied as described in the Flowchart (Table 1) when needed.

Rhinoconjunctivitis rescue medication

Rhinoconjunctivitis rescue medication in this trial will be provided by the sponsor as pre-defined, open-labelled rescue medication.

The subject will be provided with:

- Oral antihistamine tablets (Loratadine tablets 10 mg) or
- Oral antihistamine solution (Desloratadine oral solution 0.5 mg/ml)
- Antihistamine eye drops (Olopatadine 1 mg/ml)
- Nasal corticosteroid spray (Mometasone furoate 50 micrograms/dose)

The rhinoconjunctivitis rescue medication provided to the subject must be used according to the product's labelling (e.g. SmPC or USPI). The dosage instructions are described in Table 3.

Table 3 Schedule for rhinitis and conjunctivitis rescue medication

Allergy rescue medication	Subject dosage instructions
Desloratadine solution, 0.5 mg/ml	5 years old: 2.5 ml solution once daily as needed
	6-11 years old: 5.0 ml solution once daily as needed
	≥12 years old: 10 ml solution once daily as needed
Loratadine tablets, 10 mg	6-12 years old weighing more than 30 kg: 1 tablet once daily. 10 mg tablets are not suitable for children who weigh less than 30 kg.
	>12 years old: 1 tablet once daily as needed
Mometasone furoate 50 micrograms/dose	5-11 years old: 1 puff in each nostril once daily as needed
	≥12 years old: 2 puffs in each nostril once daily as needed
Olopatadine eye drops, 1 mg/ml	1 drop in the affected eye(s) twice daily, morning and evening as needed

Rescue medication for asthma symptoms

Subjects with a diagnosis of asthma, according to the criteria stated in 11.5, will be dispensed rescue medication for asthma.

SABA (i.e., albuterol/salbutamol inhaler) will be provided locally by the trial site and reimbursed by sponsor. If not possible due to local country operational or regulatory requirements, it will be supplied locally by sponsor designee or centrally by the sponsor. Subjects with asthma will receive SABA at visit 2 and at subsequent visits (including unscheduled visits) as necessary.

Other medications for asthma will not be reimbursed.

Subjects who are taking low or medium daily dose inhaled corticosteroids (with or without LABA) for asthma management will be allowed to continue with the same medications during the trial. A subject must have been on a stable regimen (dose unchanged) for at least 2 weeks prior to screening (visit 1) and 4 weeks before randomisation (visit 3). Low and medium daily doses of ICS are defined in Table 4.

If a subject requires additional dose adjustment of asthma medications, the subject should follow recommendations regarding medication adjustments provided by his/her physician. Medication use should be recorded in the eCRF.

Table 4 Definition of low, medium and high dose ICS for subjects aged 5-11 years

Inhaled corticosteroid	Low daily dose (micrograms)	Medium daily dose (micrograms)	High daily dose (micrograms)
Beclomethasone dipropionate	100 - 200	> 200 – 400	> 400
Budesonide DPI	100 - 200	> 200 – 400	> 400
Budesonide nebs	250 - 500	> 500 – 1000	> 1000
Flunisolide	500 - 750	> 750 – 1250	> 1250
Fluticasone propionate	100 - 200	> 200 – 500	> 500
Ciclesonide	80 - 160	> 160 – 320	> 320
Mometasone furoate	100	200	> 200
Triamcinolone acetonide	400 - 800	> 800 – 1200	> 1200

Rescue medication for severe allergic reaction (adrenaline/epinephrine auto-injector)

In countries where it is a regulatory requirement, two adrenaline/epinephrine auto-injectors will be provided to each subject and parent/caregiver at the randomisation visit (visit 3) and should

be available around the time that the tablets are administered at-home. Adrenaline/epinephrine auto-injector, dose selection should follow SmPC/USPI for the product.

Adrenaline/epinephrine auto-injectors are intended for immediate self-administration for an anaphylactic reaction, including symptoms/signs of upper airway obstruction (see section 9.3 for further details).
Adrenaline/epinephrine auto-injectors will be supplied by the investigators and will be reimbursed by the sponsor.

8.3 Packaging and labelling

The IMP will be supplied in blister cards containing 10 tablets each. The blister cards will be packed in specific boxes containing a sufficient number of tablets to cover the treatment period between the dispensing visits and the end of trial visit.

IMP will be packaged and labelled according to EU Annex 13 and national requirements. The IMP will be uniquely numbered.

Rescue medication will be sourced as commercially available products in a member state of the EU. The products will be labelled with an additional label including trial specific information.

Rescue medication supplied by the sponsor will be dispensed together with patient information leaflets in local language.

Packaging and labelling will be outsourced.

8.4 Handling and storage

The trial products provided by the sponsor (IMP, rhinoconjunctivitis rescue medication, SABA and SPT materials) are to be used only for this trial and not for any other purpose.

IMP, rhinoconjunctivitis rescue medication, SABA and SPT must be stored in a secure, limited-access location separately from normal clinic stocks and according to label specifications. IMP and rescue medication returned by the subject must be stored separately from other medication, e.g. unused IMP that has not yet been dispensed.

Site storage conditions for IMP, rhinoconjunctivitis rescue medication, SABA and SPT must be monitored by the site staff for adherence to label specifications and reviewed by the CRA during monitoring visits.

Monitoring must be done using a calibrated, stationary and continuously recording system. As a minimum a calibrated min/max thermometer is required.

8.5 IMP and rescue medication accountability

The investigator or appropriate delegated staff must maintain records of the IMP, rhinoconjunctivitis rescue medication and SABA delivered to the trial site from the sponsor. The site must maintain records of:

- Inventory at the site
- Dispensing to each subject

- Return by each subject to site
- Return by site to the sponsor

These records must include dates, quantities, batch/serial numbers, expiry dates and the IMP unique code numbers assigned to the trial subject. Investigators must maintain records that document adequately that the subjects were provided the doses specified by the protocol and must reconcile all IMP and rescue medication received from the sponsor.

All IMP and rhinoconjunctivitis rescue medication accountability logs and records will be verified by the CRA during the monitoring visits in accordance with the monitoring plan.

Full drug accountability will be performed for the IMP by tablet count. Full drug accountability will not be performed on rescue medication. After LSLV at site, the investigator will return all unused and partly used IMP and rhinoconjunctivitis rescue medication and a copy of the completed drug accountability form to the CRA or to the sponsor address provided. The investigator must not destroy any IMP or rhinoconjunctivitis rescue medication without written agreement with the sponsor.

8.6 Reporting of technical complaints

Any technical complaint related to the trial products provided by the sponsor (IMP, rhinoconjunctivitis rescue medication, SABA or SPT) must be reported to the sponsor. The information must be accompanied by samples or a picture of the item.

9 Treatment

9.1 Posology and method of administration

The SLIT-tablet should be taken with dry fingers from the blister unit immediately after opening the blister and placed under the tongue, where it will disperse. Swallowing should be avoided for approximately 1 minute.

Food and beverages should not be taken for 5 minutes after intake of IMP. The daily dose of IMP is 1 SLIT-tablet, which should preferably be taken in the morning.

9.2 Precautions in relation to first dosing

First intake of IMP should be at the clinic with a minimum of 30 minutes observation period after the intake. For the recording of onset and duration of solicited AEs that occur on Day 1, the subject should remain in the clinic for 30 minutes after IMP intake.

Prior to IMP intake, an oropharyngeal examination should be performed. This should be repeated 30 ±5 minutes after IMP intake.
For subjects with severe oral inflammation (e.g. oral lichen planus, mouth ulcers or thrush), oral wounds or following oral surgery, including dental extraction, or following tooth loss, initiation of IMP treatment should be postponed until the oral cavity has healed.

For subjects with symptoms of, or in treatment for upper respiratory tract infection, asthma exacerbation, acute sinusitis, acute otitis media or other relevant infections, initiation of IMP treatment should not be initiated until the condition has improved.

9.3 Rescue medication for severe allergic reactions

At visit 3, each subject and parent/caregiver will be provided with educational information regarding symptoms of local and systemic allergic reactions, including anaphylaxis. The investigator will provide written instructions and explain to the subject and parent/caregiver, when to administer the adrenaline/epinephrine auto-injectors for countries where this is a regulatory requirement. The investigator will complete a written local and systemic allergic reaction emergency plan **(Epstein et al. 2017)**, including treatment options. A list of symptoms that may be present during anaphylaxis include flushing, apprehension, syncope, tachycardia, thread or unobtainable pulse associated with a fall in blood pressure, vomiting, diarrhea and abdominal cramps, wheezing, dyspnea due to laryngeal spasm, lower airway obstruction, pruritus, rashes (such as urticaria), or angioedema.

In countries where it is a regulatory requirement, the subject /parent/caregiver will be provided with two adrenaline/epinephrine auto-injectors at the randomisation visit (visit 3). Adrenaline/epinephrine auto-injectors are intended for immediate self-administration for an anaphylactic reaction, including symptoms/signs of upper airway obstruction. The investigator will instruct the subject and parent/caregiver on how to use the adrenaline/epinephrine auto-injectors and to have it available when the tablet is administered.

In the event that the adrenaline/epinephrine auto-injector is used, the subject/parent/caregiver must immediately call the local emergency number. The subject/parent/caregiver must inform the investigator and an unscheduled visit should be arranged to further evaluate the subject.

At each visit following the dispensing, the investigator or designee will verify that the subject has adrenaline/epinephrine auto-injectors and will review instructions for use. Unused adrenaline/epinephrine auto-injectors will be collected at visit 7.

9.4 Temporary interruption and discontinuation of treatment

Treatment may be discontinued for up to 7 days for the following reasons:

- In case of oral surgery, including dental extraction, or shedding of a deciduous tooth, to allow healing of the oral cavity
- Inflammatory conditions in the oral cavity
- Upper airway viral infection
- Other reasons if deemed necessary by the investigator

Interruptions should be kept to a minimum. If IMP is interrupted for more than 7 days in a row, the subject should contact the investigator before restarting the treatment.

If IMP treatment is permanently discontinued, the subject is encouraged to continue in the trial for safety assessments, see also Section 5.1.

The sponsor should be notified in case of IMP discontinuation due to an AE.

9.5 IMP compliance

Subjects and/or their parent/caregiver must be instructed to bring all residual and unused IMP and all empty packaging to the site at every visit. Compliance will be assessed at each visit by SLIT-tablets counts. If IMP compliance is less than 80% or more than 100%, the investigator should discuss the reason and educate the subject to comply with the protocol.

The site staff should in cooperation with the subject aim at keeping the IMP compliance between 80-100%.

9.6 Post-trial treatment

After the end of the trial, the investigator must advise trial subjects on access to appropriate and available treatment. Such treatment will not be sponsored by ALK.

10 Visit schedule

In Table 5, the assessments that should be performed at each visit is described. The assessments are listed in the chronological order in which they should preferably be performed.

Table 5 Visit schedule

Visit ID	Assessments to be performed at the visit
**Visit 1 (screening)**	• Obtain written informed consent for the trial, for storage of serum samples in the ALK Research Biobank (Canada and Poland only), and for pharmacogenetic biobank. Subject assent must be obtained if applicable. Written informed consent and subject assent must be obtained before any other trial procedures are performed<br>• Obtain demographic data (month/year of birth, age, race, ethnic origin, sex)<br>• Record medical history including rhinoconjunctivitis and asthma medication history<br>• Assess symptoms of eosinophilic oesophagitis<br>• Record use of relevant concomitant and previous medication<br>• Measure height and weight<br>• Measure vital signs<br>• Perform physical examination<br>• Collect urine and perform pregnancy test if applicable<br>• Perform SPT[24] and evaluate results[25] (to be performed at visit 2, if washout of concomitant medication is required)<br>• Assess compliance with inclusion and exclusion criteria<br>• Assess AEs<br>• Schedule date for visit 2<br>• Collect blood and urine sample for safety laboratory assessments<br>• Collect blood sample for specific IgE against *D. pteronyssinus* and *D. farinae*<br><br>**For Canada and Poland:**<br>• Collect blood sample for immunological assessments<br>• Collect blood samples for ALK Research Biobank (should only be performed if the specific consent has been obtained)
**Visit 2 (Baseline)**	• Assess AEs occurring since the last visit<br>• Assess symptoms of eosinophilic oesophagitis

[24] No data are available for SPT in pregnant subjects, therefore perform the urine pregnancy test before SPT if applicable
[25] If medication that could interfere with the SPT, according to Table 8, has not been washed out and the positive control is <3 mm for subjects in Europe or <5 mm for subjects in North America, the SPT must be repeated after the interfering medication has been washed out.

Visit ID	Assessments to be performed at the visit
	• Measure height and weight<br>• Record changes to concomitant medication<br>• Re-assess compliance with inclusion and exclusion criteria<br>• Perform oropharyngeal examination<br>• Measure vital signs<br>• Collect urine and perform pregnancy test if applicable<br>• SPT, if not possible at visit 1 due to necessary washout of concomitant medication<br>• Instruct the subject and their parent/caregiver in the use of rhinoconjunctivitis rescue medication<br>• Instruct the subject and their parent/caregiver in the use of asthma rescue medication if applicable<br>• Dispense rhinoconjunctivitis rescue medication<br>• Dispense asthma rescue medication if applicable<br>• Show trial video for symptom rating<br>• Issue, activate and instruct the subject and their parent/caregiver in the use of the eDiary for the next 3 weeks<br>• Schedule date for visit 3
**Visit 3 (Randomisation)**	• Assess AEs occurring since the last visit<br>• Assess symptoms of eosinophilic oesophagitis<br>• Record changes to concomitant medication<br>• Check eDiary compliance<br>• Re-assess compliance with inclusion and exclusion criteria<br>• Measure height and weight<br>• Measure vital signs and body temperature<br>• Measure FEV_1 and calculate the % of predicted FEV_1 after at least 6 hours of SABA washout<br>• Collect urine and perform pregnancy test if applicable<br>• Randomise the subject<br>• Collect used rescue medication as applicable<br>• Dispense rhinoconjunctivitis and asthma rescue medication as needed<br>• Instruct the subject and their parent/caregiver in the use of rhinoconjunctivitis rescue medication<br>• Instruct the subject and their parent/caregiver in the use of asthma rescue medication if applicable<br>• Issue and instruct the subject and their parent/caregiver in the use of the local and systemic allergic reaction emergency plan<br>• Dispense rescue medication for severe allergic reaction

Visit ID	Assessments to be performed at the visit
	(adrenaline/epinephrine auto-injector) in countries where this is a regulatory requirement<br>• Instruct the subject and their parent/caregiver in the use of rescue medication for severe allergic reaction<br>• Perform PRQLQ interview<br>• Evaluate subjects' absence from school/ work due to subjects' allergy and/or asthma<br>• Evaluate parent/caregivers' absence from work due to subjects' allergy and/or asthma<br>• Evaluate health care utilisation due to subjects' allergy and/or asthma<br>• Perform oropharyngeal examination before and 30 ±5 minutes after IMP intake[26]<br>• Instruct the subject and their parent/caregiver on how to use the IMP<br>• First intake of IMP at the clinic. The first dose will be administered at the clinic with the subject remaining in the clinic for 30 minutes after IMP intake for recording of solicited AEs[27]<br>• Dispense IMP to the subject<br>• Schedule V3, TC1 and TC2 dates in eDiary and instruct the parent/caregiver recording of pre-specified symptoms in eDiary the following 27 days<br>• Schedule date for visit 4
**Visit 4 (Solicited AEs)**	• Assess AEs occurring since the last visit including solicited AEs<br>• Assess symptoms of eosinophilic oesophagitis<br>• Perform oropharyngeal examination<br>• Record changes to concomitant medication<br>• Measure height and weight. If necessary, adjust the local and systemic allergic reaction emergency plan<br>• Measure vital signs<br>• Collect urine for pregnancy test if applicable<br>• Check eDiary compliance and return eDiary to parent/caregiver for use after TC1 and TC2<br>• Collect used IMP, rescue medication as applicable and perform drug accountability and IMP compliance check<br>• Dispense IMP to the subject

[26] For subjects with severe oral inflammation (e.g. oral lichen planus, mouth ulcers or thrush), oral wounds or following oral surgery, including dental extraction, or following tooth loss, initiation of IMP treatment should be postponed until the oral cavity has healed, see Section 9.2.

[27] For subjects with symptoms of, or in treatment for, asthma exacerbation, upper respiratory tract infection, acute sinusitis, acute otitis media or other relevant infections, initiation of IMP treatment should be postponed until the condition has improved, see Section 9.2.

Visit ID	Assessments to be performed at the visit
	• Dispense rhinoconjunctivitis and asthma rescue medication as needed<br>• Dispense rescue medication for severe allergic reaction (adrenaline/epinephrine), if needed<br>• Evaluate subjects' absence from school/ work due to subjects' allergy and/or asthma<br>• Evaluate parent/caregivers' absence from work due to subjects' allergy and/or asthma<br>• Evaluate health care utilisation due to subjects' allergy and/or asthma<br>• Schedule date for visit 5
**TC1**	• Assess AEs occurring since the last visit<br>• Assess symptoms of eosinophilic oesophagitis<br>• Record changes to concomitant medication<br>• Evaluate subjects' absence from school/ work due to subjects' allergy and/or asthma<br>• Evaluate parent/caregivers' absence from work due to subjects' allergy and/or asthma<br>• Evaluate health care utilisation due to subjects' allergy and/or asthma<br>• Instruct the parent/caregiver in activating the eDiary for the next 14 days following the TC
**TC2**	• Assess AEs occurring since the last visit<br>• Assess symptoms of eosinophilic oesophagitis<br>• Record changes to concomitant medication<br>• Evaluate subjects' absence from school/ work due to subjects' allergy and/or asthma<br>• Evaluate parent/caregivers' absence from work due to subjects' allergy and/or asthma<br>• Evaluate health care utilisation due to subjects' allergy and/or asthma<br>• Instruct the parent/caregiver in activating the eDiary for the next 14 days following the TC
**Visit 5**	• Assess AEs occurring since the last visit<br>• Assess symptoms of eosinophilic oesophagitis<br>• Record changes to concomitant medication<br>• Perform oropharyngeal examination<br>• Measure height and weight. If necessary adjust the local and systemic allergic reaction emergency plan<br>• Measure vital signs<br>• Collect urine for pregnancy test if applicable

Visit ID	Assessments to be performed at the visit
	• Collect used IMP, rescue medication as applicable and perform drug accountability and IMP compliance check<br>• Dispense IMP to the subject<br>• Dispense rhinoconjunctivitis and asthma rescue medication, as needed<br>• Dispense rescue medication for severe allergic reaction (adrenaline/epinephrine auto-injector), if needed<br>• Perform PRQLQ interview<br>• Evaluate subjects' absence from school/ work due to subjects' allergy and/or asthma<br>• Evaluate parent/caregivers' absence from work due to subjects' allergy and/or asthma<br>• Evaluate health care utilisation due to subjects' allergy and/or asthma<br>• Schedule date for visit 6
**Visit 6**	• Assess AEs occurring since the last visit<br>• Assess symptoms of eosinophilic oesophagitis<br>• Record changes to concomitant medication<br>• Perform oropharyngeal examination<br>• Measure height and weight. If necessary adjust the local and systemic allergic reaction emergency plan<br>• Measure vital signs<br>• Collect urine for pregnancy test if applicable<br>• Collect used IMP, rescue medication as applicable and perform drug accountability and IMP compliance check<br>• Dispense IMP to the subject<br>• Dispense rhinoconjunctivitis and asthma rescue medication, as needed<br>• Show trial video for symptom rating<br>• Dispense rescue medication for severe allergic reaction (adrenaline/epinephrine auto-injector), if needed<br>• Perform PRQLQ interview<br>• Evaluate subjects' absence from school/ work due to subjects' allergy and/or asthma<br>• Evaluate parent/caregivers' absence from work due to subjects' allergy and/or asthma<br>• Evaluate health care utilisation due to subjects' allergy and/or asthma<br>• Instruct the parent/caregiver in completing the eDiary for the next 8 weeks following the visit<br>• Schedule date for visit 7

Visit ID	Assessments to be performed at the visit
**Visit 7 (End of trial)**	• Assess AEs occurring since the last visit<br>• Assess symptoms of eosinophilic oesophagitis<br>• Record changes to concomitant medication<br>• Measure height and weight<br>• Measure vital signs<br>• Measure FEV_1 and calculate the % of predicted FEV_1 after at least 6 hours of SABA washout<br>• Perform physical examination<br>• Collect unused and partly used IMP/rescue medication and empty blistercards<br>• Collect unused adrenaline/epinephrine auto-injector, if applicable<br>• Perform drug accountability<br>• Check IMP compliance<br>• Collect eDiary<br>• Check eDiary compliance<br>• Perform PRQLQ interview<br>• Evaluate subjects' absence from school/ work due to subjects' allergy and/or asthma<br>• Evaluate parent/caregivers' absence from work due to subjects' allergy and/or asthma<br>• Evaluate health care utilisation due to subjects' allergy and/or asthma<br>• Schedule date for a telephone follow-up contact approximately two weeks later<br>• Collect urine for pregnancy test, if applicable, and for safety laboratory assessments<br>• Collect blood sample for safety laboratory assessments<br>• Collect blood sample for pharmacogenetic biobank (should only be performed if the specific consent has been obtained)<br><br>**For Canada and Poland:**<br>• Collect blood sample for immunological assessments<br>• Collect blood, sample for ALK Research Biobank (should only be performed if the specific consent has been obtained)
**Follow-up phone contact**	• Record AEs.<br>If an AE was ongoing at the previous visit, if a new AE is identified at the telephone contact, or if one of the safety laboratory parameters measured at the previous visit showed a clinically significant abnormality, the subject could be asked to return to the trial site<br>• Assess symptoms of eosinophilic oesophagitis

Visit ID	Assessments to be performed at the visit
	• Record changes to concomitant medication
**Unscheduled visit**	• Assess AEs occurring since the last visit<br>• Record changes to concomitant medication<br>• Measure height and weight. If necessary adjust the local and systemic allergic reaction emergency plan<br>The following procedures will be performed if deemed necessary by the investigator or designee:<br>• Measure vital signs<br>• Physical examination<br>• Assess symptoms of eosinophilic oesophagitis<br>• Perform oropharyngeal examination<br>• Measure FEV_1 and calculate the % of predicted FEV_1<br>• Collect urine for pregnancy test if applicable<br>• Collect blood and/or urine sample for safety laboratory assessments<br>• Collect blood sample for specific IgE against *D. pteronyssinus and D. farinae*<br>• Dispense IMP<br>• Intake of IMP at clinic<br>• Dispense rhinoconjunctivitis and asthma rescue medication<br>• Dispense rescue medication for severe allergic reaction (adrenaline/epinephrine auto-injector)<br>• Evaluate subjects' absence from school/ work due to subjects' allergy and/or asthma<br>• Evaluate parent/caregivers' absence from work due to subjects' allergy and/or asthma<br>• Evaluate health care utilisation due to subjects' allergy and/or asthma<br>• Instruct the parent/caregiver in activating the eDiary for the next 3 weeks following the visit

11 Assessments

This section outlines the trial procedures that will be performed during the trial. For further details on the specific timing of the procedures please refer to the visit schedule in section 10.

The tasks listed below must be performed by a physician or other medically trained person (according to national legislation):

- Obtainment of informed consent
- Evaluation of in- and exclusion criteria
- Physical examination

- Assessment of AEs/SAEs
- Assessment of FEV_1 and laboratory results
- Decision to break the randomisation code for individual subjects

11.1 Informed consent

All parents/guardian must provide informed consent in accordance with the origins of the Declaration of Helsinki (**World Medical Association 2013**) and the applicable laws of the country. The written informed consent must be obtained before any trial activities are performed, including any period for washout of concomitant medication.

If the minor can understand the risks and benefits of the trial, he/she should also be informed. Assent should be obtained according to national requirements.

It is the responsibility of the principal investigator or a sub-investigator to obtain the written informed consent/assent from the parents/guardian and subject, respectively.

The investigator must explain the nature of the trial, its purpose, the procedures involved, the expected duration, the potential risks and benefits involved and any discomfort it may entail and provide the subject with a copy of the information sheet and the consent form. Information to the parent/guardian and subject can be delegated to a nurse, but the investigator must be available for questions and both the nurse and the investigator must sign the consent form to document this.

The parent/guardian and subject must be given sufficient time to consider the trial before deciding whether to participate. Each subject must be informed that participation in the trial is voluntary, that he/she may discontinue from the trial at any time and that withdrawal of consent/assent will not affect his/her subsequent medical treatment or relationship with the treating physician.

The informed consent/assent form must be signed and dated before the subject enters the trial (i.e. before any trial related activity). The investigator must give a copy of the signed informed consent/assent to the parent/guardian and subject. The investigator should keep the original.

If information becomes available that may be relevant to the subject’s willingness to continued participation in the trial the subject information sheet will be updated by the sponsor and approved by an IEC/IRB and the regulatory authorities. The parent/guardian and subject must be informed in a timely manner about the updated subject information sheet and written informed consent/assent must be obtained.

11.2 Consent for long-term storage of biobank samples for Canada and Poland

When the subject/parent/guardian in Canada and Poland is asked to consent to the participation in the trial, children with a body weight over 20 kg will be asked specifically if they will donate 2 blood samples of 5 ml each.

All samples will be stored in the ALK Research Biobank. The answer to this question will be recorded on the consent form for retention of blood samples for future research, as well as in the eCRF. The samples will be taken at visits 1 and 7. The parent/guardian cannot consent to sample donation after visit 3 as the first sample has to be taken before first IMP intake. Participation in the trial does not depend on giving consent to donating biobank samples.

Assent from the subject should be obtained according to national requirements. If sampling and storage is not accepted by the subject/parent/guardian, the samples must not be drawn.

11.3 Consent for collection of blood samples for pharmacogenetic biobank

When the subject/parent/guardian are asked to consent/assent to the participation in the trial, children with a body weight over 20 kg will be asked specifically if they can accept sampling for pharmacogenetic biobank and storage of a DNA and RNA sample. The answer to this question will be recorded on the consent form for retention of samples for future pharmacogenetic testing, as well as in the eCRF. The sample volume will be 2.5 ml and the samples will be taken at visit 7. Participation in the trial does not depend on giving consent to donating pharmacogenetic samples. Assent from the subject should be obtained according to national requirements. If pharmacogenetic sampling and storage is not accepted by the subject/parent/guardian, the samples must not be drawn.

11.4 Demographics

The following data will be recorded:

- Month and year of birth[28]
- Age
- Race and ethnic origin
- Sex

11.5 Medical history

The relevant medical history, incl. diseases present at trial entry must be recorded in the eCRF. This includes a detailed allergy history including recording of the subject's history of rhinitis, conjunctivitis, asthma, atopic dermatitis and food allergy.

Those subjects suspected of having a history of asthma must meet at least one of the following 4 criteria to be considered as having a diagnosis of asthma (and documented as such in the eCRF):

- At least one episode of wheeze, cough, shortness of breath or chest tightness and a change in FEV_1 ≥12% after beta2-agonist administration
- Recurrent wheeze with or without prolonged phase of forced exhalation observed at physical examination and an intake of asthma medication which resulted in a clinically relevant effect
- Wheezing with or without prolonged phase of forced exhalation observed at physical examination and a change in FEV_1 ≥12% after beta2-agonist administration
- Using asthma medication

---

[28] Only year of birth in countries which do not allow the month to be collected

11.6 Concomitant and previous medication

The subject's use of all concomitant medication including rhinoconjunctivitis and asthma medication must be recorded. Relevant previous medication should also be recorded. This includes previous rhinoconjunctivitis and asthma medication within the past 1 year. Other previous medication taken by the subject should also be recorded if considered relevant by the investigator or if discontinued within 1 year from visit 1. Standard information about the medication will be collected including name of medication, dose, administration route and treatment period.

Medication not provided as a part of this trial should be kept to a minimum during the trial. However, if considered necessary for the subject's well-being, concomitant medication may be given at the discretion of the investigator according to the local standard of care.

At each visit the investigator should ask the subject about use of concomitant medication. All concomitant medication must be documented in the subject's medical records and in the eCRF (generic name). Furthermore, each change in concomitant medication (e.g. new treatment, discontinuation of treatment and change in dosage/routine) during the trial must be documented in the same way.

11.7 Height and weight

The subject's body height and weight will be recorded at all visits as detailed in the flow chart, Table 1.

11.8 Vital signs

Vital signs will include measurement of BP, heart rate in a seated position (after ≥ 3 minutes of seated inactivity) and respiratory rate (breaths/minute) measured while in a seated position after ≥3 minutes of seated inactivity (counted over 30sec and multiplied by 2). Significant findings that are present at screening must be recorded as medical history in the eCRF. Significant findings found at the following visits, which meet the definition of an AE, must be recorded on an AE page in the eCRF.

11.9 Lung function

The assessment of the lung function will include measurements of forced vital capacity (FVC), and FVC percent predicted, FEV_1 and FEV_1 percent predicted, for all subjects. Lung function measurements will be performed with a spirometer available at the clinic. FVC and the derived FEV_1 is measured as 3 valid measurements and the highest value will be entered in the eCRF. Lung function will be measured according to the American Thoracic Society (ATS)/European Respiratory Society (ERS) spirometry recommendations (**Reddel et al. 2009**). The spirometry values will be reported by the site according to the site's pre-programmed spirometric reference equations. The reference equations and values used should be documented on every pulmonary function report. For subjects with asthma, lung function will be measured with subjects on their usual asthma medication following at least a 6-hour washout of SABA.

11.10 Physical examination

The physical examination should be performed by a physician or designee in accordance with local practice and should be based on the following body systems see Table 6.

Table 6 Physical examination

Body system	Minimum examinations to be completed
General appearance	Nutritional status, consciousness, skin colour and developmental status
Skin	Inspection of skin
Head	Ears - Inspection of auricles and external canal (otoscopy is not required)<br>Eyes - Inspection of conjunctivae and eyelids<br>Nose - Inspection of nasal mucosa (nasal endoscopy is optional)<br>Oral cavity - oropharyngeal inspection of lips, tongue, tonsils and uvula will be performed for signs of mouth irritation, edema, and any other abnormalities. When performed at visit 3, it will take place before and 30 minutes after IMP administration.
Lymph nodes	Examination of lymph nodes (cervical, axillary, and inguinal lymph nodes)
Respiratory	Assessment of respiratory effort, including respiratory rate<br>Palpation and percussion of chest<br>Auscultation/stethoscopy of lungs
Heart	Auscultation/stethoscopy of the heart
Abdomen	It is up to the investigators discretion to evaluate whether an examination of the abdomen is necessary. Questions regarding symptoms may be sufficient.
Urogenital	It is up to the investigators discretion to evaluate whether an examination of the urogenital system is necessary. Questions regarding symptoms may be sufficient.
Musculoskeletal and neurological	It is up to the investigators discretion to evaluate whether an examination of the musculoskeletal/neurological system is necessary. Questions regarding symptoms may be sufficient.
Other abnormality	If applicable

Physical examination of optional body systems not performed should be marked as not done (ND) in the eCRF.

Significant findings that are present at screening must be recorded as medical history in the eCRF. Significant findings found at the following visits, which meet the definition of an AE, must be recorded on an AE page in the eCRF.

11.11 Pregnancy test

For female subjects of childbearing potential, a urine pregnancy test will be performed at all regular visits. For female subjects who have their first menstrual period during the trial, a urine pregnancy test will be performed at all subsequent visits. The urine pregnancy test will be performed by dip-stick at the trial site.

11.12 Skin prick test

All SPT materials will be supplied by the sponsor. The SPT must be performed according to the procedure provided by the sponsor with wheal sizes entered into the eCRF.

No data is available for SPT in pregnant subjects so the urine pregnancy test must be performed before the SPT.

Subjects enrolled in the trial will be tested for the allergens listed in Table 7.

Table 7 Skin prick test

Country	Allergen
All Countries	Positive control – Histamine<br>Negative control – Saline<br>HDM – *Dermatophagoides pteronyssinus*<br>HDM – *Dermatophagoides farinae*<br>Cat – *Felis domesticus*<br>Dog – *Canis familiaris*<br>Mould – *Alternaria alternata*<br>Mould - *Cladosporium*<br>Grass – *Phleum pratense*<br>Ragweed – *Ambrosia artemisiifolia*<br>Birch – *Betula verrucosa*
Canada and United States	Oak - *Quercus alba*

Some medication may affect the outcome of the SPT and should be washed out before performing the SPT (see Table 8).

Table 8 Medication with a possible interference with SPT

Drug[29]	Recommended washout period prior to performing SPT
Antihistamine<br>• Oral, intravenous or topical (skin)<br>• Long-acting (astemizole)	 3 days<br>100 days
Tricyclic antidepressant medications and antidepressant medication with antihistaminic effects (e.g. doxapine, mianserine)	14 days
Antipsychotic medications with antihistaminic effects (e.g. chlorpromazine, levomepromazine, clozapine, olanzapine, tioridazine)	7 days
Glucocorticosteroid<br>• Local application of ultra high and high potency[30] (on the skin area used for SPT)<br>• Oral<br>• Short-acting parenteral<br>• Long-acting parenteral (intra-articular or intramuscular)	 21 days<br>30 days<br>30 days<br>90 days
Pizotifene	7 days

11.13 Blood and urine sampling

The following blood samples will be drawn during the trial:

Purpose	Volume	Number of samples
Safety / Hematology	2 ml	2
Safety / Blood chemistry	2.5 ml	2
Screening / IgE	5 ml	1
For Canada and Poland:<br>Immunology	5 ml	2
**Total**<br>**Canada and Poland:**<br>**Remaining countries:**	 **24 ml**<br>**14 ml**	 **7**<br>**5**

[29] If medication that could interfere with the SPT, according to Table 8, has not been washed out, the SPT must be repeated after the interfering medication has been washed out.
[30] Augmented betamethasone dipropionate 0.05%; clobetasol propionate 0.05%; diflorasone diacetate 0.05%; fluocinonide 0.1%; flurandrenolide 4 mcg per m2; halobetasol propionate 0.05%; amcinonide 0.1%; betamethasone dipropionate 0.05%; desoximetasone; halcinonide 0.1%

The following urine samples will be collected:

Purpose	Volume	Number of samples
Safety / Urinalysis	~10 ml	2
Pregnancy tests	NA	At each visit (if applicable)

The following blood samples will only be drawn if specific consent has been obtained:

Purpose	Volume	Number of samples during the trial
For Canada and Poland:<br>Future research - serology sample (biobank samples)	5 ml	2
Long term storage - DNA + RNA sample (pharmacogenetic biobank)	2.5 ml	1
**Total Blood**<br>**Canada and Poland:**<br>**Remaining countries:**	 **12.5 ml**<br>**2,5 ml**	 **3**<br>**1**

11.14 Laboratory assessments

All laboratory assessments will be performed centrally at a certified laboratory selected by the sponsor with the exception of urine dipsticks, which will be handled on site.

The clinical laboratory values will be reported to the investigator by the laboratory and he/she must immediately review them for significance. The laboratory results from the initial visits must be available before randomisation.

Laboratory procedures are described in a separate laboratory manual that also details blood sampling and shipment procedures.

Blood samples should be taken using standard venepuncture techniques. The planned total volume of blood drawn from each subject is 24 ml for Canada and Poland and 14 ml for remaining countries. Additional 10 ml will be collected for subjects who consent to the biobank samples in Canada and Poland and 2.5 ml for the pharmacogenetic sample. Local anaesthesia may be used in connection with blood sampling.

Urine will be collected for urinalysis. Urine dipsticks to be used on site will be provided by the central laboratory. A microscopic examination should be performed only if any of the urine evaluations are abnormal.

The following laboratory variables will be measured:

<u>Hematology:</u>

Erythrocytes, haemoglobin, haematocrit, mean corpuscular volume, mean corpuscular haemoglobin, platelets, leukocytes, neutrophils, eosinophils, basophils, lymphocytes, and monocytes.

Blood chemistry:

Creatinine, urea, sodium, potassium, chloride, calcium, glucose, albumin, total bilirubin, alkaline phosphatase, aspartate aminotransferase / serum glutamic-oxaloacetic transaminase, alanine aminotransferase / serum glutamic pyruvic transaminase and lactate dehydrogenase.

Urinalysis:

Protein, pH, glucose, ketone, leukocytes, urobilinogen, bilirubin, haemoglobin nitrite and specific gravity.

IgE:

To support the diagnosis of allergy against HDM, blood samples will be drawn at the screening visit for determination of specific IgE against *D. pteronyssinus and D. farinae*.

These samples will be analysed together with the samples for the safety laboratory assessments (see above), and the results will be reported to sites for assessment of subject eligibility.

Immunology for Canada and Poland:

To assess the immunological response of the treatment, blood samples will be drawn for determination of antigen-specific antibodies (e.g. IgE and IgG_4), total IgE and other immunological parameters at visits 1 and 7. At each time point, 5 mL blood will be drawn. The samples will be analysed by ALK Laboratories.

Biobank blood sample for Canada and Poland:

This blood sample will only be drawn if the subject has a body weight of more than 20 kg and consents to long term storage of an immunology sample. If blood sampling for the biobank is accepted, two 5 mL blood samples will be drawn. The blood sample will be drawn at visits 1 and 7 where blood sampling already is planned. The subject will consequently only donate extra blood, no additional venepuncture is required.

The purpose of the biobank blood samples is to continue the research into the immunological processes involved in the observed clinical effects in subjects treated with allergy immunotherapy, and which today is not fully understood. One of the goals of this research is to identify one or more surrogate markers, which can predict clinical efficacy in the individual subject, i.e. which can help to ensure optimal treatment for future subjects with allergy. The surrogate markers may be antibody levels, cytokine profiles, cell surface markers, specific set of proteins or metabolites, combinations hereof, etc. Although the exploratory biomarker analyses will help to increase our understanding, the efforts described in this protocol are strictly research based. Thus, as the complex interactions between allergy immunotherapy, rhinoconjunctivitis and asthma are currently not characterised to a level that translates to a meaningful clinical advantage, individual results from the exploratory biomarker analyses will not be given to the subjects. For the same reasons, individual results will not be added to the subjects' medical records.

The subjects will have no direct benefit from the exploratory biomarker analyses.

If the sponsor publishes results obtained from genotyping or biomarker studies based on pharmacogenetic samples obtained in the trial, the results will be published in such a way that it cannot be tied to an individual subject.

Blood sample for pharmacogenetic biobank:

This blood sample will only be drawn if the subject has a body weight of more than 20 kg and consents to long term storage of a DNA sample.

DNA and RNA material will be extracted. If pharmacogenetic sampling is accepted, a 2.5 mL blood samples will be drawn at visit 7 where blood sampling already is planned. The subject will consequently only donate an extra blood sample, no additional venepuncture is required.

The DNA and RNA samples collected in the current trial will be used to investigate various genetic causes for how subjects may respond to the treatment as well as the impact the treatment may have on the epigenetic profile of the subjects. The DNA and RNA samples will be stored to provide a resource for future studies conducted by ALK focused on the investigation of how genes can affect drug absorption, distribution and removal from the body, and drug action in the body or vice versa, how drugs can affect gene expression profiles.

Studies may include analyses for identifying for instance genomic markers of atopic diseases, efficacy of allergy treatment, AEs, or other genomic markers relevant for the atopic disease and treatment of allergy. Pharmacogenetic results may be compared to pharmacodynamic results or clinical outcomes. Any significant pharmacogenetic relationships to outcome will require validation in future clinical trials.

Since the complex interactions between allergy immunotherapy, rhinoconjunctivitis and asthma are currently not characterised to a level that translates to a meaningful clinical advantage, individual results from the pharmacogenetic analyses will not be given to the subjects. For the same reasons, individual results will not be added to the subjects' medical records.

The subjects will have no direct benefit from the exploratory biomarker analyses.

If ALK publishes results obtained from genotyping or biomarker studies based on pharmacogenetic samples obtained in the trial, the results will be published in such a way that it cannot be tied to an individual subject.

11.15 Adverse event assessment

The investigator will question the subject about AEs since the last visit (as applicable), and will record the information on the eCRF (see Section 12 for instructions on assessment and reporting of (S)AEs and pregnancies to the sponsor).

The eDiary will be used to capture information on 15 pre-specified symptoms/signs identified as potential local side effects of sublingual immunotherapy on a daily basis from the subject for the first 28 days of treatment (**Passalacqua et al. 2013**).The eDiary is to be completed by the parent/caregiver together with the subject. The parent/caregiver will be trained by the investigator or designee at visit 3 on the proper method to complete the eDiary. The reported symptoms will be evaluated by investigator and reported in the eCRF as solicited AEs.

The solicited AEs that occur on Day 1 within 30 minutes of IMP intake, including the time of AE start and AE stop will be monitored by site personnel, recorded in source documents, and entered into the eCRF. For details on solicited AEs, please see section 11.19.

11.16 Assessment of rhinoconjunctivitis and asthma symptoms

Rhinoconjunctivitis and asthma symptoms will be recorded by the parent/ caregiver together with the subject in the eDiary at specified periods during the trial. A video with information on the rating will be available for the subjects.
A total of 10 symptoms; 6 rhinitis and/or conjunctivitis symptoms and 4 asthma symptoms will be measured on a scale from no symptoms to severe symptoms (for details, please see Table 9 and Table 10).

The subject should be instructed in the symptom score using the following definitions:

Table 9 Subject's symptom scoring

Scored by subject	Definition of score	Numerical Score[1]
No symptoms	No sign/symptom evident	0
Mild symptoms	Symptom clearly present, but minimal awareness; easily tolerated	1
Moderate symptoms	Definite awareness of symptom that is bothersome but tolerable	2
Severe symptoms	Symptoms that are hard to tolerate; causes interference with activities of daily living and/or sleeping	3

[1] Scoring scales are not seen by the subjects

Table 10 Construction of symptom scores[1]

	Rhinitis DSS	Conjunctivitis DSS	Rhinoconjunctivitis DSS	Asthma DSS
**Rhinitis symptoms**				
Runny nose	0-3		0-3	
Blocked nose	0-3		0-3	
Sneezing	0-3		0-3	
Itchy nose	0-3		0-3	
**Conjunctivitis symptoms**				
Gritty feeling/red/itchy eyes		0-3	0-3	
Watery eyes		0-3	0-3	
**Asthma symptoms**				
Chest tightness				0-3
Wheezing				0-3
Cough				0-3
Shortness of breath				0-3
**Total range**	**0-12**	**0-6**	**0-18**	**0-12**

[1] Scoring scales are not seen by the subjects

For the rhinitis CSMS and the rhinoconjunctivitis CSMS recommended by the EAACI (Pfaar et al. 2014), a rhinitis EAACI symptom score and a rhinoconjunctivitis EAACI symptom score are constructed as the sum of each individual daily symptom score divided by the number of symptoms (i.e. rhinitis EAACI symptom score total range 0-3; rhinoconjunctivitis EAACI symptom score total range 0-3).

11.17 Medication assessment

All subjects are provided with open-label rescue medication for treatment of rhinoconjunctivitis. Use of rhinoconjunctivitis and asthma rescue medication will be recorded by the parent/caregiver together with the subject on a daily basis during eDiary periods. Parents/caregivers are instructed to report the subject's use of specific rhinoconjunctivitis and asthma rescue medication via the daily eDiary during the baseline period (period 2) and at specified timepoints during the trial. To transform the amount of rhinoconjunctivitis rescue medication used into medication scores, the scoring principles detailed in Table 11 will be used.

Table 11 Scoring of rhinoconjunctivitis rescue medication

Rescue medication	Subject dosing	Score/ Dose unit[1]	Maximum daily score
**Rhinitis medication score**			
Desloratadine oral solution, 0.5 mg/ml	5 years old: 2.5 ml (1.25 mg) once daily	4	4
	6-11 years old: 5 ml (2.5 mg) once daily		
	≥12 years old: 10 ml (5 mg) once daily		
Loratadine tablets[2], 10 mg	6-12 years old and > 30 kg: 1 tablet (10 mg) once daily		
	>12 years old: 1 tablet (10 mg) once daily		
Mometasone furoate nasal spray, 50 micrograms/dose	<12 years old: 1 puff in each nostril once daily	4	8
	≥12 years: 2 puffs in each nostril once daily	2	
**Maximum daily rhinitis medication score[3]**			12
**Conjunctivitis medication score**			
Desloratadine oral solution 0.5 mg/ml	5 years old: 2.5 ml (1.25 mg) once daily	2	2
	6-11 years old: 5 ml (2.5 mg) once daily		
	≥12 years old: 10 ml (5 mg) once daily		
Loratadine tablets[2], 10 mg	6-12 years old and > 30 kg: 1 tablet (10 mg) once daily		
	>12 years old: 1 tablet (10 mg) once daily		
Olopatadine eye drops, 1 mg/ml	1 drop in each eye twice daily	1.5	6
**Maximum daily conjunctivitis medication score[3]**			8
**Maximum daily rhinoconjunctivitis medication score[3]**			20

[1] Scoring scales are not seen by the subjects
[2] Loratadine will count 4 in the rhinitis score and 2 in the conjunctivitis score, based on assumed equal efficacy of antihistamine on the 4 nasal symptoms and 2 eye symptoms (Salmun & Lorber 2002)
[3] If any subject exceed the recommended daily dose of symptomatic medication, the maximum daily score will be used.

For the CSMS recommended by the EAACI (Pfaar et al. 2014), the rhinoconjunctivitis rescue medication use reported by subject's parent/caregiver in the eDiary will be transformed into a rhinoconjunctivitis EAACI medication score using the stepwise scoring principles detailed in Table 12 (EAACI medication score total range 0-2).

Table 12 Scoring of rhinoconjunctivitis medication for the CSMS (Pfaar et al. 2014)

Rescue medication	EAACI medication score
Desloratadine oral solution, 0.5 mg/ml	1
Loratadine tablets, 10 mg	1
Olopatadine eye drops, 1 mg/ml	1
Mometasone furoate nasal spray, 50 micrograms/dose	2
The EAACI medication score is defined as the maximum value of either of the 4 above components (i.e. EAACI medication score total range 0-2). Note that in (Pfaar et al. 2014) the EAACI medication score may also assume a value of 3 (oral corticosteroids). This is not possible in the present trial since oral corticosteroids are not included in the provided rescue medication.	

11.18 Local and systemic allergic reaction emergency plan

The subject and parent/caregiver will be provided with educational information regarding symptoms of anaphylaxis and treatment, including a written local and systemic allergic reaction emergency plan (Epstein et al. 2017).

The local and systemic allergic reaction emergency plan will detail the mild to moderate local reactions that include:

- Mouth: bothersome itching, and/or mild swelling of lips and/or tongue
- Throat: bothersome itching, irritation, and/or mild tightness
- Ear: bothersome itching
- Gastrointestinal: mild abdominal pain, nausea, and/or cramps

The local and systemic allergic reaction emergency plan specifies that these symptoms may be treated with an antihistamine, if treatment is required, where the dose is pre-specified by the investigator on the plan.

Severe reactions are defined as:

- Local reaction with swelling in the mouth/throat causing hoarseness and/or throat closing
- Systemic reactions such as
  - Skin: hives all over body and/or redness all over body
  - Lung: shortness of breath, cough, and/or wheezing
  - Heart: weak pulse, dizziness, and/or passing out
  - Gastrointestinal: severe abdominal pain, vomiting, diarrhea, and/or cramping

Severe reactions should be treated in accordance with the local and systemic allergic reaction emergency plan as instructed by the investigator.

11.19 Solicited AEs

The eDiary will be used to capture 15 pre-specified symptoms/signs, identified as potential local side effects of sublingual immunotherapy (Passalacqua et al. 2013):

- Food tastes different

- Mouth ulcer[31]
- Swelling in the back of the mouth
- Itching in the mouth
- Itching in the ear
- Swelling of the lips
- Swelling of the tongue
- Tongue pain
- Tongue ulcer[32]
- Throat irritation/tickle
- Throat swelling
- Stomach pain
- Nausea (feel like throwing up)
- Vomiting
- Diarrhea

If the subject answers “yes” to any of the symptoms, the subject will be asked if any medication was used to treat the symptom.

The reported symptoms will be evaluated by investigator and reported as AEs on the AE form in the eCRF at the discretion of the investigator.

The AE start and stop time of solicited local AEs that occur on Day 1 within 30 minutes of IMP intake will be monitored by site personnel, recorded in source documents, and entered into the eCRF. After Day 1, standard AE reporting conventions will be utilised.

11.20 Eosinophilic oesophagitis

At each visit to the clinic, subjects/parents/caregivers will be asked whether any of the following are present or has occurred since the last clinic visit:

- food impaction requiring medical intervention
- dysphagia/difficulty swallowing requiring the subject to drink large quantities of water to swallow food
- choking or gagging with meals
- persistent (8 weeks or more) dysphagia
- a sensation of food becoming lodged in the throat
- persistent (8 weeks or more) vomiting without evidence of infection

[31] A mouth ulcer is a discrete, painful lesion affecting the lining of the mouth where the mucosa is eroded

[32] A tongue ulcer is a discrete, painful lesion affecting an area on the tongue, where the mucosa is eroded

- persistent (8 weeks or more) early satiety
- unexplained weight loss in combination with other gastrointestinal symptoms

If a subject presents with any of the above, the investigator should consider referring the subject to a gastroenterologist for evaluation. If there is a clinical suspicion of eosinophilic oesophagitis, subjects must be referred to a gastroenterologist.

11.21 Subject reported outcomes

11.21.1 eDiary assessments

During the trial, the subject's parent/caregiver will complete an eDiary together with the subject. The eDiary is a hand-held electronic device that will be issued to the subjects at visit 2. Subjects/parents/caregivers will be instructed by the investigator on how to fill in the eDiary.

The following items will be outlined in the eDiary at pre-specified intervals during the trial:

- Rhinoconjunctivitis symptoms
- Use of rhinoconjunctivitis rescue medication
- Asthma symptoms
- Use of asthma rescue medication
- Nocturnal awakenings due to asthma requiring SABA use
- Pre-specified symptoms/signs identified as potential local effect of sublingual immunotherapy, see Section 11.19, for a list of solicited symptoms/signs and Section 12.3, for capturing solicited AEs

11.21.2 Paediatric quality of life questionnaire

The PRQLQ will be completed by an interviewer during the trial. The PRQLQ will be used for all subjects aged 5 to 11 years of age although it is only validated for children from 6 years of age.

The PRQLQ has 23 questions in 5 domains (nasal symptoms, eye symptoms, practical problems, activity limitation and other symptoms) (**Juniper et al. 1998**).

Children are asked to think about how they have been during the previous week and to respond to each of the 23 questions on a 7-point scale (from 0 = not bothered/none of the time to 6 = extremely bothered/all of the time). The overall PRQLQ score is the mean of all 23 responses and the individual domain scores are the means of the items in those domains.

The PRQLQ and details on the scoring principles are described in Appendix 5.

12 Adverse events

Information about AEs, whether reported by the subject, identified by the investigator by reviewing eDiary records, detected through physical examination, laboratory test or other means, must be collected and recorded on the AE form and followed up as appropriate. Evaluation of AEs including severity, causality, outcome and seriousness assessments must be performed by a physician.

Any AE occurring from the time the informed consent was signed by the subject and until the last follow-up phone contact must be recorded and reported on an AE form in the eCRF. This includes all AEs, even AEs occurring before the subject is administered the IMP and whether or not AEs are observed in connection with the trial procedures and/or the conduct of the trial.

12.1 Definitions

Adverse event

An AE is any untoward medical occurrence in a clinical trial subject and which does not necessarily have a causal relationship with the administered IMP.

An AE can therefore be any unfavourable and unintended sign (including e.g. an abnormal laboratory finding), symptom, or disease, whether or not considered related to the trial product.

The following events should not be recorded as AEs:

- A pre-planned procedure, e.g. a surgical intervention, unless the condition for which the procedure was planned has worsened since the informed consent form was signed.
- Pre-existing conditions documented as medical history. However, any worsening in severity or frequency of a pre-existing condition during the clinical trial period must be regarded as an AE.

Serious adverse event

A SAE is any untoward medical occurrence or effect that at any dose:

- Results in death
- Is life-threatening (this refers to an event in which the subject was at risk of death at the time of the event; it does not refer to an event that hypothetically might have caused death if it had been more severe)
- Requires inpatient hospitalisation, regardless of duration, or prolongation of existing hospitalisation
- Results in persistent or significant disability or incapacity
- Is a congenital anomaly or birth defect
- Is judged to be medically important (this refers to an event that may not be immediately life-threatening or result in death or hospitalisation, but may jeopardise the subject or may require intervention to prevent one of the other outcomes listed above)

Events of special interest (ESI)

Selected AEs (non-serious or serious) will be considered ESI. ESIs are events that are considered critical for the evaluation of the safety profile for HDM SLIT-tablet and for which additional data will be collected. Events of special interest for this trial are:

- Anaphylactic reactions, anaphylaxis[33] and/or systemic allergic reactions

[33] The definition of anaphylaxis **(Sampson et al. 2006)** includes any 1 of the following 3 criteria:

1. Acute onset of an illness (minutes to several hours) with involvement of the skin, mucosal tissue, or both (e.g., generalised hives, pruritus, or flushing, or swollen lips, togue, or uvula) AND either

- Events treated with adrenaline/epinephrine
- Severe local swelling or oedema of the mouth and/or throat
- Eosinophilic oesophagitis

Medication errors, including overdose, abuse and misuse of the IMP

The definition of an AE also covers medication errors and use of the IMP outside what is foreseen in the protocol, including misuse and abuse of the product. If doses higher than the recommended dose are taken, whether intentionally or unintentionally, the risk of AEs may increase. This includes the risk of systemic allergic reactions or severe local reactions.

- Overdose: Any cumulative dose taken in one day that exceeds the dose intended by this protocol, regardless of whether the dose has caused any AEs (reporting of overdoses must be based on reported IMP exposure and not on drug accountability procedures)
- Abuse: Persistent or sporadic, intentional excessive use which is accompanied by harmful physical or psychological effects
- Misuse: Intentional and inappropriate use

12.2 Assessments

Severity

The severity of an AE is a clinical observation assessed by the investigator using the following definitions:

- Mild: Transient symptoms, no interference with the subject’s daily activities
- Moderate: Marked symptoms, moderate interference with the subject’s daily activities
- Severe: Considerable interference with the subject’s daily activities, unacceptable

Causal relationship to IMP

The causal relationship between an AE and the IMP is assessed by the investigator using the following definitions:

---

respiratory compromise (e.g., dyspnoea, wheeze-bronchospasm, stridor, reduced PEF, hypoxemia) or reduced BP* or associated symptoms of end-organ dysfunction (e.g., hypotonia [collapse], syncope, incontinence)

2. Two or more of the following that occur rapidly after exposure to a likely allergen for the subject (minutes to several hours):
    a. Involvement of the skin-mucosal tissue (e.g., generalised hives, itch-flush, swollen lips, tongue, or uvula)
    b. Respiratory compromise (e.g., dyspnoea, wheeze-bronchospasm, stridor, reduced PEF, hypoxemia)
    c. Reduced BP* or associated symptoms (e.g., hypotonia [collapse], syncope, incontinence)
    d. Persistent gastrointestinal symptoms (e.g., crampy abdominal pain, vomiting)
3. Reduced BP after exposure to known allergen for the subject (minutes to several hours): low systolic BP* or greater than 30% decrease in systolic BP

*Low systolic BP is defined as less than (70 mmHg + [2 x age]) from 1 to 10 years, and less than 90 mmHg from 11 to 17 years.

- Possible: A reasonable possibility of a causal relationship between the event and the IMP
- Unlikely: The event is most likely caused by a different aetiology than the IMP

For SAEs assessed as unlikely related to IMP, the most likely alternative aetiology should be provided.

Outcome

The outcome of an AE is assessed by the investigator using the following definitions:

- Recovered: Fully recovered or the condition has returned to baseline
- Recovered with sequelae: As a result of the AE the subject suffered persistent disability/incapacity. If the sequela qualifies as a SAE, the AE must be reported as such
- Not recovered: The condition has not returned to baseline however, symptoms may have improved
- Fatal: Event that results in death
- Unknown: The outcome is unknown. This term should only be used when no other definition is possible e.g. the subject is lost to follow-up

12.3 Collection, recording and reporting of adverse events

At each contact with the trial site, the subject must be asked about AEs in an objective manner such as “Have you experienced any problems since the last contact?"

AEs must be recorded on the AE form in the eCRF with the initial eCRF report containing as much information as possible. One single AE form must be used per AE from start to resolution. For SAEs and ESIs, specific SAE and ESI data fields in the eCRF must also be filled in.

If the same type of AE occurs more than 1 day in a row with the same pattern (e.g. itching in the mouth for 5-10 minutes after intake of IMP) it is considered a recurrent AE. The AE form should be filled in with the start date and the description. Once the AE no longer recurs after IMP intake, the AE form should be completed with a stop date. If the AE then re-appears on a subsequent day, a new AE form should be filled in.

The investigator should record the diagnosis, if available. If no diagnosis is available the investigator should record each sign and symptom as individual AEs. Further, the diagnosis of the underlying disease should be reported instead of a procedure performed due to the disease (e.g. if a subject undergoes surgery due to appendicitis, appendicitis, and not the surgery performed, should be reported as the AE).

Solicited adverse events

The eDiary will be used to capture 15 pre-specified symptoms/signs, identified as potential local side effects of sublingual immunotherapy, as described in section 11.19 (**Passalacqua et al. 2013**). The reported symptoms/signs will be evaluated by investigator and reported as solicited AEs on the solicited AE form in the eCRF.

The duration in minutes of the solicited AEs occurring on Day 1 (visit 3) will be collected at the site. The start and stop time of solicited AEs that occur on Day 1 within 30 minutes of IMP intake will be monitored by site personnel, recorded in source documents, and entered into the

eCRF. After Day 1, standard AE reporting conventions will be utilised, as described above. For more details on solicited AEs, please see Section 11.19.

Eosinophilic oesophagitis

During the trial, subjects will be monitored for emerging symptoms of eosinophilic oesophagitis at the scheduled visits, as described in section 11.19.

Reporting of adverse events

The investigator must report all SAE and ESI information (initial as well as follow-up) to ALK within 24 hours after obtaining knowledge of the information. SAEs and ESIs will automatically be sent to ALK via the eCRF system. In case the eCRF system is unavailable during the 24 hour reporting timeline, SAEs and ESIs (including relevant eCRF pages e.g. demography, medical history, concomitant medication) must be reported by email to ALK. For the ESI of epinephrine/adrenaline administration, the symptoms and/or circumstances that triggered the use of the adrenaline/epinephrine auto-injector must be clearly recorded as AEs in the eCRF.

Non-serious AEs should be reported within 5 working days after obtaining knowledge of the information.

If requested, please forward supporting documents to ALK via email. Please state the trial ID (MT-12) and the subject and site ID on all documents.

IMPORTANT: Any information that could reveal the identity of the trial subject must be hidden or removed in the source documentation. Also, information that is not relevant for the subject and the subject's condition must be hidden or removed.

Email address: [redacted]

Emergency phone: [redacted]

The assessment of listedness is performed by ALK according to the reference safety information in the current version of the approved IB.

ALK will inform the regulatory authorities and IECs/IRBs in accordance with local requirements in force and the ICH guidelines for Good Clinical Practice (GCP) (**ICH 1996**).

Follow-up on adverse events

SAEs must be followed up until the outcome of the event is “recovered”, “recovered with sequelae” or “fatal” and until all queries have been resolved.

Ongoing SAEs can be closed with the term “not recovered” for chronic diseases (as evaluated upon medical evaluation by the investigator or the sponsor).

The investigator must respond to SAE follow-up requests from ALK without delay and no later than 14 days after receiving the request.

The investigator must ensure that the worst case severity and seriousness of an event is kept throughout the trial.

Non-serious AEs must be followed up until resolution or until the last follow-up phone contact.

Reporting of significant laboratory events

All laboratory reports must be reviewed by the investigator for significance.

Significant laboratory events present at screening should be recorded on the medical history page. Significant laboratory events found at the following visits, and which meet the definition of an AE, must be recorded on an AE page in the eCRF.

A significant laboratory event should be recorded as an AE if one of the following is applicable:

- It is abnormal and clinically significant (medical judgement by investigator)
- It leads to a change or discontinuation of treatment
- It fulfils a seriousness criterion
- It indicates a potential safety risk to the subject

The following constellation of laboratory values should by definition be considered an AE and reported accordingly:

- An elevated aspartate aminotransferase or alanine aminotransferase laboratory value that is greater than or equal to 3 times the upper limit of normal and an elevated total bilirubin lab value that is greater than or equal to 2 times the upper limit of normal and, at the same time an alkaline phosphatase laboratory value that is less than 2 times the upper limit of normal, as determined by way of protocol-specified laboratory testing or unscheduled laboratory testing.

The purpose of the criteria is to specify a threshold of abnormal hepatic tests that may require an additional evaluation for an underlying aetiology.

Reporting of medication errors, including overdose, abuse and misuse

Medication errors, including events of overdose, abuse or misuse must be reported on an AE form within 5 calendar days of obtaining the information. Reporting of overdoses must be based on actual IMP exposure and not on drug accountability procedures. For overdose cases, the descriptive terms accidental or intentional overdose should be used. If an event is classified as an SAE, it must be reported as such.

Reporting of pregnancies

The investigator must report information on pregnancy and pregnancy follow-up information including pregnancy complications, delivery and health of the infant until the age of one month within 14 calendar days of obtaining the information, using the pregnancy notification form and pregnancy follow-up form.

Complications in relation to pregnancy must be reported as AEs. In case of spontaneous abortion, any malformation of the foetus, foetal death, stillbirth or a congenital anomaly/birth defect/developmental delay, the event must be reported and followed up as an SAE.

Any abnormalities observed in a child (up to two years of age) and suspected to be related to intra-uterine exposure to the IMP should be reported to ALK.

Reporting of SAE and pregnancies after last follow-up phone contact

SAEs that in the opinion of the investigator are related to IMP that are brought to the attention of the investigator after the last follow-up phone contact must be reported immediately by using the contact details listed in this section.

All pregnancies occurring in trial subjects while exposed to IMP that are brought to the attention of the investigator after the last follow-up phone contact must be reported within 14 days by using the contact details listed in this section.

12.4 Data monitoring committee

To supplement the routine trial monitoring performed by the sponsor, an independent DMC will be established. The DMC is an independent, external committee composed of members whose expertise covers relevant specialities. The members of the DMC must not be involved with the trial in any other way (e.g., they cannot be trial investigators) and must have no competing interests that could affect their roles with respect to the trial.

Specific details regarding composition, responsibilities, and governance, including the roles and responsibilities of the various members and the sponsor; meeting facilitation; the trial governance structure; and requirements for and proper documentation of DMC reports, minutes, and recommendations will be described in a separate charter that is reviewed and approved by the DMC. The DMC will monitor trial data at an appropriate frequency, as described in the detailed DMC charter. The DMC will make recommendations to the sponsor regarding appropriate actions to ensure both subject safety and the continued ethical integrity of the trial.

13 Early termination of trial

ALK reserves the right to terminate the trial under the following conditions due to safety concerns and/or recommendation by the DMC.

If the trial is prematurely terminated or suspended, the investigator should promptly inform the subjects and ensure appropriate therapy and follow-up. Furthermore, the investigator and/or ALK should promptly inform the IEC/IRBs and provide a detailed written explanation. The regulatory authorities should be informed according to national regulations.

Early trial termination may be the result of any single criteria specified below:

1. IMP-related death of an individual
2. IMP-related anaphylactic shock in at least 2 subjects[34] defined as:
   Acute onset of an illness (minutes to several hours) with involvement of the skin, mucosal tissue, or both (e.g., generalised hives, pruritus, or flushing, swollen lips, tongue, or uvula) AND medically confirmed reduced BP with associated symptoms of end-organ dysfunction (e.g., hypotonia [collapse], syncope, incontinence).
   Low systolic BP for children is defined as less than 70 mmHg + (2 x age) from 1 to 10 years, and less than 90 mmHg for 11 to 17 years of age.
3. Recommendation by the DMC

IMP intake may only be resumed after the information has been presented to regulatory authorities, and regulatory authorities concur with continued IMP intake. In case of complete premature IMP discontinuation, participating investigators/subjects/parent/caregiver, the IEC/IRB, and the relevant regulatory authorities will be promptly informed.

[34] The criterion for 2 events is based on prior trial experience where it has been observed that events of severe anaphylactic reactions unrelated to IMP may occur in allergic subjects even with a temporal relationship to tablet administration.

14 Data handling

The diary in the trial will be an eDiary. The site will be trained in the use of eDiary by sponsor representatives. The sites will then train the subject and parent/caregiver in how to fill in the eDiary.

An eCRF will be used for this trial. The data is entered into the eCRF by a trained site investigator or other designated staff according to guidelines. A completed eCRF is required for each subject who signs an informed consent. All eCRFs must be completed in English.

The completed eCRFs and eDiaries are the property of the sponsor and must not be made available in any form to third parties (except for authorised representatives of appropriate governmental health or regulatory authorities) without written permission of the sponsor.

14.1 eCRF

Data generated by the trial site and relevant for the trial will be recorded in the eCRF.

The sponsor or its representative will supply sites with access to the eCRF. The sponsor or its representative will make arrangements to train the site staff in the use of the eCRF. There will be no access to the eCRF without documented training in the system.

All eCRF data must be verified and approved by an investigator at the site.

The CRA will review the eCRF for completeness and accuracy and instruct the personnel at the trial site to make any required corrections or additions according to an eCRF completion guideline.

The information entered into the database is systematically checked and errors or omissions will result in queries, which will appear in the eCRF for resolution.

Concomitant and previous medications entered into the database will be coded using the World Health Organisation Drug Reference List (WHO Drug, 2016 Q1 or higher).

Medical history and AEs will be coded using Medical Dictionary for Regulatory Activities (MedDRA 20.0 or higher).

The writing access to the eCRF will be revoked for all site staff prior to database lock, but the reading access will not be revoked until the investigator have received all subject PDF-files.

After database lock the sponsor will extract data from the eCRF system and convert the eCRF data to CDISC SDTM datasets.

14.2 eDiary

Starting at visit 2, an eDiary will be dispensed and data will be entered by the subject’s parent/caregiver together with the subject. The parent/caregiver will be trained in the eDiary by the investigator or designee. In addition, a training video with information on the eDiary will be available for the subjects and their parent/caregiver. The data will be reviewed by the investigator.

During the trial, data will be entered in an eDiary by the subject’s parent/caregiver and transferred to the vendor database on a daily basis. If the subject has missed more than 2 days in a row the investigator/designee will be notified by email and must contact the parent/caregiver. In addition, if the total compliance for a period is less than 80% the

investigator/designee must contact the parent/caregiver. The aim is for the overall compliance for a subject in the trial not to be below 80%.

There will be no other source documentation for these data than the vendor database.

After database lock, the sponsor will extract data from the eDiary system and convert the eDiary data to CDISC SDTM datasets. The investigator will also be provided with an electronic copy of the diary data collected at the specific site at the latest 3 months after end of trial, by the vendor. The eDiary data will be subject to periodic review by the sponsor. Findings during the review of the eDiary data will be evaluated by the investigator and updates/correction to the data can only be executed by the investigator. Documentation of the data load from the eDiary vendor to ALK will be described in the data handling report.

14.3 Query handling

Query handling at the trial site will be performed according to the guidelines for the eCRF and eDiary systems. Queries are created by programmed validation checks according to an edit check specification. Queries will also be created based on manual data checks.

All data changes/query decisions are created with an audit trail capturing the old information, the new information, identification of the user making the correction, the date the correction was made, and the reason for change.

14.4 Laboratory data

Laboratory samples will be processed at a central laboratory selected by ALK. When the samples have been analysed and the data released, the lab data will be imported automatically in the eCRF for investigator evaluation. The investigator must review and sign the laboratory reports. At the end of trial the laboratory data will be provided electronically to data management at ALK.

Immunological samples from Canada and Poland will be processed at ALK Research Laboratory and data will be provided electronically after database lock to data management.

Documentation of receipt and quality control of laboratory and immunological data will be provided in a data management report.

14.5 Database lock

When the database has been declared to be complete and accurate, the database will be locked and data will be unblinded, All accesses to the eCRF will be set as “read only”. CRAs and non-sponsor staff/persons “read only” accesses will be revoked from the eCRF when subjects PDF-files are received at site.

If changes to trial data become necessary after database lock this must be performed according to the current ALK SOP.

A data archive for the site subject data files are produced and sent to the site.
The investigator must sign and date the data archive approval form and send it back to the sponsor.

15 Statistical methods

Statistical analyses will be carried out by ALK Biometrics Department.

If nothing else is mentioned, all the statistical tests described in this section use a significance level of 5% and all tests and confidence intervals are two-sided. The null hypothesis is the hypothesis of no difference and the alternative to the null hypothesis is the hypothesis of a difference.

Descriptive statistics for numerical variables include summary tables displaying mean, standard deviation, median, 5%-quantile, 25%-quantile, 75%-quantile, 95%-quantile, minimum and maximum.

Descriptive statistics for categorical variables include frequencies tables that display numbers and percentage.

Before unblinding, a final SAP detailing the specifications given below will be prepared and agreed upon.

Any changes in the statistical methods compared to the final SAP will be documented in the ICTR. Post-hoc analyses, if any, will be clearly marked.

15.1 Sample size and power considerations

The primary efficacy endpoint is the average daily TCRS during the 8-week primary efficacy assessment period.

The primary efficacy analysis is based on treatment comparison performed using a two-sided t-test on a 5% level of significance in a linear mixed effect (LME) model assuming unequal variances. The TCRS is square root transformed in the LME model in order to obtain a better approximation to a normal distribution. Least squares means are then back-transformed to the original scale by taking the square. In addition, the relative difference of the back-transformed least squares means will be calculated together with 95% confidence limits. The latter will be calculated based on Fieller's theorem.

Superiority of active treatment compared to placebo is confirmed when the p-value for the primary efficacy analysis is <0.05 in the t-test. An additional FDA acceptance criteria for the trial is a point estimate of the relative difference to placebo of no more than -15% with an associated upper bound of the 95% CI being at most -10%.

Data from previous adult phase III trials of the HDM tablet conducted in Europe (MT-06) and North America (P001) and data from a previous paediatric trial (5-17 years of age) of the HDM tablet conducted in Japan (TO-203-3-3) will be used for power calculation. Table 13 shows the mean and standard deviation of the square root transformed TCRS by treatment group for the MT-06 trial. The power calculation will be based on the mean for placebo and the standard deviation for the placebo and the 12 SQ-HDM treatment group observed in the MT-06 trial due to the similar nature of the trial design for the MT-12 and MT-06 trial. Table 14 shows the estimated effect sizes in MT-06, P001 and TO-203-3-3 for different age groups. Power calculation will be performed based on the assumption that the effect size is similar to the effect size seen in the paediatric trial TO-203-3-3 for the age group 5-11 years (~21%). In addition power calculation will be based on the more conservative assumption that the effect size is similar to the effect sizes observed in the previous adult trials MT-06 and P001 conducted in Europe and North America (~17-18%).

Table 15 shows the results for the power calculation. Power calculation for fulfilling the acceptance criteria of a p-value <0.05 is based on Satterthwaite unpooled two-sided t-test on a 5% level of significance. Power calculation for fulfilling the acceptance criteria of an upper bound of the confidence limit being at most -10% (95% UCL ≤ -10%) is conducted by simulations using Fieller's theorem.

Table 13 Mean and standard deviation of TCRS by treatment group in MT-06

	**MT-06**	
**Square root transformed TCRS**	*Placebo*	*12 SQ-HDM*
Raw Mean	2.65	2.40
Standard deviation	0.89	0.95

Table 14 Effects size of TCRS in MT-06, P001 and PO-203-3-3

**Trial**	**MT-06**	**P001**		**TO-203-3-3**		
Region	*Europe*	*North America*		*Japan*		
Age group	18-65	18-65	12-17[A]	5-17	5-11[A]	12-17[A]
N	582	1186	160	427	247	180
Effect size	-18.1%[B]	-17.2%[C]	-22.4%[C]	-22.5%[B]	-20.8%[B]	-25.9%[B]

[A] Subgroup analysis, [B] Relative difference (Active-Placebo)·100%/Placebo in adjusted means [C] Relative difference (Active-Placebo)·100%/Placebo in medians

Table 15 Power calculations based on different assumptions of the effect size

**Effect size**	**-18%**		**-21%**	
Acceptance criterion	*p-value < 0.05*	*95% UCL ≤ -10%*	*p-value <0.05*	*95% UCL ≤ -10%*
**Sample size per arm**	**Power**			
300 subjects	91%	35%	97%	58%
500 subjects	99%	51%	100%	80%
580 subjects	100%	58%	100%	85%

95% UCL, upper bound of 95% confidence interval.

Based on Table 15, a sample size of 580 per treatment arm is chosen. With an effective sample size of 580 subjects per treatment arm, the MT-12 trial will have:

- 85% power (2-sided, α=0.05) to obtain an upper bound of the 95% CI for a relative difference of no more than -10% if assuming an effect size of -21%
- >90% power (2-sided, α=0.05) to detect a statistically significant difference (p-value<0.05) if assuming an effect size of -18%

When further adjusting for a drop-out that may be approximately 15% based on experience from MT-06 and P001, the proposed sample size per treatment arm for the MT-12 trial is 682 subjects (N_{total}=1364 ~ 1370 subjects).

15.2 Analysis data sets

The total analysis set comprises all subjects who entered the trial. This analysis set includes screening failures. The total population will be used for listing reasons for screening failures and AEs before randomisation.

The full analysis set (FAS) comprises all randomised subjects who received at least 1 dose of IMP. Subjects will be analysed according to the treatment group to which they are randomised. The FAS will be used for all baseline/demography tables and subject listings. For efficacy analysis, the missing data approach will be to use FAS with observed data of the relevant endpoint (FAS, Obs). FAS with imputation of missing data will be used for sensitivity analyses of the primary endpoint.

The safety analysis set (SAF) will be used for safety analyses. The SAF will include all randomised subjects who received at least 1 dose of IMP. Subjects will be included in the treatment group corresponding to the IMP they actually received during the trial. SAF will be used for safety tables and listings.

15.3 Subject disposition

A table of subject disposition by treatment group displaying number and percentage of subjects screened, randomised, included in the FAS, SAF, discontinued and the primary reason for discontinuation will be presented. In addition, the number and percentage of subjects in FAS with observation of the primary endpoint (FAS, Obs) will be displayed.

The over all drop-out distribution will be displayed by a Kaplan-meier plot. Cumulative incidence plot will be used to show cause specific drop-out distribution.

15.4 Demographic and baseline characteristics

Demographic (including age, year of birth, sex, race and ethnic origin) and baseline characteristics (including baseline TCRS, rhinitis DSS, rhinitis DMS, % of predicted FEV_1, duration of HDM AR, sensitisation type, medical history) will be summarised by treatment group.

15.5 Extent of exposure

IMP accountability (number of daily doses used) is the difference between the number of daily doses dispensed and the number of daily doses returned. IMP compliance is the number of daily doses used divided by the duration of the treatment period in days and multiplied with 100. A treatment year is the number of daily doses divided by 365. Duration of IMP treatment period in days, IMP accountability, IMP compliance and treatment years will be displayed in summary tables by treatment groups.

15.6 Previous and concomitant therapy

Previous and concomitant medication and illness will be summarised by means of descriptive statistics.

15.7 Efficacy analyses

The primary endpoint is the average TCRS during the primary efficacy assessment period (period 4). The primary efficacy analysis will be based on the FAS population and the missing data approach is to use observed data only. Thus, only subjects in FAS with observation of the primary endpoint (FAS, Obs) will contribute to the primary efficacy analysis.

The primary endpoint will be analysed using a linear mixed effect model with the square root of the TCRS as the response variable, treatment and cohort as a fixed factors, the square root of the baseline average TCRS as a covariate and country/region within cohort as a random effect. Different residual error for each treatment group will be specified in the linear mixed effect model.

The between treatment comparison will be performed using a t-test in the linear mixed effect model. The degree of freedom in the t-test is calculated based on the Kenward and Roger approximation. The null hypothesis in the t-test is that the mean is equal for the active group and placebo. The alternative hypothesis is that the mean is different for the active group and placebo. The result of the primary efficacy analysis is considered successful when the p-value from the t-test is < 0.05. The difference in (the back-transformed) adjusted means will be calculated together with 95% confidence intervals. In addition, the relative difference of the (back-transformed) adjusted means will be reported together with 95% confidence limits. The latter is calculated based on Fieller's theorem.

If the square root transformation does not result in a good approximation to the normal distribution, no transformation will be applied if this improves the approximation to a normal distribution. When it is not possible (with or without transformation) to obtain a good approximation to the normal distribution the non-parametric Wilcoxon Rank Sum test will be applied and reported together with the Hodges-Lehmann estimator.

The assumption of normally distributed residuals underlying the parametric linear mixed effect model will be evaluated by visual inspections of quantile-quantile plots.

Key secondary efficacy analysis

The key secondary endpoints will be analysed in the same way as described for the primary efficacy endpoint based on all subjects in FAS with observations during the efficacy assessment period (FAS, Obs) and using a LME model. The default transformation for the response in the LME models for the key secondary endpoints is the square root transformation.

Other efficacy analyses

All endpoints regarding average scores during the efficacy assessment period will be analysed similar to the primary endpoint, i.e. with a linear mixed effect model including treatment group, and cohort as fixed factors, the corresponding average baseline score as a covariate and country/region within cohort as a random effect. Different residual error for each treatment group will be specified in the linear mixed effect model. The default transformation for the secondary endpoint "average rhinoconjunctivitis DSS", "average rhinoconjunctivitis DMS", average asthma DSS and "average combined symptom and medication score (CSMS)" is the square root transformation. By default no transformation will be applied to the overall PRQLQ score.

Binary endpoints (e.g. mild days and rhinitis exacerbations) will be analysed using a generalised linear mixed effect model.

In case of a positive outcome of the primary efficacy analysis, clinical effect will be evaluated using the average TCRS collected after 8 and 16 weeks of treatment. Data will be analysed using a mixed-effect model for repeated measurement (MMRM) with a square root transformed response. Visit, treatment group and their 2-way interaction and cohort nested within visit will be included as fixed factors, the square root of the average baseline score nested within visit as a covariate and country/region nested within cohort and visit as a random effect. The resulting back-transferred adjusted means and the difference in back-transferred adjusted means with 95% confidence limits will be plotted against weeks (visit) with IMP treatment.

In addition, all efficacy endpoints will be presented in summary tables.

Details for the evaluation of immunological parameters will be described in the SAP.

15.8 Safety analyses

15.8.1 Analyses of AEs

All TEAEs will be summarised by treatment group, MedDRA system organ class and preferred term displaying number of subjects in treatment group, number and percentage of subjects having the event as well as number of events. Furthermore, TEAEs will be summarised according to severity, relationship, outcome and seriousness.

Solicited AEs will be summarised separately.

The analyses will be described further in SAP.

15.8.2 Analyses of other safety parameters

Physical examination assessments, laboratory assessments, vital signs and FEV_1 will be summarised by means of descriptive statistics.

15.9 Multiplicity

The issue of multiple testing for the primary and 3 key secondary efficacy endpoints will be handled by using hierarchical testing. The order of the hierarchical tests is:

1. Superiority testing of the HDM SLIT-tablet over placebo with respect to the average TCRS during the primary efficacy assessment period
2. Superiority testing of the HDM SLIT-tablet over placebo with respect to the average rhinitis DSS during the primary efficacy assessment period
3. Superiority testing of the HDM SLIT-tablet over placebo with respect to the average rhinitis DMS during the primary efficacy assessment period
4. Superiority testing of the HDM SLIT-tablet over placebo with respect to average TCS during the primary efficacy assessment period

A lower test can only be evaluated when the null hypothesis in the former test has been rejected.

15.10 Interim analyses

No interim analyses are planned.

15.11 Handling of missing data

For the primary efficacy analysis, no imputation of data will be carried out in case of missing data. This means that for the primary efficacy analysis subjects who withdrew prior to the primary efficacy assessment period visit (period 4) will not contribute to the efficacy analysis. To evaluate the sensitivity of the results of the primary efficacy analysis with respect to missing data, the following sensitivity analyses will be performed.

Sensitivity analysis 1: A mixed-effect model for repeated measurement (MMRM) analysis based on FAS with at least one post randomisation observation of TCRS. The square root of the average TCRS is the response variable, baseline square root average TCRS nested within visit is a covariate, visit (TC1, TC2, V6-V7) and cohort nested within visit are fixed factors and country/region nested within cohort is a random variable. Different residual errors for each treatment group will be specified and the within-subject correlated errors are modelled by an unstructured (UN) covariance. From the MMRM model the back-transformed (squared) adjusted means and difference in back-transformed adjusted means together with 95% confidence limits will be calculated for period 4 (V6-V7).

Sensitivity analysis 2: A LME analysis based on FAS with multiple imputation (MI) of missing data. Subjects with no observation of TCRS in period 4 due to the discontinuation reasons “IMP-related AEs” or “Lack of efficacy” will be imputed based on the placebo arm using an unconditional parametric MI method. Subjects with no observation of TCRS in period 4 due to discontinuation reasons other than “IMP-related AEs” or “Lack of efficacy” will be imputed based on own arm using an unconditional parametric MI method. The imputation conservatively assumes a treatment effect similar to the placebo group for subjects in the active arm who discontinue due to “IMP-related AEs”/”Lack of efficacy” had the subjects continued on treatment into period 4. Multiple imputation will be performed on the square root transformed average TCRS during period 4 using a monotone regression method with baseline square root average TCRS as a covariate and country/region as a factor. In total 500 imputed datasets will be generated. Each copy will be analysed using the LME model specified for the primary efficacy analysis in section 15.7. The resulting estimates and standard deviations for the multiple data sets are finally pooled to one single estimate and associated standard deviation using the method of Rubin.

16 Quality assurance and control

16.1 Monitoring

Regular monitoring visits will be performed according to ICH GCP. Data will be evaluated for compliance with the protocol and accuracy in relation to source documents. In accordance with written SOP, the CRAs will verify that the clinical trial is conducted and data are generated, documented and reported in compliance with the protocol, GCP and the applicable regulatory requirements.

16.2 Source data and access to source documents

Prior to start of recording of data from subjects, the investigator, with the aid of the CRA, will prepare a source data location agreement to document where the first recording of data is done.

As a minimum requirement, the following eCRF data must be source data-verifiable in source documentation other than the eCRF:

- Date of informed consent
- Subject's month[35] and year of birth
- Date of screening visit
- Confirmation of participation in the trial (trial ID, subject number, diagnosis)
- Confirmation of subject eligibility (in/exclusion criteria)
- Prior and concomitant diseases and medication
- Relevant medical history (incl. specific allergy and asthma history and date of diagnosis)
- Any AEs and SAEs should be described in detail
- Date and number of each trial visit including signature and/or initials of persons conducting the trial visit
- Date and information of any relevant telephone contact with the subject and signature and/or initials of persons conducting or receiving the call
- IMP dispensed/returned
- Subject discontinuation from the trial including reason

Documentation of assessments made during the trial e.g. FEV_1, SPT and laboratory results must be kept in the subject's medical record – evaluated, signed and dated by an investigator at the trial site. Documentation on thermo-sensitive paper must be copied and signed by the investigator. The copy signed by the investigator should be kept together with the original in the subject's medical record.

The following data may be recorded directly in the eCRF and is then considered to be source data (if acceptable by national legislation and hospital routine):

- Demography and body measurements
- Vital signs
- Physical examination

The investigator must give the CRA direct access to examine, analyse and verify any medical records or reports to procedures, source documentation, data records and reports used, referenced or created as part of the conduct of this trial (e.g. relevant hospital or medical records), to confirm consistency with eCRF entries.

The CRA will examine the electronic medical record system and decide one of the following options for source data verification:

- <u>Option 1</u>
  If an audit trail is available, the CRA may choose either to perform source data verification through a direct comparison of the eCRF and the electronic medical record

[35] For countries where it is allowed

- Option 2
  If no audit trail is available, relevant source data from electronic medical records should be printed out by the investigator or delegate preferably at the day of the monitoring visit. The investigator or delegate must sign and date the print-out to confirm that the print and the electronic source data are identical. The CRA must verify the original source data at least once during the trial.

Either option will be agreed with the investigator prior to trial start.

All documents must be stored safely under confidential conditions. On all trial specific documents, other than the signed consent, the subject will be referred to by the subject ID number. If ALK becomes aware of the identity of a subject, ALK is bound to keep this information confidential and to take immediate actions to delete any information received that identifies the subject.

16.3 Investigator site file – and other trial documentation

The investigator must maintain source documents for each subject in the trial in accordance with local legislation.

The investigator must retain the investigator site file for at least 25 years or in accordance with local legislation.

No trial related documentation may be destroyed by the investigator without prior written agreement with ALK. The investigator agrees to adhere to the document retention procedures by signing the protocol.

Should the investigator choose to transfer the trial documents to another physician or institution, ALK must be notified.

16.4 Protocol compliance

The instructions in the protocol must be followed. If deviations occur, the investigator must inform the CRA, and the implications of the deviation must be reviewed and discussed. Deviations must be documented (or included in eCRF data). In addition, deviations must be accompanied by a description of the deviation, the reason for the deviation, the relevant dates (start and resolved) and the action taken. Deviation reports and supporting documentation must be kept in the investigator's file and in the ALK trial master file.

16.5 Audit

ALK may conduct audit(s) of clinical research activities in accordance with internal SOPs to evaluate compliance with GCP and international and local guidelines and regulations.

The investigator must be available during the audit and give the auditors direct and unlimited access to source documentation and data, records and reports used, referenced or created as part of the performance of this trial.

17 Ethics and regulatory procedures

17.1 Statement of compliance

This trial must be carried out in compliance with the protocol, which is designed to ensure adherence to the Declaration of Helsinki and the principles of GCP, as described in:

- The World Medical Association Declaration of Helsinki - Ethical Principles for Medical Research Involving Human Subjects (**World Medical Association 2013**)
- International Conference on Harmonisation Harmonised Tripartite Guideline for Good Clinical Practice E6(R2) (**ICH 2016**)
- EU Directive 2001/20/EC of the European Parliament and of the Council on the Approximation of the Laws, Regulations and Administrative Provisions of the Member States Relating to the Implementation of GCP in the Conduct of Clinical Trials on Medicinal Products for Human Use. 2001 (**2001**)
- European Commission. Commission Directive 2005/28/EC of 8 April 2005 laying down principles and detailed guidelines for GCP as regards IMPs for human use, as well as the requirements for authorisation of manufacturing or importation of such products. 2005 (**European Commission 2005**)
- FDA regulations relating to good clinical practice and clinical trials, Protection of Human Subjects (21 CFR Part 50)

17.2 Disclosure and confidentiality

By signing the protocol, the investigator agrees to keep all information, data and materials whether in oral, written, graphic, electronic or other form provided by ALK or a third party acting on behalf of or at the instruction of ALK in strict confidentiality.

Trial documents provided by ALK (protocol, IB, eCRFs and other material) should be stored appropriately to ensure their confidentiality. The information provided by ALK to the investigator may not be disclosed to others except as expressly authorised by this protocol or the clinical trial agreement or with the prior written consent of ALK.

The investigator may disclose confidential information to employees of the investigator, hospital authorities and IECs/IRBs on a need-to-know basis and only if the aforementioned parties are bound or obligated by provisions of confidentiality no less strict than imposed upon the investigator under this protocol or the clinical trial agreement. Further, the investigator may disclose confidential information set out in the protocol to the extent necessary to obtain informed consent from subjects who wish to participate in the trial.

Any data, results, reports, findings, discoveries and any other information developed or collected during this trial shall be regarded as ALK's confidential information until published.

Financial disclosure from the investigators will be obtained before the trial.

17.3 Subject confidentiality

The trial staff should ensure that the subjects' anonymity is maintained. The subjects will be identified only by a subject ID number on the eCRF and any electronic database owned by ALK.

All documents will be stored securely and only accessible by trial staff and authorised personnel.

17.4 Data protection

All data will be handled and stored according to General Data Protection Regulation (GDPR) (The European parliament 2016).

Investigator

By signing this protocol, the investigator recognises that certain personal identifying information with respect to the investigator and all sub-investigators and trial site personnel, may be used and disclosed for trial management purposes, as part of a regulatory submissions, and as required by law. This information may include:

- Name, address, telephone number and email address
- Hospital or clinic address and telephone number
- Curriculum vitae or other summary of qualifications and credentials
- Financial disclosure information
- Other professional documentation

Consistent with the purposes described above, this information may be transmitted to the ALK, affiliates and ALK representative, in the investigators country and other countries, including countries that do not have laws protecting such information.

Additionally, the investigator's name and business contact information may be included when reporting certain SAEs to regulatory authorities or to other investigators. By signing this protocol, the investigator expressly consents to these uses and disclosures.

In order to facilitate contact between investigators, ALK may share an investigator's name and contact information with other participating investigators upon request.

Subjects

Subjects will be assigned a unique identifier, a subject number. Any subject records or datasets that are transferred to ALK will contain the identifier only; subject names or any information which would make the subject identifiable will not be transferred. This way the subject data will go through a pseudonymisation process, which means that the data cannot be attributed to a specific subject without the use of additional information.

The subject and any biological material obtained from the subject will be identified by subject number, visit number and trial ID. Appropriate measures such as encryption or leaving out certain identifiers will be enforced to protect the identity of subjects as required by local, regional and national requirements.

The subject must be informed that his/her personal trial related data will be used by ALK in accordance with local data protection law. The disclosure of the data must also be explained to the subject.

The subject must be informed that his/her medical records may be examined by auditors or other authorised personnel appointed by ALK, by appropriate IRB/IEC members, and by inspectors from regulatory authorities.

Transfer of data

If data are transferred to or accessed by third countries, that is, countries outside EU/EEA countries, the data will be protected to have an adequate level of data protection principles.

Description of arrangements

To ensure the safekeeping of clinical trial data ALK has Standard Operating Procedures that defines how data shall be managed including handling of security data breaches. When data is managed by external parties, written agreements are in place to ensure that the data is handled according to ALK's instructions/standards.

17.5 IEC/IRB/regulatory authorities

Before initiation of this trial, the protocol, the proposed subject informed consent form and other information to subjects as well as other documents required, must be reviewed by a properly constituted IEC/IRB and provided to the national (and local, if applicable) regulatory authority.

A signed and dated statement that the protocol and the subject informed consent form have been approved by the IEC/IRB and the regulatory authority must be obtained by ALK before trial initiation.

17.6 Inspections

An IEC/IRB or a national or international regulatory authority may also wish to conduct an inspection (during the trial or after its completion). If an inspection is requested by a regulatory authority, the investigator must inform ALK of the request immediately. The investigator or ALK should agree (in accordance with the prevailing law) with the inspectors that ALK shall have the right to be present at any inspection or investigation. The investigator or ALK should agree with the inspector that ALK may conduct and control applicable action arising from the inspections at ALK's expense.

The investigator must be available during the inspection and give the inspectors direct and unlimited access to source documentation and data, records and reports used, referenced or created as part of performance of this trial.

17.7 Protocol amendment and other changes in trial conduct

Substantial changes to this protocol require a protocol amendment that must be signed off by ALK and the investigator(s) and be approved by IEC/IRB and/or regulatory authorities as applicable before implementation.

The requirements for approval of the substantial changes should in no way prevent any immediate action from being taken by the investigator or by ALK in the interest of preserving the safety of all subjects included in the trial.

Amendments not considered substantial such as administrative changes will only be submitted to the regulatory authorities once another substantial amendment must be submitted or together with the end of trial notification unless national legislation requires otherwise.

18 Reporting and publication

18.1 Integrated clinical trial report

Data will be reported in an ICTR in compliance with the requirements of the current version of ICH E3: Structure and Content of Clinical Trial Report (**ICH 1995**), ICH GCP Guidelines (**ICH 2016**), and ALK SOPs.

The signatory investigator will review and sign the ICTR.

18.2 Publication of results

ALK retains exclusive ownership of all data, results, reports, findings, discoveries and any other information developed or collected during this trial and ALK shall have the exclusive right to use all such information for any purpose, including, but not limited to, use of the results and data either in the form of eCRF (or copies of these), or in the form of a report, with or without comments and with or without analysis, in order to submit them to the regulatory authorities of any country.

By signing the investigator agreement, the investigator agrees that the results of this trial may be used for submission to national and/or international registration and supervising authorities. The authorities will be notified of the investigator's name, address, qualifications and extent of involvement.

It is envisaged that the findings of this trial, including sub-analysis, and if relevant the epidemiology of the screened population and the selection process, will, in due time and by mutual agreement, be published in international journals, theses and/or presented at scientific meetings or symposia. All presentations and publications must be reviewed by ALK prior to public presentation or submission. For multi-site trials, it is mandatory that the primary publication is based on data from all trial sites, analysed as stipulated in the protocol and in the SAP. Authorship is based on the International Committee of the Medical Journal Editors' Uniform Requirements (Vancouver Declaration). If the number of authors is restricted, selection will be based on fulfilment of 1) involvement in the development of the protocol and 2) being coordinating investigator.

Investigators agree not to present data gathered from one trial site or a group of trial sites before the primary publication has been accepted for publication unless formally agreed by all other investigators and ALK.

ALK shall be provided with copies of any proposed publication or presentation at least 60 days in advance of the submission of such proposed publication or presentation to a journal, editor, or other third party. ALK has the right to review and comment on any such publication or presentation within 60 days of receipt, but cannot prevent publication of findings. The investigator agrees that all reasonable comments made by ALK will be incorporated into the publication. Furthermore, the investigator agrees that the investigator shall, at ALK's request exclude or delete any confidential information, except trial results generated, from the proposed publication or presentation.

ALK will review the presentations and publications for accuracy (thus avoiding potential discrepancies with submissions to regulatory authorities), verify that the confidential information is not being inadvertently divulged and provide any relevant supplementary information. Upon ALK's request, the investigator shall delay a publication or presentation for 6 months from ALK's

receipt of the publication or presentation to permit ALK to file a patent application or take other steps as necessary to protect the confidential information (including trial results of ALK's).

19 Finance and insurance

ALK subscribes to an insurance policy covering, in its terms and provisions, ALK's legal liability for injuries caused to participating subjects and arising out of these trial procedures performed strictly in accordance with this protocol as well as with applicable law and professional standards.

The compensation to the investigators for work performed under this protocol will be set out in separate clinical trial agreements with the investigators.

20 Trial organisation

The telephone numbers and fax numbers of relevant ALK staff are listed in the investigator's site file.

The title, name, address and contact details of investigators and clinical research organisations e.g. subcontractors for project management, clinical trial packaging, monitoring, central laboratory etc. are listed in Appendix 4: Trial organisation.

21 Reference list

Directive 2001/20/EC of the European Parliament and of the Council on the Approximation of the Laws, Regulations and Administrative Provisions of the Member States Relating to the Implementation of Good Clinical Practice in the Conduct of Clinical Trials on Medicinal Products for Human Use. 2001.

Ref Type: Report

Alvarez-Cuesta,E., Bousquet,J., Canonica,G.W., Durham,S.R., Malling,H.J. & Valovirta,E. 2006. Standards for practical allergen-specific immunotherapy. Allergy, 61 Suppl 82, 1-20.

Arbes,S.J., Jr., Gergen,P.J., Elliott,L. & Zeldin,D.C. 2005. Prevalences of positive skin test responses to 10 common allergens in the US population: results from the third National Health and Nutrition Examination Survey. J Allergy Clin Immunol, 116, 377-383.

Arlian,L.G. & Platts-Mills,T.A. 2001. The biology of dust mites and the remediation of mite allergens in allergic disease. J. Allergy Clin Immunol., 107, S406-S413.

Arshad,S.H., Tariq,S.M., Matthews,S. & Hakim,E. 2001. Sensitization to common allergens and its association with allergic disorders at age 4 years: a whole population birth cohort study. Pediatrics, 108, E33.

Bacharier,L.B., Boner,A., Carlsen,K.H., Eigenmann,P.A., Frischer,T., Gotz,M., Helms,P.J., Hunt,J., Liu,A., Papadopoulos,N., Platts-Mills,T., Pohunek,P., Simons,F.E., Valovirta,E., Wahn,U. & Wildhaber,J. 2008. Diagnosis and treatment of asthma in childhood: a PRACTALL consensus report. Allergy, 63, 5-34.

Bauchau,V. & Durham,S.R. 2004. Prevalence and rate of diagnosis of allergic rhinitis in Europe. Eur. Respir. J., 24, 758-764.

Bousquet,J., Khaltaev,N., Cruz,A.A., Denburg,J., Fokkens,W.J., Togias,A., Zuberbier,T., Baena-Cagnani,C.E., Canonica,G.W., van,W.C., Agache,I., it-Khaled,N., Bachert,C., Blaiss,M.S., Bonini,S., Boulet,L.P., Bousquet,P.J., Camargos,P., Carlsen,K.H., Chen,Y., Custovic,A., Dahl,R., Demoly,P., Douagui,H., Durham,S.R., van Wijk,R.G., Kalayci,O., Kaliner,M.A., Kim,Y.Y., Kowalski,M.L., Kuna,P., Le,L.T., Lemiere,C., Li,J., Lockey,R.F., Mavale-Manuel,S., Meltzer,E.O., Mohammad,Y., Mullol,J., Naclerio,R., O'Hehir,R.E., Ohta,K., Ouedraogo,S., Palkonen,S., Papadopoulos,N., Passalacqua,G., Pawankar,R., Popov,T.A., Rabe,K.F., Rosado-Pinto,J., Scadding,G.K., Simons,F.E., Toskala,E., Valovirta,E., Van,C.P., Wang,D.Y., Wickman,M., Yawn,B.P., Yorgancioglu,A., Yusuf,O.M., Zar,H., nnesi-Maesano,I., Bateman,E.D., Ben,K.A., Boakye,D.A., Bouchard,J., Burney,P., Busse,W.W., Chan-Yeung,M., Chavannes,N.H., Chuchalin,A., Dolen,W.K., Emuzyte,R., Grouse,L., Humbert,M., Jackson,C., Johnston,S.L., Keith,P.K., Kemp,J.P., Klossek,J.M., Larenas-Linnemann,D., Lipworth,B., Malo,J.L., Marshall,G.D., Naspitz,C., Nekam,K., Niggemann,B., Nizankowska-Mogilnicka,E., Okamoto,Y., Orru,M.P., Potter,P., Price,D., Stoloff,S.W., Vandenplas,O., Viegi,G. & Williams,D. 2008. Allergic Rhinitis and its Impact on Asthma (ARIA) 2008 update (in collaboration with the World Health Organization, GA(2)LEN and AllerGen). Allergy, 63 Suppl 86, 8-160.

Bousquet,J., Lockey,R., Malling,H.J., varez-Cuesta,E., Canonica,G.W., Chapman,M.D., Creticos,P.J., Dayer,J.M., Durham,S.R., Demoly,P., Goldstein,R.J., Ishikawa,T., Ito,K., Kraft,D., Lambert,P.H., Lowenstein,H., Muller,U., Norman,P.S., Reisman,R.E., Valenta,R., Valovirta,E. &

Yssel,H. 1998. Allergen immunotherapy: therapeutic vaccines for allergic diseases. World Health Organization. American academy of Allergy, Asthma and Immunology. Annals of allergy, asthma & immunology : official publication of the American College of Allergy, Asthma, & Immunology, 81, 401-405.

Bousquet,J., Van Cauwenberge,P., Khaltaev,N., Aria Workshop Group & World Health Organization 2001. Allergic rhinitis and its impact on asthma. J. Allergy Clin. Immunol., 108, S147-S334.

Brozek,J.L., Bousquet,J., Baena-Cagnani,C.E., Bonini,S., Canonica,G.W., Casale,T.B., van Wijk,R.G., Ohta,K., Zuberbier,T. & Schunemann,H.J. 2010. Allergic Rhinitis and its Impact on Asthma (ARIA) guidelines: 2010 revision. J. Allergy Clin. Immunol, 126, 466-476.

Calderon,M.A., Kleine-Tebbe,J., Linneberg,A., de,B.F., Hernandez Fernandez de,R.D., Virchow,J.C. & Demoly,P. 2015. House Dust Mite Respiratory Allergy: An Overview of Current Therapeutic Strategies. J Allergy Clin Immunol Pract., 3, 843-855.

Canonica,G.W. 2007. A survey of the burden of allergic rhinitis in Europe. Allergy, 62 supp, 17-25.

Canonica,G.W., Baena-Cagnani,C.E., Bousquet,J., Bousquet,P.J., Lockey,R.F., Malling,H.-J., Passalacqua,G., Potter,P. & Valovirta,E. 2007. Recommendations for standardization of clinical trials with Allergen Specific Immunotherapy for Respiratory allergy. A statement of the World Allergy Organization (WAO) taskforce. Allergy, 62, 317-324.

Corzo,J.L., Carrillo,T., Pedemonte,C., Plaza-Martin,A.M., Martín Hurtado,S., Dige,E. & Calderon,M.A. 2014. Tolerability during double-blinded randomised phase I trials with the house dust mite allergy immunotherapy tablet in adults and children. J Investig Allergol Clin Immunol, 24, 154-161.

Cox,L., Nelson,H., Lockey,R., Calabria,C., Chacko,T., Finegold,I., Nelson,M., Weber,R., Bernstein,D.I., Blessing-Moore,J., Khan,D.A., Lang,D.M., Nicklas,R.A., Oppenheimer,J., Portnoy,J.M., Randolph,C., Schuller,D.E., Spector,S.L., Tilles,S. & Wallace,D. 2011. Allergen immunotherapy: a practice parameter third update. J. Allergy. Clin. Immunol., 127, S1-55.

Demoly,P., Emminger,W., Rehm,D., Backer,V., Tommerup,L. & Kleine-Tebbe,J. 2016. Effective treatment of house dust mite-induced allergic rhinitis with 2 doses of the SQ HDM SLIT-tablet: Results from a randomized double-blind, placebo-controlled phase III trial. J Allergy Clin Immunol, 137, 444-451.

EMA & PDCO. EMA/PDCO Standard Paediatric Investigation Plan for Allergen Products for Specific Immunotherapy. EMA/PDCO/737605/2009, Revision 4, 1-13. 2015.

Ref Type: Report

EMEA. Guideline on the Clinical Development of Medicinal products for the Treatment of Allergic Rhinoconjunctivitis. CHMP/EWP/2455/02, 1-9. 2004.

Ref Type: Report

EMEA. Reflection Paper: Formulations of choice for the paediatric population. 2006.

Ref Type: Report

EMEA. Guideline on the clinical development of products for specific immunotherapy for the treatment of allergic diseases. CHMP/EWP/18504/2006[Final]. 2008.

Ref Type: Report

Epstein,T.G., Calabria,C., Cox,L.S. & Dreborg,S. 2017. Current Evidence on Safety and Practical Considerations for Administration of Sublingual Allergen Immunotherapy (SLIT) in the United States. J Allergy Clin Immunol Pract., 5, 34-40.

European Commission. Commission Directive 2005/28/EC of 8 April 2005 laying down principles and detailed guidelines for good clinical practice as regards investigational medicinal products for human use, as well as the requirements for authorisation of manufacturing or importation of such products. 2005.

Ref Type: Report

FDA. Guidance for Industry: Allergic Rhinitis: Developing Drug Products for Treatment. 2018. U.S. Department of Health and Human Services, FDA, CDER.

Ref Type: Report

GINA Executive Committee. Global Initiative for Asthma; Global Strategy for Asthma Management and Prevention. 2017. National Heart, Lung and Blood Institute, National Institute of Health, Bethesda, Maryland, USA.

Ref Type: Report

Gotzsche,P.C. & Johansen,H.K. 2008. House dust mite control measures for asthma: systematic review. Allergy, 63, 646-659.

Gruchalla,R.S., Pongracic,J., Plaut,M., Evans,R., III, Visness,C.M., Walter,M., Crain,E.F., Kattan,M., Morgan,W.J., Steinbach,S., Stout,J., Malindzak,G., Smartt,E. & Mitchell,H. 2005. Inner City Asthma Study: relationships among sensitivity, allergen exposure, and asthma morbidity. J Allergy Clin Immunol, 115, 478-485.

ICH. ICH Harmonised Tripartite Guideline Topic E3: Structure and Content of Clinical Study Reports. 1995.

Ref Type: Report

ICH. ICH Harmonised Tripartite Guideline Topic E6 (R1): Guideline for Good Clinical Practice. 1996.

Ref Type: Report

ICH. Integrated Addendum to ICH E6(R1): Guideline for Good Clinical Practice E6(R2). 2016.

Ref Type: Report

ICH Harmonised Tripartite Guideline. ICH Harmonised Tripartite Guideline - Statistical Principles for Clinical Trials E9. 1998.

Ref Type: Report

Jacobsen,L., Niggemann,B., Dreborg,S., Ferdousi,H.A., Halken,S., Host,A., Koivikko,A., Norberg,L.A., Valovirta,E., Wahn,U. & Moller,C. 2007. Specific immunotherapy has long-term preventive effect of seasonal and perennial asthma: 10-year follow-up on the PAT study. Allergy, 62, 943-948.

Juniper,E.F., Howland,W.C., Roberts,N.B., Thompson,A.K. & King,D.R. 1998. Measuring quality of life in children with rhinoconjunctivitis. J Allergy Clin Immunol, 101, 163-170.

Lodge,C.J., Lowe,A.J., Gurrin,L.C., Hill,D.J., Hosking,C.S., Khalafzai,R.U., Hopper,J.L., Matheson,M.C., Abramson,M.J., Allen,K.J. & Dharmage,S.C. 2011. House dust mite sensitization in toddlers predicts current wheeze at age 12 years. J Allergy Clin Immunol., 128, 782-788.

Massie,T. Statistical criteria for establishing safety and efficacy of allergenic products. Allergenics Products Advisory Committee, U. S. Food and Drug Administration. 2011.
Ref Type: Slide

Masuyama,K., Okamoto,Y., Okamiya,K., Azuma,R., Fujinami,T., Riis,B., Ohashi,K., Natsui,K., Imai,T. & Okubo,K. 2018. Efficacy and safety of SQ house dust mite sublingual immunotherapy-tablet in Japanese children. Allergy.

Matsuoka,T., Bernstein,D.I., Masuyama,K., Nolte,H., Okamiya,K., Seitzberg,D. & Nelson,H.S. 2017. Pooled efficacy and safety data for house dust mite sublingual immunotherapy tablets in adolescents. Pediatr Allergy Immunol, 28, 661-667.

Meltzer,E.O., Blaiss,M.S., Derebery,M.J., Mahr,T.A., Gordon,B.R., Sheth,K.K., Simmons,A.L., Wingertzahn,M.A. & Boyle,J.M. 2009. Burden of allergic rhinitis: results from the Pediatric Allergies in America survey. J Allergy Clin Immunol, 124, S43-S70.

Mir,E., Panjabi,C. & Shah,A. 2012. Impact of allergic rhinitis in school going children. Asia Pac. Allergy, 2, 93-100.

Nathan,R.A. 2007. The burden of allergic rhinitis. Allergy Asthma Proc., 28, 3-9.

Nolte,H., Bernstein,D.I., Nelson,H.S., Kleine-Tebbe,J., Sussman,G.L., Seitzberg,D., Rehm,D., Kaur,A., Li,Z. & Lu,S. 2016. Efficacy of house dust mite sublingual immunotherapy tablet in North American adolescents and adults in a randomized, placebo-controlled trial. J Allergy Clin Immunol, 138, 1631-1638.

Nolte,H., Maloney,J., Nelson,H.S., Bernstein,D.I., Lu,S., Li,Z., Kaur,A., Zieglmayer,P., Zieglmayer,R., Lemell,P. & Horak,F. 2015. Onset and dose-related efficacy of house dust mite sublingual immunotherapy tablets in an environmental exposure chamber. J Allergy Clin Immunol, 135, 1494-1501.

Novembre,E., Galli,E., Landi,F., Caffarelli,C., Pifferi,M., De,M.E., Burastero,S.E., Calori,G., Benetti,L., Bonazza,P., Puccinelli,P., Parmiani,S., Bernardini,R. & Vierucci,A. 2004. Coseasonal

sublingual immunotherapy reduces the development of asthma in children with allergic rhinoconjunctivitis. J. Allergy Clin. Immunol., 114, 851-857.

Okubo,K., Masuyama,K., Imai,T., Okamiya,K., Stage,B.S., Seitzberg,D. & Konno,A. 2016. Efficacy and safety of the SQ house dust mite sublingual immunotherapy tablet in Japanese adults and adolescents with house dust mite-induced allergic rhinitis. J Allergy Clin Immunol.

Passalacqua,G., Baena-Cagnani,C.E., Bousquet,J., Canonica,G.W., Casale,T.B., Cox,L., Durham,S.R., Larenas-Linnemann,D., Ledford,D., Pawankar,R., Potter,P., Rosario,N., Wallace,D. & Lockey,R.F. 2013. Grading local side effects of sublingual immunotherapy for respiratory allergy: Speaking the same language. J Allergy Clin Immunol, 132, 93-98.

Pfaar,O., Demoly,P., Gerth van,W.R., Bonini,S., Bousquet,J., Canonica,G.W., Durham,S.R., Jacobsen,L., Malling,H.J., Mosges,R., Papadopoulos,N.G., Rak,S., Rodriguez Del,R.P., Valovirta,E., Wahn,U. & Calderon,M.A. 2014. Recommendations for the standardization of clinical outcomes used in allergen immunotherapy trials for allergic rhinoconjunctivitis: an EAACI Position Paper. Allergy..

Reddel,H.K., Taylor,D.R., Bateman,E.D., Boulet,L.P., Boushey,H.A., Busse,W.W., Casale,T.B., Chanez,P., Enright,P.L., Gibson,P.G., de Jongste,J.C., Kerstjens,H.A., Lazarus,S.C., Levy,M.L., O'Byrne,P.M., Partridge,M.R., Pavord,I.D., Sears,M.R., Sterk,P.J., Stoloff,S.W., Sullivan,S.D., Szefler,S.J., Thomas,M.D. & Wenzel,S.E. 2009. An official American Thoracic Society/European Respiratory Society statement: asthma control and exacerbations: standardizing endpoints for clinical asthma trials and clinical practice. Am. J. Respir. Crit Care Med., 180, 59-99.

Salmun,L.M. & Lorber,R. 2002. 24-hour efficacy of once-daily desloratadine therapy in patients with seasonal allergic rhinitis [ISRCTN32042139]. BMC. Fam. Pract., 3, 14.

Sampson,H.A., Munoz-Furlong,A., Campbell,R.L., Adkinson,N.F., Jr., Bock,S.A., Branum,A., Brown,S.G., Camargo,C.A., Jr., Cydulka,R., Galli,S.J., Gidudu,J., Gruchalla,R.S., Harlor,A.D., Jr., Hepner,D.L., Lewis,L.M., Lieberman,P.L., Metcalfe,D.D., O'Connor,R., Muraro,A., Rudman,A., Schmitt,C., Scherrer,D., Simons,F.E., Thomas,S., Wood,J.P. & Decker,W.W. 2006. Second symposium on the definition and management of anaphylaxis: summary report--Second National Institute of Allergy and Infectious Disease/Food Allergy and Anaphylaxis Network symposium. J Allergy Clin Immunol, 117, 391-397.

Sears,M.R., Herbison,G.P., Holdaway,M.D., Hewitt,C.J., Flannery,E.M. & Silva,P.A. 1989. The relative risks of sensitivity to grass pollen, house dust mite and cat dander in the development of childhood asthma. Clin Exp Allergy, 19, 419-424.

Sporik,R., Holgate,S.T., Platts-Mills,T.A. & Cogswell,J.J. 1990. Exposure to house-dust mite allergen (Der p I) and the development of asthma in childhood. A prospective study. N. Engl. J Med., 323, 502-507.

Terreehorst,I., Oosting,A.J., Tempels-Pavlica,Z., De Monchy,J.G., Bruijnzeel-Koomen,C.A., Hak,E. & van Wijk,R.G. 2002. Prevalence and severity of allergic rhinitis in house dust mite-allergic patients with bronchial asthma or atopic dermatitis. Clin Exp. Allergy, 32, 1160-1165.

Valovirta,E., Myrseth,S.E. & Palkonen,S. 2008. The voice of the patients: allergic rhinitis is not a trivial disease. Curr. Opin. Allergy Clin Immunol, 8, 1-9.

van Cauwenberge,P., Bachert,C., Passalacqua,G., Bousquet,J., Canonica,G.W., Durham,S.R., Fokkens,W.J., Howarth,P.H., Lund,V., Malling,H.J., Mygind,N., Passali,D., Scadding,G.K. & Wang,D.Y. 2000. Consensus statement on the treatment of allergic rhinitis. European Academy of Allergology and Clinical Immunology. Allergy, 55, 116-134.

Virchow,J.C., Backer,V., Kuna,P., Prieto,L., Nolte,H., Villesen,H.H., Ljorring,C., Riis,B. & de,B.F. 2016. Efficacy of a House Dust Mite Sublingual Allergen Immunotherapy Tablet in Adults With Allergic Asthma: A Randomized Clinical Trial. JAMA., 315, 1715-1725.

World Medical Association. Declaration of Helsinki - Ethical Principles for Medical Research Involving Human Subjects. 2013. Adopted by the WMA General Assembly in Helsinki (1964) and as amended by the WMA General Assembly.

Ref Type: Report

Figure S1: Trial design

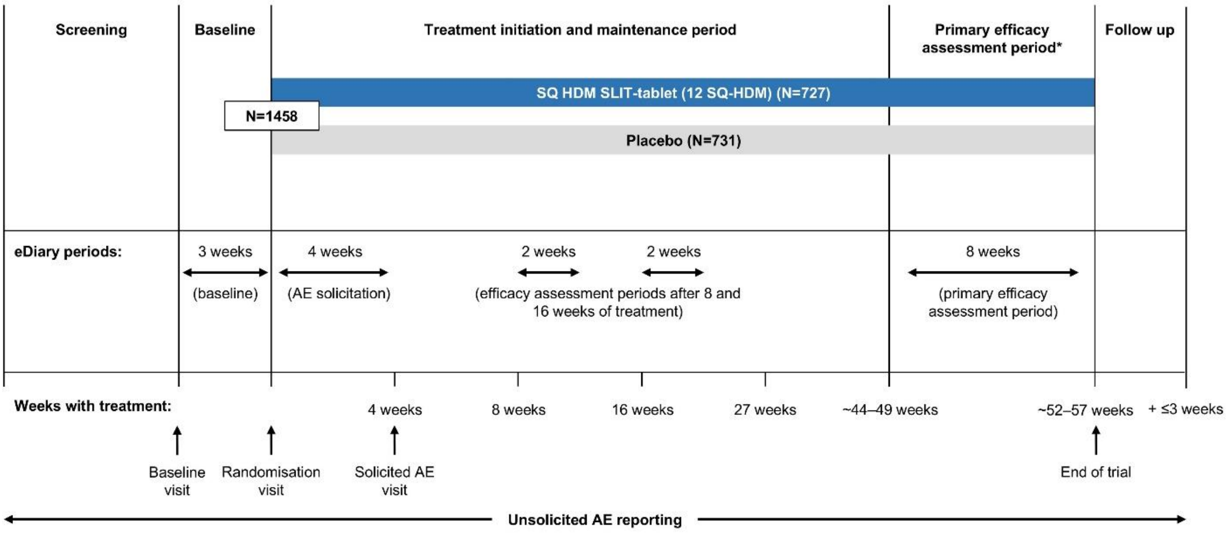

The date of the first subject first visit was 12 October 2019, and of the last subject last visit was 21 April 2023. *The primary efficacy assessment period had to fall between 1 September and 1 April and include the dates of the 3-week baseline period from the previous year. For pollen allergic subjects, the primary efficacy assessment period had to be outside of their pollen allergy season. AE=adverse event. HDM=house dust mite. SLIT=sublingual immunotherapy.

The Paediatric Rhinoconjunctivitis Quality of Life Questionnaire (PRQLQ)

The PRQLQ is a questionnaire that evaluates the quality of life of children with ARC. The PRQLQ is composed of 23 questions, divided into five domains (see table below).

Individual domains and associated questions comprising the PRQLQ

Domain	Items	Score card
**Nose symptoms**	How much were you bothered by a stuffy, blocked nose during the past week?	Blue
	How much were you bothered by sneezing during the past week?	
	How much were you bothered by a runny nose during the past week?	
	How much were you bothered by an itchy nose during the past week?	
**Eye symptoms**	How much were you bothered by itchy eyes during the past week?	Blue
	How much were you bothered by watery eyes during the past week?	
	How much were you bothered by swollen/puffy eyes during the past week?	
	How much were you bothered by sore eyes during the past week?	
**Practical problems**	How much were you bothered by having to rub your eyes and nose during the past week?	Blue
	How much were you bothered by having to blow your nose during the past week?	
	How much were you bothered by having to carry Kleenex during the past week?	
	How much were you bothered by having to take medications for your allergies during the past week?	
	How often did your allergies make you feel embarrassed during the past week?	Green
**Other symptoms**	How much were you bothered by thirst during the past week?	Blue
	How much were you bothered by scratchy/itchy throat during the past week?	Blue
	How much were you bothered by having a headache during the past week?	Blue
	How often did your allergies make you feel tired during the past week?	Green
	How often did your allergies make you feel not well all over during the past week?	Green
	How often did your allergies make you feel irritable (cranky/grouchy) during the past week?	Green
**Activity limitation**	How much were you bothered by your allergies playing outdoors during the past week?	Blue
	How often did your allergies make it hard to get to sleep during the past week?	Green
	How often did your allergies wake you up during the night during the past week?	Green
	How often did your allergies make it hard to pay attention during the past week?	Green

Each question is answered on a 7-point scale, ranging from 0 to 6 according to how often the child experiences symptoms or how bothersome the symptoms are to the child (see table below).

Scoring system for the PRQLQ

Scoring	Explanation for the green card	Explanation for the blue card
**0**	None of the time	Not bothered
**1**	Hardly any of the time	Hardly bothered at all
**2**	Once in a while	Bothered a bit
**3**	Some of the time	Somewhat bothered
**4**	Quite often	Quite bothered
**5**	Most of the time	Very bothered
**6**	All of the time	Extremely bothered

The overall PRQLQ score is calculated as an average score for all 23 questions. The domain score is calculated as the mean score for the questions in the relevant domain. The higher the overall score, the lower the quality of life of the child.

Definition of an event of special interest (ESI)

ESIs are events considered critical for the evaluation of the safety profile for the SQ HDM SLIT-tablet, and additional data on these events were collected. An ESI can be any of the following:

- anaphylactic reactions and/or systemic allergic reactions. (See the clinical trial protocol for the definition of anaphylaxis)
- events treated with adrenaline/epinephrine
- severe local swelling or oedema of the mouth and/or throat
- eosinophilic oesophagitis.

Statistical analysis plan

16.1.9 Documentation of statistical method

Statistical Analysis Plan

Trial ID: MT-12

A one-year placebo-controlled phase III trial evaluating the efficacy and safety of the house dust mite (HDM) SLIT-tablet in children (5-11 years of age) with HDM allergic rhinitis/rhinoconjunctivitis with or without asthma

Sponsor: ALK-Abelló A/S
Bøge Alle 6-8
DK-2970 Hørsholm

Investigational medicinal product: HDM SLIT-tablet

Phase: III

EudraCT No.: 2019-000560-22

Document status: Final

Date: 24-MAY-2023

Version number: 1.0

Property of ALK
May not be used, divulged, published, or otherwise disclosed without the written consent of ALK

ALK approval of statistical analysis plan

Trial statistician:

[redacted]

Signature

Date

Trial manager:

[redacted]

Signature

Date

Medical writer:

[redacted]

Signature

Date

Head of biometrics:

[redacted]

Signature

Date

Head of GPCD:

[redacted]

Signature

Date

This document is approved by electronic signature, refer to signature page at the end of this document.

Table of contents

Page
ALK approval of statistical analysis plan 2
Table of contents 4
Table of figures 7
Table of tables 7
1 List of abbreviations 8
2 List of definitions 9
3 Introduction 10
3.1 Objectives, endpoints, and estimands 10
3.1.1 Estimands 12
3.1.1.1 Population 12
3.1.1.2 Population level summary 12
3.1.1.3 Variable 12
3.1.1.4 Intercurrent events 12
3.1.1.4.1 Hypothetical strategy 13
3.1.1.4.2 Treatment policy strategy 13
3.2 Study design 13
3.2.1 Flow chart 15
3.2.2 Randomisation 19
4 Statistical hypotheses 19
4.1.1 Multiplicity adjustment 19
5 Analysis sets 19
6 Statistical analyses 20
6.1 General considerations 20
6.1.1 Potential data issues 21
6.2 Primary endpoint analyses 21
6.2.1 Definition of endpoint 21
6.2.2 Primary analysis 21
6.2.3 Applying the estimand framework 22
6.2.3.1 Trial product estimand 22
6.2.3.1.1 Main analytical approach 22
6.2.3.1.2 Sensitivity analytical approach 23
6.2.3.2 Treatment policy estimand 24
6.2.3.2.1 Main analytical approach 24
6.2.3.2.2 Sensitivity analytical approach 24
6.3 Key secondary endpoint analyses 24
6.3.1 Definition of endpoints 24

6.3.2 Primary analysis 24
6.3.3 Applying the estimand framework 25
6.4 Summary of primary and key secondary endpoint analyses 25
6.5 Additional secondary efficacy endpoint analyses 26
6.5.1 Immunology 27
6.6 Explorative endpoint analyses 27
6.7 Safety analyses 28
6.7.1 Extent of exposure 28
6.7.1.1 Treatment duration 28
6.7.1.2 Exposure 28
6.7.1.3 IMP compliance 28
6.7.1.4 eDiary compliance for TCRS 28
6.7.2 Adverse events 29
6.7.2.1 Treatment emergent adverse events 29
6.7.2.2 Solicited AEs 29
6.7.2.3 Events of special interest 30
6.7.2.4 Reporting of AEs 30
6.7.3 Additional safety assessments 31
6.8 Subgroup analyses 31
6.9 Interim analysis 31
6.10 Changes and/or deviations to protocol-planned analyses 31
7 Sample size determination 32
7.1 Assumptions 32
7.2 Results of sample size calculation 33
8 Demographics and baseline characteristics 34
8.1 Screening failures 34
8.2 Protocol deviations 34
8.3 Subject disposition 34
8.4 Baseline characteristics 34
8.5 Medical history 34
8.6 Prior and concomitant therapy 34
9 Supporting documentation 35
9.1 Derivations for the study 35
9.1.1 Derivation of daily symptom and medication scores 35
9.1.1.1 Rhinitis DSS 35
9.1.1.2 Conjunctivitis DSS 35

9.1.1.3 Rhinoconjunctivitis DSS 35
9.1.1.4 Rhinitis DMS 35
9.1.1.5 Conjunctivitis DMS 36
9.1.1.6 Rhinoconjunctivitis DMS 37
9.1.1.7 TCRS 37
9.1.1.8 TCCS 37
9.1.1.9 TCS 37
9.1.1.10 Asthma DSS 37
9.1.1.11 Rhinitis CSMS 37
9.1.1.12 Rhinoconjunctivitis CSMS 38
9.1.2 Medication scores used for sensitivity analyses 38
9.1.2.1 Rhinitis DMS_sens 39
9.1.2.2 Conjunctivitis DMS_sens 39
9.1.2.3 TCRS_sens 40
9.1.2.4 TCS_sens 40
9.1.3 Derivation of average symptom and medication scores 41
9.1.4 Derivation of daily binary variable 41
9.1.4.1 Rhinitis mild day 41
9.1.4.2 Rhinitis exacerbation day 42
9.1.4.3 SABA free day 42
9.1.4.4 Rhinitis symptom-free day 42
9.1.4.5 Nocturnal awakenings due to asthma requiring SABA use 42
9.1.5 Other derivations of endpoints 42
9.1.5.1 Average weekly number of puffs of as-needed SABA use 42
9.1.5.2 PRQLQ 42
9.1.5.3 Absence from school/work for a subject and parent/caregiver 42
9.1.5.4 Health care utilisation 43
9.1.6 Imputation of dates 43
9.1.6.1 Partial dates in AE reporting 43
9.1.6.2 Incomplete date for last IMP 43
9.1.6.3 Imputation for date of birth 43
9.1.7 Other derivations 44
9.1.7.1 Subjects with reported asthma 44
9.1.7.2 eDiary periods 44
9.1.7.3 Age for DMS 44
9.2 Further details pertaining to statistical analyses 44

9.2.1.3 Square root transformation 46
9.2.1.4 Mean and SE 46
9.3 Subjects excluded from the sensitivity analysis 47
10 References 48

Table of figures

Page

Figure 1 Study Design 14

Table of tables

Page

Table 1 Schedule of cohorts 15
Table 2 Flow chart 15
Table 3 Overview of analyses for the primary and key secondary endpoints 25
Table 4 Correspondance between solicited symptom/sign and PT 29
Table 5 Mean and standard deviation of TCRS by treatment group in MT-06 32
Table 6 Effect size of TCRS in MT-06, P001, and TO-203-3-3 33
Table 7 Power calculations based on different assumptions of the effect size using observed case approach 33
Table 8 Rhinitis DMS scoring 35
Table 9 Conjunctivitis DMS Scoring 36
Table 10 Rhinitis Medication Scoring for CSMS by EAACI task force 37
Table 11 Rhinoconjunctivitis medication scoring for CSMS by EAACI task force 38
Table 12 Rhinitis DMS_sens scoring 39
Table 13 Conjunctivitis DMS_sens scoring 40
Table 14 SAS code for primary efficacy analysis 46
Table 15 SAS code for additional efficacy analysis (binary endpoint) 47

Version History

This statistical analysis plan for trial MT-12 is based on the protocol version 5.0 dated 19-Mar-2021.

SAP Version	***Date***	***Change***	***Rationale***
1	24-MAY-2023	Not applicable	Original version

1 List of abbreviations

AE	Adverse event
AR	Allergic rhinitis
ANOVA	Analysis of variance
CI	Confidence interval
CRF	Case report form
CSMS	Combined symptom and medication score (recommended by European academy of allergy and clinical immunology task force)
CSR	Clinical study report
DMS	Daily medication score
DPS	Data points set
DSS	Daily symptom score
ESI	Event of special interest
EAACI	European academy of allergy and clinical immunology
EudraCT	European Union drug regulating authorities clinical trials database
FAS	Full analysis set
FDA	Food and Drug Administration
FEV_1	Forced expiratory volume in 1 second
FVC	Forced vital capacity
GCP	Good clinical practice
GLMM	General linear mixed model
HDM	House dust mite
ICH	International council for harmonisation of technical requirements for pharmaceuticals for human use
ICS	Inhaled corticosteroid
ID	Identification
IgE	Immunoglobulin E

IMP	Investigational medicinal product
ITT	Intention-to-treat
LME	Linear mixed effects model
MAR	Missing at random
MedDRA	Medical dictionary for regulatory activities
MMRM	Mixed-effect model for repeated measurements
MNAR	Missing not at random
PRQLQ	Paediatric rhinoconjunctivitis quality of life questionnaire
PD	Protocol deviation
PT	Preferred term
QoL	Quality of life
SABA	Short-acting β_2-agonist
SAE	Serious adverse event
SAF	Safety analysis set
SAP	Statistical analysis plan
SAS	Statistical analysis software
SD	Standard deviation
SE	Standard error
SOC	System organ class
SPT	Skin prick test
SLIT	Sublingual allergy immunotherapy
TC FU	Telephone follow-up
TCCS	Total combined conjunctivitis score
TCRS	Total combined rhinitis score
TCS	Total combined score (of rhinoconjunctivitis symptoms and medication)
TEAE	Treatment-emergent adverse event
WHO	World Health Organisation

2 List of definitions

AE	An AE is any untoward medical occurrence in a clinical trial subject and which does not necessarily have a causal relationship with the administered IMP
Completed	A randomised subject is considered as completed if he/she has not discontinued

subject	the trial
Concomitant medication	All medications being continued by a subject on entry into the trial and all medications given in addition to IMP and rescue medication
IMP	A pharmaceutical form of the active substance or placebo being tested
Solicited AE	A pre-specified AE recorded by the investigator based on the symptoms reported in the subject's eDiary during the first 28 days after randomisation
Source documents	Source documents are original documents, data, and records from which the subjects' eCRF data are obtained. These include, but are not limited to, hospital records (from which medical history and concomitant medication may be summarised into the eCRF), clinical and office charts, laboratory and pharmacy records, diaries, microfiches, radiographs, and correspondence
Trial completion	The trial is completed once the CSR is signed
Treatment-emergent AE	An AE that starts on or after the first IMP administration and no later than 7 days after the last IMP administration

3 Introduction

The statistical analysis plan is a supplement to the protocol and provides additional details regarding estimands (where appliable), analysis sets, endpoints and the statistical analyses.

Supporting documentation is provided in section 9. Section 9.1 'Derivations for the study' contains details about the derivations used in the study. In Section 9.2 'Further details pertaining to statistical analyses', a detailed description of how to code the statistical analyses is provided, and in section 9.3 'Subjects excluded from the sensitivity analysis' a list of subjects to be excluded from the sensitivity analysis is given.

Changes to the analyses and changes that could impact the analyses described in the protocol are documented in section 6.10.

This statistical analysis plan has been written and approved before database lock and unblinding.

3.1 Objectives, endpoints, and estimands

**Objectives**	**Endpoints**
Primary objective	Primary endpoints
Demonstrate the efficacy of the HDM SLIT-tablet compared to placebo in the treatment of HDM AR in children (5-11 years of age) based	• The average daily TCRS during the primary efficacy assessment period

on total combined rhinitis symptoms and medication use during the primary efficacy assesment period.	
Key secondary objectives	Key secondary endpoints
Demonstrate the efficacy of the HDM SLIT-tablet compared to placebo during the primary efficacy assessment period based on rhinitis symptoms, rhinitis medication use, combined rhinoconjunctivitis symptoms and medication use, and rhinoconjunctivitis QoL.	• The average rhinitis DSS during the primary efficacy assessment period<br>• The average rhinitis DMS during the primary efficacy assessment period<br>• The average daily TCS during the primary efficacy assessment period
Secondary objectives	Secondary endpoints
Evaluate the HDM SLIT-tablet compared to placebo during the primary efficacy assessment period based on safety and tolerability, rhinoconjunctivitis symptoms, rhinoconjunctivitis medication use, rhinoconjunctivitis QoL, asthma symptoms and medication use, changes in immunological parameters, rhinitis mild days, rhinitis exacerbation days, rhinitis CSMS, rhinoconjunctivitis CSMS	• Safety and tolerability assessments<br>• Average rhinoconjunctivitis DSS<br>• Average rhinoconjunctivitis DMS<br>• PRQLQ score<br>• Average asthma DSS<br>• SABA free days<br>• Weekly number of puffs of as-needed SABA use<br>• Changes in immunological parameters<br>• Rhinitis mild days<br>• Rhinitis exacerbation days<br>• Average daily rhinitis CSMS (recommended by the EAACI)<br>• Average daily rhinoconjunctivitis CSMS (recommended by the EACCI)
Explorative objectives	Explorative endpoints
Evaluate the following: combined rhinitis symptoms and medication use during pollen season in spring and summer, conjunctivitis symptoms, conjunctivitis medication use and combined conjunctivitis symptoms and medication use during the primary efficacy assessment period, rhinitis combined symptoms and medication use after 8 and 16 weeks of treatment, rhinoconjunctivitis combined symptoms and medication use after 8 and 16 weeks of treatment, nocturnal	• Average daily TCRS during pollen season in spring and summer<br>• Average onjunctivitis DSS, average conjunctivitis DMS, and average daily TCCS (combined conjunctivitis symptoms and medication use) during the primary efficacy assessment period<br>• Average daily TCRS, average daily

awakenings due to asthma requiring SABA use, absence from school/work for a subject and parent/caregiver, health care utilisation	TCS, average rhinitis DSS, average rhinitis DMS during the 2 weeks period collected 8 and 16 weeks after treatment initiation<br>• Rhinitis symptom-free days<br>• Nocturnal awakenings due to asthma requiring SABA use<br>• Absence from school/work for a subject and parent/caregiver<br>• Health care utilisation

3.1.1 Estimands

The study was not designed using the estimand framework, but since the first version of the protocol the ICH E9 addendum (ICH 2019, 1998) has come into affect. Therefore the primary analysis using an observed case analysis is supplemented with analyses of the trial product estimand and the treatment policy estimand using the estimand framework.

For all estimands considered for the study, the population, the population-level summary, and the intercurrent events are given below.

3.1.1.1 Population

The population is defined by the inclusion and exclusion criteria of the trial to reflect the targeted patient population for approval.

3.1.1.2 Population level summary

The absolute difference in means between placebo and HDM SLIT-tablet.

3.1.1.3 Variable

The two estimands will be defined using the primary endpoint average TCRS, and the key secondary endpoints average rhinitis DSS, average rhinitis DMS, and average daily TCS, all during the primary efficacy assessment period.

3.1.1.4 Intercurrent events

In allergy immunotherapy clinical trials, use of rescue medication is allowed according to the protocol and is included in the treatment condition. Treatment interruptions are also considered part of the treatment condition. Discontinuation of IMP is considered an intercurrent event and will be handled by different strategies for the estimands considered for the study, either the hypothetical strategy, or the treatment policy strategy.

3.1.1.4.1 Hypothetical strategy

The intercurrent event of discontinuation of treatment will be handled by the **hypothetical strategy**, i.e. if a subject discontinues treatment, only diary data up until the time of treatment discontinuation will be included in the analysis, and any data recorded after discontinuation of treatment will be excluded from the analysis.

Thus, an estimand applying the hypothetical strategy is the absolute difference between placebo and HDM SLIT-tablet based on the endpoint, in the population defined by the trial inclusion and exclusion criteria, if all subjects adhered to the treatment regimen. In other words, the estimand is the expected treatment effect in subjects with HDM allergic rhinitis/rhinoconjunctivitis if all subjects adhered to the treatment regimen.

A such estimand is denoted a **trial product estimand**, as it assesses the anticipated effect of the treatment (trial product) if taken as instructed. This is considered to be of most relevance to the patient, as it describes the potential benefit they could obtain from the trial product if they adhere to treatment for the planned duration.

3.1.1.4.2 Treatment policy strategy

The intercurrent event of discontinuation of treatment, will be handled by the **treatment policy strategy**, i.e. all collected data also after the time of treatment discontinuation will be included in the analysis.

Thus, an estimand applying the treatment policy strategy is the absolute difference between placebo and HDM SLIT-tablet based on the endpoint, in the population defined by the trial inclusion and exclusion criteria, regardless of whether subjects complete treatment for the planned duration. In other words, the estimand is the effect of the treatment regimen in subjects with HDM allergic rhinitis/rhinoconjunctivitis regardless of whether the subject adhered to the treatment regimen.

A such estimand is denoted a **treatment policy estimand**, as it assesses the treatment effect regardless of adherence to treatment and provides a broad perspective of the treatment effect in clinical practice.

3.2 Study design

This trial is a randomised, parallel-group, double-blind, placebo-controlled, multinational phase III trial conducted in Europe and North America.

The trial was initiated on 10-OCT-2019 and three cohorts were included. Subjects received treatment for approximately 12 months.

It was planned that approximately 1370 subjects would be randomised (1:1) to receive treatment with the HDM SLIT-tablet or placebo.

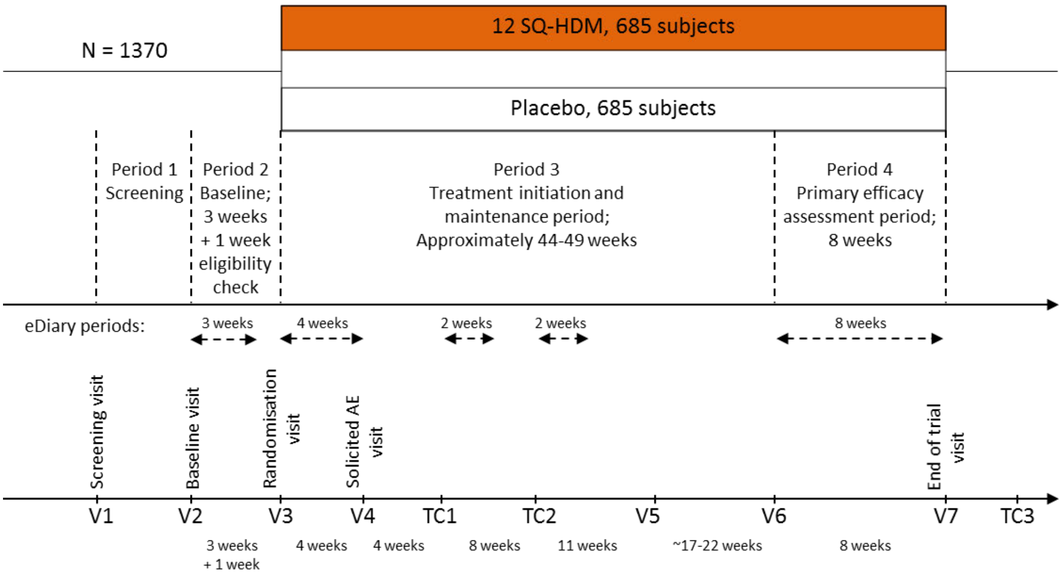

Figure 1 Study Design

The trial consisted of 4 periods: screening (Period 1), a baseline period (Period 2), a treatment initiation and maintenance period (Period 3), and a primary efficacy assessment period during the last 8 weeks (period 4) of approximately 12 months treatment.

Period 1 was the screening period. Subjects may have been screened up to 12 weeks before randomisation.

Period 2 was the baseline period and lasted 21 days plus 7 days for eligibility check. Subjects' baseline must have been recorded between 1 September and 1 April. In addition, pollen allergic subjects must have their baseline period outside the season of their pollen allergy. During the baseline period, subjects were allowed to take sponsor provided rhinoconjunctivitis and asthma rescue medication, and their rhinoconjunctivitis symptoms and use of rescue medication were scored on a daily basis in their eDiary. To be eligible for inclusion, subjects were required to have a rhinitis DSS of at least 6, or a score of at least 5 with one symptom being severe, on 8 of the last 14 days of the baseline period and were required to use rescue medication for the treatment of HDM allergic rhinitis during at least 8 of the last 14 days of the baseline period. The baseline period must have ended 1 week before randomisation to allow for eligibility check. Subjects with evidence of current, clinically significant, intercurrent illness (e.g., significant common cold or influenza) during the baseline period could be rescheduled for a repeat of the baseline on a resolution of their illness. The blood samples for safety testing were repeated if the results were more than 12 weeks old.

Period 3 was the treatment initiation and maintenance period. It began at V3 (randomisation visit) and lasted until V6 (44-49 weeks of treatment). During the first 28 days of period 3, 15 pre-specified symptoms/signs (based on recommendation by the WAO (Passalacqua et al. 2013) occurring after IMP intake were solicited and recorded by the parent/caregiver together with the subject in the eDiary. If pre-specified symptoms were reported in the eDiary, the investigator was to evaluate these and report them in the eCRF as solicited AEs at V4 (4 weeks of treatment). To evaluate efficacy, there were two 2 weeks efficacy assessment periods recorded in the eDiary at TC1 (after 8 weeks of treatment) and TC2 (after 16 weeks of treatment).

Period 4 was the 8-week primary efficacy assessment period. It began at V6 (44-49 weeks of treatment) and lasted until V7(end of trial). Subjects' primary efficacy assessment period had to be between 1 September and 1 April and the period was planned to include the dates that were included in the 3-week baseline period the previous year. In addition, pollen allergic subjects must have had their primary efficacy assessment period outside the season of their pollen allergy. During period 4, the parent/caregiver together with the subject rated the rhinoconjunctivitis symptoms and use of rescue medication on a daily basis in the eDiary.

The schedule of cohorts is presented in Table 1.

Table 1 Schedule of cohorts

	**Cohort 1**	**Cohort 2**	**Cohort 3**
**First subject first visit (FSFV)**	12-Oct-2019	07-Jul-2020	07-Jul-2021
**Last subject randomised**	20-Mar-2020	01-Apr-2021	01-Apr-2022
**Last subject last visit**	On-site (V7): 08-Jul-2021<br>Telephone (TC3): 22-Jul-2021	On-site (V7): 07-Apr-2022<br>Telephone (TC3): 25-Apr-2022	On-site (V7): 07-Apr-2023<br>Telephone (TC3): 21-Apr-2023

3.2.1 Flow chart

The flow chart of the study assessments by visits are shown below in

Table 2.

Table 2 Flow chart

Visit ID:	V1	V2	V3	V4	TC1	TC2	V5	V6	V7	TC 3	UV
Visit	Screening	Base-line	Randomi-sation	Solicited AEs					End of trial	Follow-up	Unschedul-ed visit
Time from randomisation (IMP initiation)	Max -12 weeks	-4 weeks<br>-7 d		8<br>+ 7 d	8 weeks<br>± 7 d	16 weeks<br>± 7 d	27 weeks<br>± 7 d	44-49 weeks<br>± 7 d	52-57 weeks<br>± 7 d[1]	+ 2 weeks from V7 + 7 d	
Informed consent	X										
Demography	X										
Medical history	X										

[1] V7 to be performed 8 weeks +14 days after V6 and no later than 1st April

Visit ID:	V1	V2	V3	V4	TC1	TC2	V5	V6	V7	TC 3	UV
Assess symptoms of eosinophilic oesophagitis	X	X	X	X	X	X	X	X	X	X	(X)
Record previous and concomitant medication	X	X	X	X	X	X	X	X	X	X	X
Physical examination	X								X		(X)
Oropharyngeal examination		X	X[2]	X			X	X			(X)
Height and weight[3]	X	X	X	X			X	X	X		X
Vital signs	X	X	X	X			X	X	X		(X)
Body temperature			X								
FEV_1			X						X		(X)
Urine pregnancy test, if applicable[4]	X	X	X	X			X	X	X		(X)
SPT	X	(X)[5]									
In-/exclusion criteria	X	X	X								
Blood and urine samples for safety laboratory assessments	X								X		(X)
Blood sample for specific IgE[6]	X[7]										(X)
Assess and record Aes in eCRF	X	X	X	X	X	X	X	X	X	X	X
Randomisation			X								
Issue and review local and systemic allergic reaction emergency plan			X								
PRQLQ			X				X	X	X		

[2] Oropharyngeal examinations was done before and 30 ±5 mins after IMP administration at Visit 3

[3] If applicable, adjust the local and systemic allergic reaction emergency plan

[4] For female subjects of childbearing potential

[5] SPT to be performed at visit 2, if not possible at visit 1, due to necessary wash-out of concomitant medication

[6] IgE against *D pteronyssinus and D farinae*

[7] Inform subjects that continued participation in trial depends on the result of the blood sample for specific IgE against *D. pteronyssinus* and *D. farinae*

Visit ID:	V1	V2	V3	V4	TC1	TC2	V5	V6	V7	TC 3	UV
Record absence from school/work for subject and parent/caregiver			X	X	X	X	X	X	X		(X)
Record health care utilisation			X	X	X	X	X	X	X		(X)
Blood sample for pharmacogenetics biobank[8]									X		
Intake of IMP at clinic			X								(X)
Dispense IMP			X	X			X	X			(X)
Dispense rhinoconjunctivitis and asthma rescue medication and instruct in the use		X	X	X			X	X			(X)
Dispense adrenaline/ epinephrine auto-injectors[9]			X	X			X	X			(X)
Collect unused adrenaline/ epinephrine auto-injector									X		
Collect rescue medication as applicable and perform drug accountability			X	X			X	X	X		
Collect IMP, perform drug accountability and IMP compliance check				X			X	X	X		
Show and discuss trial video		X						X			
Issue and instruct parent/caregiver in use and activation of eDiary		X	X		X	X		X			X
Instruct in the recording of pre-specified symptoms in eDiary			X								

[8] For subjects where the subject/parent/guardian has given consent
[9] For countries where this is a regulatory requirement

Visit ID:	V1	V2	V3	V4	TC1	TC2	V5	V6	V7	TC 3	UV
Review eDiary and record solicited Aes in eCRF				X							
eDiary recording		3 weeks	4 weeks		2 weeks	2 weeks		8 weeks			
Check eDiary compliance			X	X					X		
Collect eDiary									X		

For Canada and Poland only:

Visit ID:	V1	V2	V3	V4	TC1	TC2	V5	V6	V7	TC 3	UV
Blood sample for immunological assessments	X								X		
Biobank blood sample[10]	X								X		

10 For subjects where the subject/parent/guardian has given consent

3.2.2 Randomisation

The randomisation list was generated by a trial-independent statistician and was not accessible to trial personnel involved in the conduct of the trial until the database had been locked.

When a subject was randomised a unique number was assigned at the time of the first dispensing of IMP.

4 Statistical hypotheses

4.1.1 Multiplicity adjustment

The primary endpoint, key secondary endpoints, and the overall PRQLQ score at end of trial (using observed case analysis) will be controlled for multiplicity to ensure a maximum overall type I error rate of 5% in the hypothesis testing of these endpoints. The control for multiplicity is done by hierarchical testing, pre-specifying the order of the hypotheses to be tested. For all endpoints the null hypothesis to be tested is the hypothesis of no absolute difference in means between treatment groups. Let μ_1 denote the mean in the HDM SLIT-tablet group, and μ_2 denote the mean in the Placebo group. Then the null hypothesis (H_0) and the alternative hypothesis (H_A) are given as follows:

H_0: $\mu_1 = \mu_2$ and H_A: $\mu_1 \neq \mu_2$

The order of hypotheses to be tested is:

1. Superiority testing of the HDM SLIT-tablet over placebo with respect to the average daily TCRS during the primary efficacy assessment period.
2. Superiority testing of the HDM SLIT-tablet over placebo with respect to the average rhinitis DSS during the primary efficacy assessment period.
3. Superiority testing of the HDM SLIT-tablet over placebo with respect to the average rhinitis DMS during the primary efficacy assessment period.
4. Superiority testing of the HDM SLIT-tablet over placebo with respect to the average daily TCS during the primary efficacy assessment period.
5. Superiority testing of the HDM SLIT-tablet over placebo with respect to the overall PRQLQ score at end of trial.

All hypotheses in the hierarchy are tested on a 5% significance level. If the first hypothesis is statistically significant at the 5% level i.e., the null hypothesis of no difference is rejected, then the second hypothesis is tested, and so forth. A hypothesis is only tested if all previously tested hypothesis were statistically significant at the 5% level i.e., all previous null hypotheses were rejected.

5 Analysis sets

For the purposes of analysis, the following analysis sets are defined:

Analysis Set	Description
**Total analysis set**	The total analysis set comprises all subjects who signed informed consent and thus includes screening failures.
**Full analysis set (FAS)**	The FAS consists of all randomised subjects who received at least one dose of IMP.

	Subjects will be analysed as randomised i.e., according to their randomised assignment of treatment.
**Safety analysis set (SAF)**	The SAF consists of all randomised subjects, who received at least one dose of IMP. Subjects will be analysed as treated i.e., according to treatment they actually received.

The Total Analysis Set will be used for listing reasons for screening failure and for AEs before randomisation. The FAS will be used for baseline tables, efficacy and questionnaire endpoints, and the SAF will be used for safety reporting. FAS and all observed data for the endpoint is used for the observed case analyses (primary analysis for all endpoints).

The following data point sets (DPS) are defined:

DPS	Description
**DPS1**	For subjects who complete the study all data is included.<br><br>For subjects who discontinue IMP, post-discontinuation data will not be included. Includes data until discontinuation of treatment.
**DPS2**	For subjects who complete the study all data is included.<br><br>For subjects who discontinue IMP, all observed data will be included.

FAS and DPS1 are used to estimate the trial product estimand for the primary and key secondary objectives, whereas FAS and DPS2 are used to estimate the treatment policy estimand for the primary and key secondary objectives.

6 Statistical analyses

Statistical analyses will be carried out by ALK-Abelló A/S (hereafter ALK) Biometrics, Hørsholm, Denmark. All computation will be performed using the statistical analysis system SAS® version 9.4 or later.

The analyses described in this section along with the supporting information provided in section 9.2 specify the statistical analyses.

6.1 General considerations

In addition to the observed case analysis specified in the protocol, the primary endpoint and the key secondary endpoints are analysed using the estimand framework. For all other efficacy

endpoints, only the observed case analysis is performed. All efficacy endpoints are summarised by treatment and visit, and the primary and key secondary endpoints are listed.

All the statistical tests described in this section use a significance level of 5% and all tests and confidence intervals are two-sided. The null hypothesis is the hypothesis of no difference between treatment groups and the alternative to the null hypothesis is the hypothesis of difference. For each specific analysis the exact test is described in more detail.

Variation between countries/regions in the primary endpoint is expected and may result from variation in geography and thus HDM exposure, variation in standard treatment procedures, and possible differences in the conduct of the trial protocol. Variation between sites is also expected but is assumed to be small compared to the variation between countries and regions. The trial is not powered to detect differences or interactions in treatment effect within the country/region. Due to low number of subjects, France, Germany, and Spain will be pooled into the region Western Europe. The rest of the countries will not be pooled. Thus "country/region" will denote the effect of the individual countries as well as Western Europe.

6.1.1 Potential data issues

During study conduct, it was discovered that 11 subjects for various reasons had been randomised in error. Out of these, 10 subjects violated 1-2 inclusion criteria, and the last subject violated 1 exclusion criteria. As the inclusion criteria were systematically checked for all subjects in the trial, and because the violated inclusion criteria indicate that subjects potentially have a less severe HDM allergy, these subjects will be excluded from a sensitivitiy analysis.

However, as only one of these 10 subjects completed the trial, and the rest of the subjects discontinued prior to the start of the primary efficacy assessment period, only 1 of these subjects actually contributed to the observed case analyses. A list of the subject ID of the 10 subjects who violated at least one of the systematically checked inclusion criteria can be found in section 9.3.

The study includes several siblings. When siblings are included in the same cohort, there is a potential risk of mixing up their treatments at home, as well as mixing up/discussion of their diary recordings. Therefore siblings in the same cohort (56 in total) will be excluded from a sensitivity analysis. A list of the subject ID of the siblings can be found in section 9.3.

Several (8) subjects in Ukraine were temporarily without IMP due to the UKR crisis. These subjects will also be excluded from a sensitivity analysis. A list of the subject ID of the subjects in Ukraine without IMP can be found in section 9.3.

6.2 Primary endpoint analyses

6.2.1 Definition of endpoint

The primary endpoint is the average daily total combined rhinitis score (TCRS) of all observed daily values during the primary efficacy assessment period.

6.2.2 Primary analysis

The primary endpoint will be analysed using all subjects in FAS with at least 1 eDiary record during the primary efficacy assessment period. The presence of at least 1 eDiary record means that the primary endpoint can be calculated, so it is an observed case analysis. The analysis

will be performed using an LME. The model includes the square root of the average daily TCRS during the primary efficacy assessment period as response variable, treatment group and cohort as fixed factors, the square root of the baseline average daily TCRS as a covariate, country/region within cohort as a random effect, and with different residual errors specified for each treatment group. For further details see section 9.2.

Potential data issue: The primary observed case analysis will be repeated, but the subjects with potential data issues (randomised in error, siblings in same cohort, subjects in Ukraine without IMP as described in section 6.1.1) will be excluded from the analysis. The patients excluded from the analysis will be presented in a listing.

Sensitivity analysis: The primary analysis will be repeated using a modified DMS scoring to investigate the influence of subjects using more rescue medication than the recommended daily dose. The modified DMS scoring is defined in section 9.1.2.

Sensitivity analysis using bootstrapping: An additional sensitivitiy analysis will be performed using bootstrapping to calculate the 95% CI for the relative difference. For further details see section 9.2.

The primary analysis will also be repeated adding the cohort by treatment interaction, to investigate potential changes in treatment effect over time.

6.2.3 Applying the estimand framework

The study was not designed using the estimand framework, but since the first version of the protocol the ICH E9 addendum (ICH 2019, 1998) has come into affect. Therefore the primary analysis using an observed case analysis is supplemented with analyses of the trial product estimand and the treatment policy estimand using the estimand framework. However, as the study was not designed with these analyses in mind, data after IMP discontinuation was not collected for most subjects who discontinued the trial.

6.2.3.1 Trial product estimand

The primary endpoint will be compared between treatment groups using the **trial product estimand** for the FAS population and DPS1, following a hypothetical strategy.

6.2.3.1.1 Main analytical approach

Subjects for whom the primary endpoint is missing or unobserved (because of either missing diary data or the exclusion of diary data due to IMP discontinuation) will be included in the analysis through multiple imputation under the hypothetical situation where subjects continued to take study treatment as planned.

Multiple imputation for subjects missing the primary endpoint will be conducted defined as M1 in the following:

- For subjects who discontinue IMP due to lack of efficacy, multiple imputation of the missing endpoint will be from the placebo group. This assumes that had the subject continued to take study treatment, they would have experienced similar efficacy to subjects in the placebo group. Data is assumed to be MNAR.
- For subjects who do not discontinue IMP, and for subjects who discontinue IMP due to other reasons, multiple imputation of the missing endpoint will be from their own treatment group. This assumes that the level of efficacy that would have been

experienced by the subject if they had continued to take study treatment is unrelated to the reason for discontinuation and, if they had continued to take study treatment, they would have experienced similar efficacy to subjects in their own treatment group. Data is assumed to be MAR.

Analysis of the imputed datasets will be performed using the same model as the primary analysis using observed case analysis. The resulting treatment effect from the model is the main estimator. For more details see section 9.2.

6.2.3.1.2 Sensitivity analytical approach

Three sensitivity estimators will investigate/relax the assumptions about data missing at random.

Sensitivity estimator 1:

A sensitivity estimator for the trial product estimand, will relax the assumption about data missing at random for subjects discontinuing treatment due to IMP-related adverse events by imputing defined as M2 in the following:

- For subjects who discontinue IMP due to lack of efficacy or due to IMP-related adverse events, multiple imputation of the missing endpoint will be from the placebo group. This assumes that had the subject continued to take study treatment, they would have experienced similar efficacy to subjects in the placebo group. Data is assumed to be MNAR.
- For subjects who do not discontinue IMP, and for subjects who discontinue IMP due to other reasons, multiple imputation of the missing endpoint will be from their own treatment group. This assumes that the level of efficacy that would have been experienced by the subject if they had continued to take study treatment is unrelated to the reason for discontinuation and, if they had continued to take study treatment, they would have experienced similar efficacy to subjects in their own treatment group. Data is assumed to be MAR.

Sensitivity estimator 2:

Another sensitivity estimator will further relax the assumption about data missing at random by imputing defined as M3 in the following:

- For subjects who do not discontinue IMP, and for subjects who discontinue IMP due to lost to follow-up or withdrawal of consent, multiple imputation of the missing endpoint will be from their own treatment group. This assumes that the level of efficacy that would have been experienced by the subject if they had continued to take study treatment is unrelated to the reason for discontinuation and, if they had continued to take study treatment, they would have experienced similar efficacy to subjects in their own treatment group. Data is assumed to be MAR.
- For subjects who discontinue IMP due to other reasons, multiple imputation of the missing endpoint will be from the placebo group. This assumes that had the subject continued to take study treatment, they would have experienced similar efficacy to subjects in the placebo group. Data is assumed to be MNAR.

Sensitivity estimator 3:

In addition, in order to investigate the MAR assumption for the imputation in sensitivity estimator 2 above, a tipping point analysis will be performed as follows: A penalty (a number) is added to all imputed values in the active treatment group, and the analysis is repeated. The penalty is gradually increased until the point, the tipping point, where the null hypothesis is no longer rejected. If the tipping point is considered a clinical plausible difference, the tipping-point analysis does not support the sensitivity estimator.

6.2.3.2 **Treatment policy estimand**

In addition to the trial product estimand, the primary endpoint will be compared between treatment groups using the **treatment policy estimand** for the FAS population and DPS2, following a treatment policy strategy.

6.2.3.2.1 Main analytical approach

Subjects for whom the primary endpoint is missing (because of missing diary data) will be included in the analysis through multiple imputation as follows (M2).

- For subjects discontinuing IMP due to lack of efficacy or IMP-related adverse events, multiple imputation of the missing endpoint will be from the placebo group. Data is assumed to be MNAR.
- For subjects who do not discontinue IMP, and for subjects discontinuing IMP due to other reasons, multiple imputation of the missing endpoint will be from their own treatment group. This assumes that the missing data are MAR.

Analysis of the imputed datasets will be performed using the same model as the primary analysis using observed case analysis. The resulting treatment effect from the model is the main estimator. For more details see section 9.2.

Note that the above analysis is almost identical to the first sensitivity estimator for the trial product estimand, as data is collected after discontinuation of IMP for very few subjects only.

6.2.3.2.2 Sensitivity analytical approach

<u>Sensitivity estimator 1:</u> A sensitivity estimator will impute all missing values of the endpoint from the placebo group (M4).

6.3 Key secondary endpoint analyses

6.3.1 Definition of endpoints

The key secondary efficacy endpoints are the average of all observed daily values during the primary efficacy assessment period for rhinitis DSS, rhinitis DMS, and TCS.

6.3.2 Primary analysis

The key secondary endpoints “average rhinitis DSS”, “average rhinitis DMS”, and “average daily TCS”, will be analysed using an observed case analysis similar to the primary analysis of the primary endpoint. All subjects in the FAS with at least 1 eDiary record during the primary efficacy assessment period will be included. The analysis will be performed using an LME. The model includes the square root of the endpoint as response variable, treatment group and

cohort as fixed factors, the square root of the baseline endpoint as a covariate, country/region as a random effect, and with different residual errors specified for each treatment group.

Sensitivity analysis: For “average rhinitis DMS” and “average daily TCS” the primary analysis will be repeated using a modified DMS scoring to investigate the influence of subjects using more rescue medication than the recommended daily dose. The modified DMS scoring is defined in section 9.1.2.

6.3.3 Applying the estimand framework

In analogy with the supplemental analyses for the primary endpoint, the analysis of the key secondary endpoints will also be supplemented with analyses of the trial product estimand and the treatment policy estimand. The same main analytical approach as for the primary endpoint will be used for both estimands . No sensitivity estimators will be performed.

6.4 Summary of primary and key secondary endpoint analyses

Table 3 Overview of analyses for the primary and key secondary endpoints

Endpoint	Descriptions	Population	DPS	Model	Imputation Method	Analysis name
Average daily TCRS during the primary efficacy assessment period	Observed case	FAS	All	LME [b]	None	**Primary [a]**
						Sensitivity
						Potential data issue
	Trial product estimand		DPS1	LME with MI [b]	M1	Main analytical approach
					M2	Sensitivity 1
					M3	Sensitivity 2
					M3 tipping point	Tipping point
	Treatment policy estimand		DPS2	LME with MI [b]	M2	Main analytical approach
					M4	Sensitivity 1
Average rhinitis DSS during the primary efficacy assessment period	Observed case	FAS	All	LME [b]	None	**Primary [a]**
	Trial product estimand		DPS1	LME with MI [b]	M1	Main analytical approach
	Treatment policy estimand		DPS2	LME with MI [b]	M2	Main analytical approach

Average rhinitis DMS during the primary efficacy assessment period	Observed case	FAS	All	LME [b]	None	**Primary [a]**
						Sensitivity
	Trial product estimand		DPS1	LME with MI [b]	M1	Main analytical approach
	Treatment policy estimand		DPS2	LME with MI [b]	M2	Main analytical approach
Average daily TCS during the primary efficacy assessment period	Observed case	FAS	All	LME [b]	None	**Primary [a]**
						Sensitivity
	Trial product estimand		DPS1	LME with MI [b]	M1	Main analytical approach
	Treatment policy estimand		DPS2	LME with MI [b]	M2	Main analytical approach

[a] **Primary** refers to the analyses included in the test hierarchy

[b] Endpoints are square-root transformed before applying the model

6.5 Additional secondary efficacy endpoint analyses

The additional secondary efficacy endpoints, the average of all observed daily values during the primary efficacy assessment period for **rhinoconjunctivitis DSS**, for **rhinoconjunctivitis DMS**, for **rhinitis CSMS**, and for **rhinoconjunctivitis CSMS**, will be analysed using the same observed case analysis as specified for the primary endpoint. All subjects in the FAS with at least one eDiary record during the primary efficacy assessment period will be included. The analysis will be performed using an LME. The model includes the square root of the endpoint as response variable, treatment group and cohort as fixed factors, the square root of the baseline endpoint as a covariate, country/region as a random effect, and with different residual errors specified for each treatment group.

The **overall PRQLQ score at end of trial** will be analysed similar to the primary endpoint, but without the square root transformation, i.e. the LME will include the overall PRQLQ score as response variable, treatment group and cohort as fixed factors, the baseline overall PRQLQ score as a covariate, country/region as a random effect, and with different residual errors specified for each treatment group.

The overall **PRQLQ** score at V5 and V6 will be analysed separetely, similarly to the overall PRQLQ score at end of trial, and the model adjusted means for each treatment group will be plotted as a function of visit.

The endpoint **rhinitis mild days** during the primary efficacy assessment period consists of the daily binary responses “rhinitis mild day yes/no”, and will be analysed using a parametric GLMM with a logit link function. The model includes “rhinitis mild day yes/no” as the response variable, treatment group and cohort as fixed factors, the baseline average rhinitis TCRS as a covariate, and country/region within cohort, and subject as random effects. From the GLMM model, the

odds ratio for having a rhinitis mild day for active treatment relative to placebo will be presented together with the coherent p-values and 95% confidence limits.

Rhinitis exacerbation days will be analysed using the same model as for the analysis of rhinitis mild days, apart from that baseline average rhinitis DSS is included instead of baseline average rhinitis TCRS.

In the subgroup of subjects with asthma at baseline the following endpoints will be analysed:

- The average **asthma DSS** of all observed daily values during the primary efficacy assessment period, will be analysed using the same observed case analysis as specified for the key secondary endpoints.
- **SABA free days** will be analysed similar to "rhinitis mild days", but with baseline average asthma DSS included instead of baseline average rhinitis TCRS.
- The average of all observed values during the primary efficacy assessment period for **weekly number of puffs of as-needed SABA use**, will be analysed using the same observed case analysis as specified for the primary endpoint, however with the difference that both the endpoint and the baseline value will not be square root transformed.

6.5.1 Immunology

For Canada and Poland only, blood samples for immunological assessments were taken at screening and at end of trial. Data for the two IgE and IgG4 species, and total IgE, will be log10-transformed, whereas IgE-blocking factor will not be transformed. The assessments will be summarised by visit and treatment including change from baseline.

6.6 Explorative endpoint analyses

The explorative efficacy endpoints, the average of all observed daily values during the primary efficacy assessment period for average daily **TCCS**, for average **conjunctivitis DSS**, and for average **conjunctivitis DMS**, will be analysed using the same observed case analysis as specified for the primary endpoint.

The average of all observed daily values during the **2 weeks daily period collected 8 weeks** after treatment initiation for average daily **TCRS**, for average daily **TCS**, for average **rhinitis DSS**, and for average **rhinitis DMS**, will be analysed using the same observed case analysis as specified for the primary endpoint. All subjects in FAS with at least one eDiary record during the 2 weeks period collected 8 weeks after treatment initiation will be included.

The average of all observed daily values during the **2 weeks daily period collected 16 weeks** after treatment initiation for average daily **TCRS**, for average daily **TCS**, for average **rhinitis DSS**, and for average **rhinitis DMS**, will be analysed using the same observed case analysis as specified for the primary endpoint. All subjects in FAS with at least one eDiary record during the 2 weeks period collected 16 weeks after treatment initiation will be included.

Rhinitis symptom-free days will be analysed similar to "rhinitis mild days".

In the subgroup of subjects with asthma at baseline, **nocturnal awakenings due to asthma requirering SABA use**, will be analysed similar to "rhinitis mild days", but with baseline average asthma DSS included instead of baseline average rhinitis TCRS.

To investigate the effect of treatment as measured by average daily **TCRS during pollen season** in spring and summer, subjects will be classified according to whether or not they were

sensitised to pollen (tree/grass according to SPT) at baseline. Average daily TCRS during pollen season will be analysed for the **2 weeks daily period collected 8 weeks** after treatment initiation**,** for the **2 weeks daily period collected 16 weeks** after treatment initation, and for the **primary efficacy assessment period** separately. The analysis for each period will be similar to the observed case analysis specified for the primary endpoint, adding pollen sensitisation and pollen sensitisation by treatment interaction.

The model adjusted means for each treatment group will be plotted as a function of time for each group of subjects. For pollen sensitised subjects (tree/grass according to SPT), the primary efficacy assessment period is supposed to be outside the season of their pollen allergy. Therefore, the diary periods at 8 and 16 weeks can be seen as a reflection of spring and summer.

The endpoints “absence from school/work for a subject and parent/caregiver”, and “health care utilization”, will be summarised only.

6.7 Safety analyses

All safety summary tables and listings will be based on the SAF.

6.7.1 Extent of exposure

6.7.1.1 Treatment duration

The duration of treatment for each subject is calculated from the date of first dose up until (and including) the date of last IMP administration. For subjects who complete the trial this information is not available, instead the date of the last visit will be used.

Treatment duration will be summarised by treatment group.

6.7.1.2 Exposure

Exposure is a priori calculated as the difference between the number of tablets dispensed and the number of tablets returned or lost. But for a substantial number of subjects the information about tablets lost is missing, and is therefore assumed to be 0. To account for this, exposure will be calculated as the minimum of the difference between the number of tablets dispensed and the number of tablets returned or lost, and the treatment duration.

The number of tablets taken will be summarised by treatment group.

6.7.1.3 IMP compliance

Percentage compliance will be calculated for each subject as treatment taken divided by the treatment duration. The average percentage compliance will be presented by treatment group.

6.7.1.4 eDiary compliance for TCRS

eDiary compliance for TCRS is calculated for each subject as the number of daily records of TCRS in the period, divided by the expected number of days in the period. Note that daily TCRS is calculated based on rhinitis symptoms and rhinitis medication score, so a daily record of both is needed in order to have a daily record of TCRS.

The expected number of days in the period is 14 for baseline, TC1, and TC2, and 56 for the primary efficacy assessment period. The average percentage eDiary compliance will be presented by visit and treatment group.

6.7.2 Adverse events

6.7.2.1 Treatment emergent adverse events

An AE is considered a TEAE if the AE starts on or after the first IMP administration and no later than 7 days after the last IMP administration.

6.7.2.2 Solicited AEs

During the first 28 days after randomisation (V3), the subjects (together with a parent/caregiver) record in the eDiary whether they experience any of the 15 pre-specified symptoms/signs after IMP intake. These symptoms/signs (solicited term) and their corresponding PTs are listed in Table 4.

At Visit 4 the symptoms/signs reported in the eDiary are discussed with the subject. If they are assessed to be AEs, they are either reported as solicited AEs (by one of the solicited terms) or as regular AEs. Thus, there is not a one-to-one correspondence between the reporting of a symptom/sign in the eDiary and a solicited AE.

For solicited AEs, the PTs are assigned as shown in Table 4.

Table 4 Correspondance between solicited symptom/sign and PT

**Solicited symptom/sign**	**PT**
Food tastes different	Dysgeusia
Mouth ulcer	Mouth ulceration
Swelling in the back of the mouth	Mouth swelling
Itching in the mouth	Oral pruritus
Itching in the ear	Ear pruritus
Swelling of the lips	Lip swelling
Swelling of the tongue	Swollen tongue
Tongue pain	Glossodynia
Tongue ulcer	Tongue ulceration
Throat irritation/tickle	Throat irritation
Throat swelling	Pharyngeal swelling
Stomach pain	Abdominal pain upper
Nausea (feel like throwing up)	Nausea

Vomiting	Vomiting
Diarrhoea	Diarrhoea

6.7.2.3 Events of special interest

Events of special interest for this trial are:

- Anaphylactic reactions, anaphylaxis and/or systemic allergic reactions
- Events treated with adrenaline/epinephrine
- Severe local swelling of the mouth and/or throat
- Eosinophilic oesophagitis

6.7.2.4 Reporting of AEs

All TEAEs will be summarised by treatment group according to causality, severity (mild, moderate, severe), seriousness, action taken, outcome, and whether the event led to IMP discontinuation. The number of subjects in treatment group, the frequency and proportion of subjects having the event, as well as the number of events and proportion of events will be displayed. A similar summary table will be produced for all IMP-related TEAEs.

All TEAEs and all IMP-related TEAEs will be summarised (in separate tables) by treatment group, system organ class, and preferred term, displaying number of subjects in treatment group, and number and frequency of subjects having the event. The tables will be sorted according to most frequent SOC and PT in the active treatment group, and the most common TEAEs (preferred term from MedDRA version 23.0) defined as those that are present in at least 2% of subjects in the active treatment group will be marked.

In addition IMP-related solicited TEAEs will be summarised by treatment group, and presented by PT.

Severe TEAEs will be listed only, if there are fewer than 10 events in total. Otherwise severe TEAEs will also be summarised and presentented by SOC and PT similar to how TEAEs are presented. The same goes for SAEs, TEAEs leading to IMP discontinuation, and IMP-related TEAEs leading to IMP disruption.

For most frequent IMP-related TEAEs (present in at least 2% of subject in the active treatment group) onset and duration will be presented as follows: Average time to onset and average duration both in days, will be presented by PT and treatment. In addition, for day 1 of treatment average time to onset in minutes will be presented by PT and treatment.

For the most frequent IMP-related TEAEs which are recurrent, the average daily duration and the average duration in days from first to last occurrence will be presented by PT and treatment.

A table with the overall summary of the safety profile will be produced showing subjects with TEAEs, IMP-related TEAEs, IMP-related severe TEAEs, IMP-related treatment-emergent SAEs, IMP-related TEAEs leading to IMP discontinuation, IMP-related solicited TEAEs, and each type of IMP-related treatment-emergent ESI by treatment group.

Serious TEAEs, severe TEAEs, TEAEs leading to IMP discontinuation, IMP-related TEAEs leading to IMP interruption, Individual ESIs, TEAEs, and all non-treatment-emergent AEs for the total analysis set will be listed. Medication errors will also be listed.

6.7.3 Additional safety assessments

Shift tables will be produced for physical examination and oropharyngeal examination including the categories normal, abnormal not clinically significant, abnormal clinically significant.

Shift tables will be produced for laboratory assessments including the categories low, normal, high, and missing as relevant, with the exception is urinalysis, where the shift table will include the categories normal, abnormal not clinically significant, and abnormal clinically significant.

Vital signs and the pulmonary function test parameters (FVC, FVC percent predicted, FEV_1, and FEV_1 percent predicted) will be summarised by visit and treatment group.

6.8 Subgroup analyses

No formal statistical subgroup analyses were planned in the protocol. But as the trial includes subjects with HDM allergic rhinitis/rhinoconjunctivitis with or without asthma, the subgroup of subjects with asthma at baseline will be examined. The observed case analysis for the primary and key secondary endpoints will be repeated adding asthma subgroup and asthma subgroup by treatment interaction.

6.9 Interim analysis

No interim analyses will be performed.

6.10 Changes and/or deviations to protocol-planned analyses

The sensitivitiy analyses of the primary endpoint are not performed as specified in the protocol, and neither is the specified MMRM analysis. Instead the planned observed case analyses are supplemented with analyses using the estimand framework and several corresponding sensitivity analyses. A few other sensitivity analyses are also added for the primary endpoint. For the primary endpoint and the key secondary endpoints using medication score, a sensitivity analysis is added to investigate the influence of subjects using more medication than the recommended daily dose.

The “overall PRQLQ score at end of trial” was included as the 5th and last test in the testing hierarcy.

Additional secondary and explorative endpoints are added:

- “Absence from school/work for a subject and parent/caregiver”
- “Health care utilisation”

Some endpoints are redefined to make them more clear:

- The endpoint “average daily use of SABA” is changed into two endpoints: “SABA free days” and “average weekly number of puffs of as-needed SABA use”
- The “endpoint CSMS” is specified as “average daily rhinitis CSMS” and “average daily rhinoconjunctivitis CSMS”

The distribution of IMP discontinuations will be visualised by a band plot, instead of by a Kaplan-meier plot and a cumulative incidence plot, as the band plot also shows the different reasons for discontinuation. The band plot is also used to visualize time to discontinuation due to TEAEs.

Subgroup analyses of the primary and the key secondary endpoints according to asthma status have been added.

7 Sample size determination

The primary efficacy endpoint is the “average daily TCRS during the 8-week primary efficacy assessment period”.

7.1 Assumptions

The primary efficacy analysis is based on treatment comparison performed using a two-sided t-test on a 5% level of significance in a linear mixed effect (LME) model assuming unequal variances. The TCRS is square root transformed in the LME model in order to obtain a better approximation to a normal distribution. Least squares means are then back-transformed to the original scale by taking the square. In addition, the relative difference of the back-transformed least squares means will be calculated together with 95% confidence limits. The latter will be calculated based on Fieller’s theorem.

Superiority of active treatment compared to placebo is confirmed when the p-value for the primary efficacy analysis is <0.05 in the t-test. An additional FDA acceptance criteria for the trial is a point estimate of the relative difference to placebo of no more than -15% with an associated upper bound of the 95% CI being at most -10%.

Data from previous adult phase III trials of the HDM tablet conducted in Europe (MT-06) and North America (P001) and data from a previous paediatric trial (5-17 years of age) of the HDM tablet conducted in Japan (TO-203-3-3) is used for power calculation. Table 5 shows the mean and standard deviation of the square root transformed TCRS by treatment group for the MT-06 trial. The power calculation is based on the mean for placebo and the standard deviation for the placebo and the 12 SQ-HDM treatment group observed in the MT-06 trial due to the similar nature of the trial design for the MT-12 and MT-06 trial. Table 6 shows the estimated effect sizes in MT-06, P001 and TO-203-3-3 for different age groups. Power calculation is performed based on the assumption that the effect size is similar to the effect size seen in the paediatric trial TO-203-3-3 for the age group 5-11 years (~21%). In addition, power calculation is based on the more conservative assumption that the effect size is similar to the effect sizes observed in the previous adult trials MT-06 and P001 conducted in Europe and North America (~17-18%).

Table 5 Mean and standard deviation of TCRS by treatment group in MT-06

Square root transformed TCRS	MT-06	
	Placebo	12 SQ-HDM
Raw mean	2.65	2.40
Standard deviation	0.89	0.95

Table 6 Effect size of TCRS in MT-06, P001, and TO-203-3-3

Trial<br>Region	MT-06<br>Europe	P001<br>North America		TO-203-3-3<br>Japan		
Age group	18-65	18-65	12-17[A]	5-17	5-11[A]	12-17[A]
N	582	1186	160	427	247	180
Effect size	-18.1%[B]	-17.2%[C]	-22.4%[C]	-22.5%[B]	-20.8%[B]	-25.9%[B]

[A] Subgroup analysis; [B] Relative difference (Active-Placebo)·100%/Placebo in adjusted means; [C] Relative difference (Active-Placebo)·100%/Placebo in medians

7.2 Results of sample size calculation

Table 7 shows the results for the power calculation. Power calculation for fulfilling the acceptance criteria of a p-value <0.05 is based on Satterthwaite unpooled two-sided t-test on a 5% level of significance. Power calculation for fulfilling the acceptance criteria of an upper bound of the confidence limit being at most -10% (95% UCL ≤ -10%) is conducted by simulations using Fieller's theorem (Fieller 1954).

Table 7 Power calculations based on different assumptions of the effect size using observed case approach

Effect size	-18%		-21%	
Acceptance criterion	p-value <0.05	95%UCL ≤-10	p-value <0.05	95%UCL ≤-10
Sample size per arm	Power			
300 subjects	91%	35%	97%	58%
500 subjects	99%	51%	100%	80%
580 subjects	100%	58%	100%	85%

95% UCL: upper bound of 95% confidence interval.

Based on Table 7 a sample size of 580 per treatment arm is chosen. With an effective sample size of 580 subjects per treatment arm, the MT-12 trial have:

- 85% power (2-sided, α=0.05) to obtain an upper bound of the 95% CI for a relative difference of no more than -10% if assuming an effect size of -21%
- >90% power (2-sided, α=0.05) to detect a statistically significant difference (p-value<0.05) if assuming an effect size of -18%.

When further adjusting for a drop-out that may be approximately 15% based on experience from MT-06 and P001, the proposed sample size per treatment arm for the MT-12 trial is 682 subjects (Ntotal=1364 ~ 1370 subjects).

8 Demographics and baseline characteristics

Summaries for numerical variables will display the descriptive statistics mean, SD, median, minimum and maximum.

Summaries for categorical variables will be frequency tables displaying numbers and percentages.

8.1 Screening failures

Reasons for screening failure will be summarised for the total analysis set. Reasons for screening failure will also be listed.

8.2 Protocol deviations

Important PDs and important subject-level PDs will be summarised. In addition important trial-level, country-level, site-level, and subject-level protocol deviations will be listed.

8.3 Subject disposition

Subject disposition will be summarised by number and percentage of subjects screened, randomised, included in the analysis sets, discontinued and the primary reason for discontinuation by treatment group will be presented.

IMP discontinuations over time by different reasons will be visualised by a band plot showing proportions on IMP, having discontinued IMP due to AE, withdrawal by subject, and all other reasons (pooled). IMP and trial discontinuations will be listed.

8.4 Baseline characteristics

Demographic variables (age, sex, race, ethnic origin, and country/region) will be summarised by treatment group. Demographic data will also be listed.

Baseline characteristics (mono/poly sensitised, duration of HDM AR/C, skin prick test results, skin pric test wheal sizes (for HDM), specific HDM IgE (both continuous and categorised), asthma status and severity, and use of rescue medication for rhinitis at baseline) will be summarised by treatment group.

8.5 Medical history

Medical history (including history of rhinitis, conjunctivitis, asthma, atopic dermatitis and food allergy) will be summarised by treatment group. Separate tables will be created for allergy, asthma, and atopic dermatitis medical history, and other medical history.

8.6 Prior and concomitant therapy

Prior and concomitant medication will be summarised separately by treatment group.

9 Supporting documentation

9.1 Derivations for the study

9.1.1 Derivation of daily symptom and medication scores

Symptoms and medication use are recorded daily by the subject in the eDiary. For each day it is only possible for the subject to either record all or nothing at all.

9.1.1.1 **Rhinitis DSS**

The rhinitis DSS consists of the 4 rhinitis symptoms: runny nose, blocked nose, sneezing, and itchy nose. Each of the symptoms is scored from 0 to 3 by the subject daily. No symptoms=0, mild symptoms=1, moderate symptoms=2, and severe symptoms=3. The rhinitis DSS is the sum of the 4 individual rhinitis symptom scores and ranges from 0 to 12.

9.1.1.2 **Conjunctivitis DSS**

The conjunctivitis DSS consists of the 2 conjunctivitis symptoms: gritty feeling/red/itchy eyes, and watery eyes. Each symptom is scored from 0 to 3 by the subject daily. No symptoms=0, mild symptoms=1, moderate symptoms=2, and severe symptoms=3. The conjunctivitis DSS is the sum of the 2 individual conjunctivitis symptom scores and ranges from 0 to 6.

9.1.1.3 **Rhinoconjunctivitis DSS**

The rhinoconjunctivitis DSS is a sum of rhinitis DSS and conjunctivitis DSS, and ranges from 0 to 18.

9.1.1.4 **Rhinitis DMS**

The rhinitis DMS is the sum of the total daily scores for each type of rescue medication (Table 8).

Note that the recommended dose of Desloratadine is scored 4, and that doses below are scored proportionally, i.e. if a 12 years old takes 5 ml once daily, this is scored as 2. Similarly lower than recommended use of Loratadine tablets and Mometasone furoate nasal spray is also scored proportionally based on the recommended daily dose. Some subjects took Loratadine tablets, although they should have taken Desloratadine oral solution. Their dosing is scored the same as if they should have taken tablets, i.e. a score of 4 for 1 tablet, and a score of 2 for half a tablet. The rhinitis DMS ranges from 0 to 12.

Table 8 Rhinitis DMS scoring

Rescue medication	Subject dosing	Score/Dose unit	Maximum daily score
Desloratadine oral solution, 0.5 mg/ml	5 years old:<br>2.5 ml (1.25 mg) once daily	4	4
	6-11 years old:<br>5 ml (2.5 mg) once daily		

	≥12 years old:<br>10 ml (5 mg) once daily		
Loratadine tablets, 10 mg	6-12 years old and > 30 kg:<br>1 tablet (10 mg) once daily		
	>12 years old: 1 tablet (10 mg) once daily		
Mometasone furoate nasal spray, 50 micrograms/dose	<12 years: 1 puffs in each nostril once daily	4	8
	≥12 years: 2 puffs in each nostril once daily	2	
Max. daily rhinitis medication score			12

For Desloratadine and Loratadine, the dose unit corresponds to the recommended dose. For Mometasone, the dose unit is 1 puff in 1 nostril.

9.1.1.5 Conjunctivitis DMS

The conjunctivitis DMS is the sum of the total daily scores for each type of rescue medication (Table 9). Note that the recommended dose of desloratadine is scored 2, and that doses below are scored proportionally, i.e. if a 12 years old takes 5 ml once daily, this is scored as 1. Similarly lower than recommended use of Loratadine tablets and Olopatadine eye drops is also scored proportionally based on the recommended daily dose. Some subjects took Loratadine tablets, although they should have taken Desloratadine oral solution. Their dosing is scored the same as if they should have taken tablets, i.e. a score of 2 for 1 tablet, and a score of 1 for half a tablet.

The conjunctivitis DMS ranges from 0 to 8.

Table 9 Conjunctivitis DMS Scoring

Rescue medication	Subject dosing	Score/Dose unit	Maximum daily score
Desloratadine oral solution, 0.5 mg/ml	5 years old:<br>2.5 ml (1.25 mg) once daily	2	2
	6-11 years old:<br>5 ml (2.5 mg) once daily		
	≥12 years old:<br>10 ml (5 mg) once daily		
Loratadine tablets, 10 mg	6-12 years old and > 30 kg:<br>1 tablet (10 mg) once daily		
	>12 years old: 1 tablet (10 mg) once daily		
Olopatadine eye drops, 1 mg/ml	1 drop in each eye twice daily	1.5	6

Max. daily conjunctivitis medication score	8

For Desloratadine and Loratadine, the dose unit corresponds to the recommended dose. For Olopatadine, the dose unit is 1 drop in 1 eye.

9.1.1.6 **Rhinoconjunctivitis DMS**

The rhinoconjunctivitis DMS is the sum of the rhinitis DMS and the conjunctivitis DMS, and ranges from 0 to 20.

9.1.1.7 **TCRS**

The TCRS is the sum of the rhinitis DSS and the rhinitis DMS, and ranges from 0 to 24.

9.1.1.8 **TCCS**

The TCCS is the sum of the conjunctivitis DSS and the conjunctivitis DMS, and ranges from 0 to 14.

9.1.1.9 **TCS**

The TCS is the sum of the rhinoconjunctivitis DSS and the rhinoconjunctivitis DMS, and ranges from 0 to 38.

9.1.1.10 **Asthma DSS**

The 4 symptoms of asthma: cough, wheezing, chest tightness, and shortness of breath, are scored from 0 to 3 by the subject daily. No symptoms =0, mild symptoms =1, moderate symptoms =2, and severe symptoms =3. The asthma DSS is the sum of the 4 individual asthma symptom scores, and ranges from 0 to 12.

9.1.1.11 **Rhinitis CSMS**

The rhinitis CSMS recommended by the EAACI consists of symptom and medication scoring.

The 4 EAACI rhinitis symptoms: runny nose, blocked nose, sneezing, and itchy nose are scored. Each symptom is scored from 0 to 3 by the subject daily. No symptoms =0, mild symptoms =1, moderate symptoms=2, and severe symptoms 3. The rhinitis CSMS symptom score is constructed as the sum of the individual symptoms score divided by the number of symptoms (4) and thus range from 0 to 3.

The rhinitis CSMS medication score is a stepwise scoring method, which was not implemented in this trial. However, the rhinitis CSMS medication scoring is still implemented where the highest medication step score is used as the medication score. The summary of the steps is presented in Table 10.

Table 10 Rhinitis Medication Scoring for CSMS by EAACI task force

Step	Medication	Rhinitis Medication Score

1	Desloratadine oral solution or Loratadine tablets	1
2	Mometasone furoate nasal spray	2

If the medication is not used, then the medication score is 0. The range of medication score is from 0 to 2. Note that in Pfaar et al (Pfaar et al. 2014) a step 3 of oral corticosteroids was also included, but in this trial oral corticosteroids were not provided as rescue medication, hence the step 3 is not possible.

The rhinitis CSMS by EAACI is the sum of rhinitis symptoms and the rhinitis medication scores, and ranges from 0 to 5.

9.1.1.12 **Rhinoconjunctivitis CSMS**

The rhinoconjunctivitis CSMS recommended by the EAACI consists of symptom and medication scoring.

The 6 EAACI rhinoconjunctivitis symptoms are scored: runny nose, blocked nose, sneezing, itchy nose, gritty feeling/red/itchy eyes, and watery eyes. Each symptom is scored from 0 to 3 by the subject daily. No symptoms=0, mild symptoms=1, moderate symptoms=2, and severe symptoms=3. The rhinoconjunctivitis symptoms score is constructed as the sum of the individual symptoms score divided by the number of symptoms (6) and thus range from 0 to 3.

The rhinoconjunctivitis CSMS medication score is a stepwise scoring method, which was not implemented in this trial. However, the rhinitis CSMS medication scoring is still implemented where the highest medication step score is used as a medication score. The summary of the steps is presented in Table 11.

Table 11 Rhinoconjunctivitis medication scoring for CSMS by EAACI task force

Step	Medication	Rhinitis Medication Score
1	Desloratadine oral solution or Loratadine tablets or/and Olopatadine eye drops	1
2	Mometasone furoate nasal spray	2

If the medication is not used, then the medication score is 0. The range of medication score is from 0 to 2. Note that in Pfaar et al (Pfaar et al. 2014) a step 3 of oral corticosteroids was also included, but in this trial oral corticosteroids were not provided as rescue medication, hence the step 3 is not possible.

The rhinoconjunctivitis CSMS by EAACI is the sum of rhinoconjunctivitis symptoms and the rhinoconjunctivitis medication scores, and ranges from 0 to 5.

9.1.2 Medication scores used for sensitivity analyses

DMS_sens scores will be used for sensitivity analyses for the primary endpoint average daily TCRS, and the key secondary endpoints average rhinitis DMS and average daily TCS, all during the primary efficacy assessment period.

9.1.2.1 **Rhinitis DMS_sens**

The rhinitis DMS_sens is the sum of the total daily scores for each type of rescue medication (Table 12).

Note that the recommended dose of Desloratadine is scored 4, and that doses above or below are scored proportionally, i.e. if a 12 years old takes 5 ml once daily, this is scored as 2. And if the same subject takes 20 ml once daily, this is scored as 8.

Similarly doses of Loratadine tablets and Mometasone furoate nasal spray are also scored proportionally based on the recommended daily dose. Some subjects took Loratadine tablets, although they should have taken Desloratadine oral solution. Their dosing is scored the same as if they should have taken tablets, i.e. a score of 4 for 1 tablet, and a score of 2 for half a tablet. If a subject took 2 tablets (exceeded the recommended dose) this is scored as 8.

The rhinitis DMS_sens ranges from 0 to 12, unless the recommend dose is exceeded, in which case the max of 12 is also exceeded.

Table 12 Rhinitis DMS_sens scoring

Rescue medication	Subject dosing	Score/Dose unit	Score for recommended dose
Desloratadine oral solution, 0.5 mg/ml	5 years old:<br>2.5 ml (1.25 mg) once daily	4	4
	6-11 years old:<br>5 ml (2.5 mg) once daily		
	≥12 years old:<br>10 ml (5 mg) once daily		
Loratadine tablets, 10 mg	6-12 years old and > 30 kg:<br>1 tablet (10 mg) once daily		
	>12 years old: 1 tablet (10 mg) once daily		
Mometasone furoate nasal spray, 50 micrograms/dose	<12 years: 1 puff in each nostril once daily	4	8
	≥12 years: 2 puffs in each nostril once daily	2	
Max. daily rhinitis medication score for recommended dose			12

For Desloratadine and Loratadine, the dose unit corresponds to the recommended dose. For Mometasone, the dose unit is 1 puff in 1 nostril.

9.1.2.2 **Conjunctivitis DMS_sens**

The conjunctivitis DMS_sens is the sum of the total daily scores for each type of rescue medication (Table 13).

Note that the recommended dose of desloratadine is scored 2, and that doses above or below are scored proportionally, i.e. if a 12 years old takes 5 ml once daily, this is scored as 1. Similarly use of Loratadine tablets and Olopatadine eye drops is also scored proportionally based on the recommended daily dose. Some subjects took Loratadine tablets, although they should have taken Desloratadine oral solution. Their dosing is scored the same as if they should have taken tablets, i.e. a score of 2 for 1 tablet, and a score of 1 for half a tablet. If a subject took 2 tablets (exceeded the recommended dose) this is scored as 8.

The conjunctivitis DMS_sens ranges from 0 to 8, unless the recommend dose is exceeded, in which case the max of 8 also exceeded.

Table 13 Conjunctivitis DMS_sens scoring

Rescue medication	Subject dosing	Score/Dose unit	Score for recommended dose
Desloratadine oral solution, 0.5 mg/ml	5 years old:<br>2.5 ml (1.25 mg) once daily	2	2
	6-11 years old:<br>5 ml (2.5 mg) once daily		
	≥12 years old:<br>10 ml (5 mg) once daily		
Loratadine tablets, 10 mg	6-12 years old and > 30 kg:<br>1 tablet (10 mg) once daily		
	>12 years old: 1 tablet (10 mg) once daily		
Olopatadine eye drops, 1 mg/ml	1 drop in each eye twice daily	1.5	6
Max. daily conjunctivitis medication score for recommended dose			8

For Desloratadine and Loratadine, the dose unit corresponds to the recommended dose. For Olopatadine, the dose unit is 1 drop in 1 eye.

9.1.2.3 **TCRS_sens**

The TCRS_sens is the sum of the rhinitis DSS and the rhinitis DMS_sens, and ranges from 0 to 24, unless the recommended daily dose is exceeded.

9.1.2.4 **TCS_sens**

The TCS_sens is the sum of the rhinoconjunctivitis DSS and the rhinoconjunctivitis DMS_sens (sum of rhinitis DMS_sens and conjunctivitis DMS_sens), and ranges from 0 to 38, unless the recommended daily dose is exceeded.

9.1.3 Derivation of average symptom and medication scores

For each observation period the average score for that period is calculated as the mean of all observed (i.e., non-missing) daily values in that period.

The observation periods considered are:

- Baseline period: last 2 weeks of the 3 weeks recorded after V2 (baseline visit)
- Maintenance period 1: starts at TC1 (8 weeks after IMP initiation) and lasts for 2 weeks
- Maintenance period 2: starts at TC2 (16 weeks after IMP initation) and lasts for 2 weeks
- Primary efficacy period: starts at V6 (44-49 weeks after IMPinitation) and lasts for 8 weeks

The averages are calculated for the following scores:

- TCRS
- Rhinitis DSS
- Rhinitis DMS
- TCS
- Rhinoconjuctivitis DSS
- Rhinoconjuctivitis DMS
- Asthma DSS
- Rhinitis CSMS
- Rhinoconjunctivitis CSMS
- TCSS
- Conjunctivitis DSS
- Conjunctivitis DMS
- TCRS_sens
- Rhinitis DMS_sens
- TCS_sens

9.1.4 Derivation of daily binary variable

9.1.4.1 Rhinitis mild day

Rhinitis mild day is a day with no symptoms or mild symptoms (i.e. rhinitis DSS= 0 or rhinitis DSS=1) and no use of symptoms medication (i.e. rhinitis DMS = 0). Rhinitis mild day is a binary endpoint where 1 is a rhinitis mild day, and 0 otherwise.

9.1.4.2 Rhinitis exacerbation day

A rhinitis exacerbation days is a day with a rhinitis DSS of at least 6 or a day with a rhinitis DSS of 5 and 1 individual symptom scored 3. Rhinitis exacerbation day is a binary endpoint where 1 is a day with rhinitis exacerbation, and 0 otherwise.

9.1.4.3 SABA free day

A SABA free days is a day where the subject did not use SABA. A SABA free day is a binary endpoint where 1 is a day where the subject did not use SABA, and 0 otherwise.

9.1.4.4 Rhinitis symptom-free day

Rhinitis symptom-free day is a day with no symptoms and no use of symptomatic medication (i.e. rhinitis DSS=0 and rhinitis DMS= 0). Rhinitis symptom-free day is a binary endpoint where 1 is a symptom-free day, and 0 otherwise.

9.1.4.5 Nocturnal awakenings due to asthma requiring SABA use

Nocturnal awakening due to asthma requiring SABA use is a binary endpoint where 1 is a day where the subject woke up due to asthma requiring SABA use, and 0 otherwise.

9.1.5 Other derivations of endpoints

9.1.5.1 Average weekly number of puffs of as-needed SABA use

For each observation period the average daily number of puffs of as-needed SABA use for that period is calculated as the mean of all observed (i.e., non-missing) daily values in that period.

The observation periods considered are:

- Baseline period: starts at V2 (baseline visit) and lasts for 2 weeks
- Primary efficacy period: starts at V6 (44-49 weeks of treatment) and lasts for 8 weeks

Then the average weekly number of puffs of as-needed SABA use is calculated as 7 times the average of the daily number of puffs of as-needed SABA use.

9.1.5.2 PRQLQ

The PRQLQ has 23 questions in 5 domains (nasal symptoms, eye symptoms, practical problems, activity limitation, and other symptoms (Juniper et al. 1998).

The PRQLQ is completed by an interviewer. Subjects are asked to think about how they have been during the previous week and to respond to each of the 23 questions on a 7-point scale (from 0 = not bothered/none of the time to 6 = extremely bothered/all of the time). The overall PRQLQ score is the mean of all 23 responses and the individual domain scores are the means of the items in those domains.

9.1.5.3 Absence from school/work for a subject and parent/caregiver

The “Absence from school/work for a subject and parent/caregiver” questionnaire consists of 4 questions:

1. Was subject absent from school/work due to subject's allergy and/or asthma?
2. If yes, how many days?
3. Was parent/caregiver absent from work due to subject's allergy and/or asthma?
4. If yes, how many days?

Questions number 1 and 3 have binary outcomes (Yes=1/No=0), and the questions number 2 and 3 are integer outcomes.

9.1.5.4 Health care utilisation

The health care utilisation questionnaire consists of 2questions:

1. Was any health care utilisation required?
2. Number of visits to hospitals, emergency room and doctor (specialist) due to subject's allergy and/or asthma?

Question number 1 has a binary outcome (Yes=1/No=0) and question number 2 has an integer outcome.

9.1.6 Imputation of dates

9.1.6.1 Partial dates in AE reporting

Partial dates and time for **start** of AEs are imputed as follows:

- Missing day is imputed as maximum of first day of month and day of first IMP
- Missing month is imputed as maximum of first day in January and date of first IMP

Partial dates and time for **end** of AEs are imputed as follows:

- Missing day is imputed as minimum of last day in month and day of last contact
- Missing month is imputed as minimum last day of December and date of last contact

With these imputation rules AEs are considered treatment-emergent when dates are partial and the existing information does not exclude the possibility of the event being treatment-emergent.

9.1.6.2 Incomplete date for last IMP

Any partial date of last dose is imputed as follows:

- Missing day is imputed as minimum of last day in month and day of last visit.
- Missing month is imputed as minimum of last day of December and date of last visit.

9.1.6.3 Imputation for date of birth

When only month and year of birth is known, the date is imputed as the last day of the month. When only year is known, the date is imputed as the last day of the year.

9.1.7 Other derivations

9.1.7.1 Subjects with reported asthma

Subjects with reported asthma are defined as the subjects with allergic asthma at baseline according to the eCRF, along with subjects with asthma medical history as registered by MHLLTCD=10003553 or MHLLTCD=10003555.

9.1.7.2 eDiary periods

For some subjects the eDiary period has by mistake been longer than planned. In these cases it is decided to define the period to be the last part of the eDiary period. Thus for baseline the last 14 days up until one day before first dose will be used, for TC1 and TC2 the last 14 days will be used, and for the primary efficacy assessment period, the last 8 weeks of the eDiary period will be used.

9.1.7.3 Age for DMS

For each subject and each eDiary period, age is calculated at the first day of the eDiary period, and this age is used throughout the period.

9.2 Further details pertaining to statistical analyses

9.2.1 LME analyses

Adjusted means for each treatment group, the absolute treatment difference (Placebo – Active) with 95% confidence interval and p-value, and the relative treatment difference (Placebo – Active/ Placebo) with 95% confidence interval will be presented.

Each endpoint is calculated as the simple average of all the observed daily values during the relevant period as defined in section 9.1.7.2.

9.2.1.1 Without multiple imputation

When the endpoint is not transformed

The endpoint is analysed as the response variable in an LME which includes treatment group and cohort as fixed factors, the baseline value as a covariate, country/region as a random effect, and with different residual errors specified for each treatment group. Denominator degrees of freedom will be calculated using the Kenward and Roger’s approximation (Kenward and Roger 1997). The p-value for the absolute difference is reported as the test result. The 95% confidence interval for the relative difference will be calculated using Fieller’s theorem (Fieller 1954).

When the endpoint is square root transformed

The endpoint is square root transformed and analysed as the response variable in an LME which includes treatment group and cohort as fixed factors, the square root of the baseline value as a covariate, country/region within cohort as a random effect, and with different residual errors specified for each treatment group. Denominator degrees of freedom will be calculated using the Kenward and Roger’s approximation (Kenward and Roger 1997). The p-value for the absolute difference is reported as the test result.

The results are back-transformed as follows; from the LME, estimated least square means on the square root transformed scale are output along with associated covariance matrix. For the absolute difference the SE is approximated by using the first order Delta method (first order Taylor approximation), and from this the 95% CI is calculated. For the relative difference, Fieller's theorem (Fieller 1954) is used to calculate the 95% CI for X/Y, and then the 95% confidence bounds for $(Y^2-X^2)/Y^2 = 1 - (X/Y)^2$ are found by applying the monotone transformation $f(r)= 1 - r^2$ to the confidence interval.

Sensitivity analysis using bootstrapping

For the primary endpoint, a sensitivity analysis will be performed where the 95% CI for the relative difference on the original scale, instead of using Fieller's theorem and a monotone bacl-transformation, will be calculated using bootstrapping. The bootstrapping which will be used is the "non-parametric residual bootstrap" by Carpenter et al (Carpenter et al. 2003).

Model checking:

The assumption of normally distributed residuals underlying the LME will be evaluated by visual inspection of quantile-quantile plots for the primary and key secondary endpoints. If the square root transformation does not result in a good approximation to the normal distribution, no transformation will be applied if this improves the approximation to a normal distribution

Convergence issues:

If the model does not converge, country/region will be pooled into larger regions. If the model still does not converge, the random effect of region within cohort will be removed from the model.

9.2.1.2 With multiple imputation

Imputation will be done using the method of unrestricted random sampling with replacement (seed=686) and 1000 multiple imputed datasets will be created.

When the endpoint is not transformed

Each of the 1000 multiple imputed datasets is analysed using the same model as without imputation. The endpoint is analysed as the response variable in an LME which includes treatment group and cohort as fixed factors, the baseline endpoint as a covariate, country/region as a random effect, with different residual errors specified for each treatment group, and with denominator degrees of freedom calculated using the Kenward and Roger's approximation (Kenward and Roger 1997).

Rubin's rule is used to combine the 1000 analysis results across the multiple imputed datasets. The p-value for the absolute difference is reported as the test result. The 95% confidence interval for the relative difference is calculated using Fieller's theorem (Fieller 1954).

When the endpoint is square root transformed

The endpoint is square root transformed. Each of the 1000 multiple imputed datasets is analysed using the same model as without imputation. The square root transformed endpoint is analysed as the response variable in an LME which includes treatment group and cohort as fixed factors, the square root of the baseline endpoint as a covariate, country/region as a random effect, with different residual errors specified for each treatment group, and with

denominator degrees of freedom calculated using Kenward and Roger's approximation (Kenward and Roger 1997).

Rubin's rule is used to combine the 1000 analysis results across the multiple imputed datasets. The p-value for the absolute difference is reported as the test result.

The results are back-transformed as follows; from the LME, estimated least square means on the square root transformed scale are output along with associated covariance matrix. For the absolute difference the SE is approximated by using the first order Delta method (first order Taylor approximation), and from this the 95% CI is calculated. For the relative difference, Fieller's theorem (Fieller 1954) is used to calculate the 95% CI for X/Y, and then the 95% confidence bounds for $(Y^2-X^2)/Y^2 = 1 - (X/Y)^2$ are found by applying the monotone transformation $f(r)= 1 - r^2$ to the confidence interval.

9.2.1.3 Square root transformation

The LME relies on the assumption of normally distributed residuals. Therefore the square root transformation is generally applied, as this usually results in a good approximation to the normal distribution. In addition, a lot of subjects do not use rescue meddication, so the average DMS score is often 0, and this is also accounted for by using the square root transformation, and a reason the logarithm transformation is not applied.

9.2.1.4 Mean and SE

Let μ_{Active}, μ_{Active}, $\sigma_{Placebo}$, and σ_{Active}be the resulting means and variances for placebo and active treatment and cov be the covariance. Then the back-transformed means are given by:

- $\hat{\mu}_{Placebo} = (\mu_{Placebo})^2$
- $\hat{\mu}_{Active} = (\mu_{Active})^2$
- $\hat{\mu}_{Difference} = (\mu_{Placebo})^2 - (\mu_{Active})^2$

and the back-transformed standard errors are given by

- $SE_{Active} = \sqrt{(2\mu_{Active})^2\sigma_{Active}}$
- $SE_{Placebo} = \sqrt{(2\mu_{Placebo})^2\sigma_{Placebo}}$
- $SE_{Difference} = \sqrt{(2\mu_{Placebo})^2\sigma_{Placebo} + (-2\mu_{Active})^2\sigma_{Active} + 2(2\mu_{Placebo})(-2\mu_{Active})cov}$

Table 14 SAS code for primary efficacy analysis

Data set	SAS code
All subjects in FAS with at least one diary record in the primary efficacy assessment	proc mixed method=reml plots=studentpanel(marginal conditional);<br>class treatment cohort creg;<br>model $TCRS_{sqrt}$= treatment cohort base_$TCRS_{sqrt}$ / ddfm = KenwardRogers residual;<br>random creg (cohort) ;<br>repeated/group=treatment;

period	lsmeans treatment / cl diff om;<br>run;

$TCRS_{sqrt}$ is the square root of the average TCRS, base_$TCRS_{sqrt}$ is the square root of the average TCRS at baseline, and creg is country/region, and the option om is used for the analysed population at baseline.

9.2.2 GLMM analyses

The daily binary responses will be analysed using a GLMM with a logit link function. The model includes the daily binary response as the response variable, treatment group and cohort as fixed factors, the relevant baseline value (from the model specification) as a covariate, and country/region within cohort, and subject as random effects.

The odds ratio for the response for active treatment relative to placebo will be presented together with the coherent p-values and 95% confidence limits.

Convergence issues:

If the model does not converge, country/region will be pooled into larger regions. If the model still does not converge, the random effect of region within cohort will be removed from the model.

Table 15 SAS code for additional efficacy analysis (binary endpoint)

Data set	SAS code
All subjects in FAS with at least one eDiary record in the primary efficacy assessment period	proc glimmix;<br>class treatment cohort creg subject;<br>model binary_variable (event='1') = treatment cohort baseline/ ddfm=kr solution dist=binary link=logit oddsratios;<br>random creg (cohort) subject;<br>lsmeans treatment /diff=control('Placebo') oddsratio cl ilink om;<br>run;

creg is country/region, and baseline is the relevant baseline value from the model specification.

9.3 Subjects excluded from the sensitivity analysis

The subject ID for the 10 subjects who violated at least one inclusion criteria are:

[redacted]

The subject ID for the siblings participating in the same cohort are:

[redacted]

[redacted]

[redacted]

The subject ID for the subjects in [redacted] temporarily without IMP are:

[redacted]

10 References

Carpenter, James R; Goldstein, Harvey; Jon, Rasbash (2003): A Novel Bootstrap Procedure for Assessing the Relationship between Class Size and Achievement 52 (4), pp. 431–443. DOI: 10.1111/1467-9876.00415.

Fieller, E. C. (1954): Some problems in interval estimation. In *J. R. Stat. Soc. Series B (Methodological)* 16 (2), pp. 175–185.

ICH (1998): ICH Harmonised Tripartite Guideline Topic E9: Statistical Principles for Clinical Trials.

ICH (2019): ICH E9 (R1) addendum on estimands and sensitivity analysis in clinical trials to the guideline on statistical principles for clinical trials (step 4).

Juniper, E. F.; Howland, W. C.; Roberts, N. B.; Thompson, A. K.; King, D. R. (1998): Measuring quality of life in children with rhinoconjunctivitis. In *J. Allergy Clin. Immunol.* 101 (2 Pt 1), pp. 163–170. DOI: 10.1016/s0091-6749(98)70380-x.

Kenward, M. G.; Roger, J. H. (1997): Small sample inference for fixed effects from restricted maximum likelihood. In *Biometrics* 53 (3), pp. 983–997.

Passalacqua, Giovanni; Baena-Cagnani, Carlos E.; Bousquet, J.; Canonica, Giorgio W.; Casale, T. B.; Cox, Linda S. et al. (2013): Grading local side effects of sublingual immunotherapy for respiratory allergy: speaking the same language. In *J. Allergy Clin. Immunol.* 132 (1), pp. 93–98. DOI: 10.1016/j.jaci.2013.03.039.

Pfaar, O.; Demoly, P.; van Gerth Wijk, R.; Bonini, S.; Bousquet, J.; Canonica, Giorgio W. et al. (2014): Recommendations for the standardization of clinical outcomes used in allergen immunotherapy trials for allergic rhinoconjunctivitis: an EAACI Position Paper. In *Allergy Eur. J. Allergy Clin. Immunol.* 69 (7), pp. 854–867. DOI: 10.1111/all.12383.

Approval Task	[redacted] 24-May-2023 07:42:34 GMT+0000

Approval Task	[redacted] 24-May-2023 07:45:14 GMT+0000

Approval Task	[redacted] 24-May-2023 07:46:05 GMT+0000

Approval Task	[redacted] 24-May-2023 08:14:03 GMT+0000

Approval Task	[redacted] 24-May-2023 12:20:52 GMT+0000

Table S6: Baseline demographics and clinical characteristics (FAS)

	Placebo (N=731)	SQ HDM SLIT-tablet (N=727)
**Demographics**		
Race		
Asian	3 (0·4%)	1 (0·1%)
Black or African American	4 (0·5%)	1 (0 1%)
White	714 (97·7%)	722 (99·3%)
American Indian or Alaska Native	··	1 (0·1%)
Multiple	3 (0·4%)	1 (0·1%)
Other	7 (1·0%)	1 (0·1%)
Ethnicity		
Hispanic or Latino	19 (2·6%)	26 (3·6%)
Not Hispanic or Latino	697 (95·3%)	688 (94·6%)
Not reported	15 (2·1%)	13 (1·8%)
**Clinical characteristics**		
Mean SPT wheal size, diameter in mm (SD)		
D. pteronyssinus	7·1 (2·5)*	7·1 (2·5)†
D. farinae	6·5 (2·3)*	6·5 (2·6)‡
Mean IgE levels at baseline, kU/L (SD)		
IgE against D. pteronyssinus	50·7 (36·0)	49·4 (35·1)
IgE against D. farinae	53·1 (34·7)	53·2 (34·4)
Highest of D. pteronyssinus and D. farinae	59·2 (35·3)	58·8 (34·5)
Median pulmonary function at baseline, % predicted (min–max)		
FVC	95·3 (61–184)	96·2 (52–163)

Data presented are n (%), unless otherwise stated. *n=722. †n=714. ‡n=715. *D. farinae*=*Dermatophagoides farinae*. *D. pteronyssinus*=*Dermatophagoides pteronyssinus*. FAS=full analysis set. FVC=forced vital capacity. HDM=house dust mite. IgE=immunoglobulin type E. N=number of subjects in the FAS. SD=standard deviation. SLIT=sublingual immunotherapy. SPT=skin prick test.

Table S7: Sensitivity and estimand analyses of the primary and key secondary outcomes during the primary efficacy assessment period (FAS)

Analysis frame	Analysis name*	Absolute treatment difference (95% CI)	Relative treatment difference (95% CI)	p-value
**Average daily TCRS**				
Observed case	Primary analysis	1·0 (0·5, 1·4)	22·0% (12·0, 31·1)	<0·0001
	Sensitivity analysis (potential data issues)	1·0 (0·5, 1·5)	22·6% (12·7, 31·7)	<0·0001
	Sensitivity analysis (modified DMS scoring)	1·0 (0·5, 1·5)	21·9% (11·7, 31·1)	<0·0001
Trial product estimand	Main analytical approach	1·0 (0·5, 1·5)	22·2% (12·2, 31·2)	<0·0001
	Sensitivity 1	1·0 (0·5, 1·4)	21·9% (11·9, 30·9)	<0·0001
	Sensitivity 2	1·0 (0·5, 1·4)	21·5% (11·5, 30·6)	<0·0001
Treatment policy estimand	Main analytical approach	1·0 (0·5, 1·4)	21·8% (11·8, 30·8)	<0·0001
	Sensitivity	0·9 (0·5, 1·4)	21·1% (11·0, 30·2)	0·0001
**Average rhinitis DSS**				
Observed case	Primary analysis	0·4 (0·2, 0·6)	22·0% (12·8, 30·8)	<0·0001
Trial product estimand	Main analytical approach	0·4 (0·2, 0·6)	22·5% (13·2, 31·1)	<0·0001
Treatment policy estimand	Main analytical approach	0·4 (0·2, 0·6)	22·1% (12·7, 30·6)	<0·0001
**Average rhinitis DMS**				
Observed case	Primary analysis	0·5 (0·2, 0·8)	25·3% (10·5, 38·3)	0·0016
	Sensitivity (modified DMS scoring)	0·5 (0·2, 0·8)	25·3% (10·2, 38·4)	0·0019
Trial product estimand	Main analytical approach	0·5 (0·2, 0·8)	25·4% (10·7, 38·2)	0·0015
Treatment policy estimand	Main analytical approach	0·5 (0·2, 0·8)	24·9% (10·1, 37·7)	0·0018
**Average rhinoconjunctivitis TCS**				
Observed case	Primary analysis	1·1 (0·6, 1·7)	22·2% (12·0, 31·5)	<0·0001
	Sensitivity (modified DMS scoring)	1·2 (0·6, 1·7)	22·3% (12·0, 31·6)	<0·0001
Trial product estimand	Main analytical approach	1·2 (0·6, 1·7)	22·4% (12·3, 31·6)	<0·0001
Treatment policy estimand	Main analytical approach	1·1 (0·6, 1·7)	22·0% (11·9, 31·2)	<0·0001

*Please refer to the statistical analysis plan for a detailed description of each analysis. CI=confidence interval. DMS=daily medication score. DSS=daily symptom score. FAS=full analysis set. TCRS=total combined rhinitis score. TCS=total combined score. Number of subjects with imputed data in the estimand analyses: for trial product estimand, placebo n=26 and SQ HDM SLIT-tablet n=37; for treatment policy estimand, placebo n=25 and SQ HDM SLIT-tablet, n=34.

Figure S2: Primary efficacy outcome (TCRS) for the SQ HDM SLIT-tablet versus placebo, by asthma and sensitisation status at baseline (FAS, observed cases)

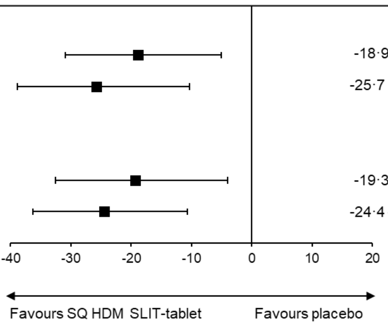

	Relative difference* (%) [95% CI]	Placebo (n)	SQ HDM SLIT-tablet (n)	Adjusted mean (SE) Placebo	Adjusted mean (SE) SQ HDM SLIT-tablet	Absolute difference [95% CI]	p-value
**Asthma status at baseline:**							
Asthma no	-18·9 [-30·9, -5·1]	426	442	4·1 (0·4)	3·3 (0·3)	0·8 [0·2, 1·4]	0·009
Asthma yes	-25·7 [-38·8, -10·3]	280	251	4·9 (0·4)	3·6 (0·4)	1·3 [0·5, 2·1]	0·002
**Sensitisation status at baseline:**							
Monosensitised	-19·3 [-32·5, -4·0]	346	327	4·3 (0·4)	3·5 (0·3)	0·8 [0·2, 1·5]	0·02
Polysensitised	-24·4 -[36·3, -10·7]	360	366	4·5 (0·4)	3·4 (0·3)	1·1 [0·4, 1·8]	0·0010

Sensitisation status at baseline was based on SPT data. Interaction effect: p=0·4 between asthma status and treatment measured as TCRS; p=0·6 between sensitisation status and treatment measured as TCRS. *Calculated as SQ HDM SLIT-tablet minus placebo divided by placebo. CI=confidence interval. FAS=full analysis set. HDM=house dust mite. n=number of subjects in the FAS with data contributing to the analysis. SE=standard error. SLIT=sublingual immunotherapy. SPT=skin prick test. TCRS=total combined rhinitis score.

Table S8: Key efficacy outcomes at Week 8 and Week 16 (FAS, observed cases)

Range	Adjusted mean (SE)		Absolute difference (95% CI)	Relative difference (95% CI)	p-value
	Placebo	SQ HDM SLIT-tablet			
**Week 8**					
TCRS	6·8 (0·4)	6·0 (0·4)	0·8 (0·2, 1·5)	12·1% (3·0, 20·4)	0·0105
Rhinitis DSS	2·9 (0·2)	2·6 (0·2)	0·3 (0·1, 0·6)	11·1% (2·2, 19·3)	0·0162
Rhinitis DMS	3·3 (0·3)	2·8 (0·2)	0·5 (0·0, 0·9)	14·7% (1·2, 26·5)	0·0339
Rhinoconjunctivitis TCS	8·0 (0·5)	7·1 (0·4)	1·0 (0·2, 1·7)	12·4% (3·2, 20·7)	0·0091
**Week 16**					
TCRS	5·2 (0·4)	4·5 (0·4)	0·7 (0·1, 1·3)	13·5% (2·5, 23·4)	0·0172
Rhinitis DSS	2·3 (0·2)	2·0 (0·2)	0·3 (0·0, 0·5)	12·4% (2·0, 21·8)	0·0209
Rhinitis DMS	2·2 (0·2)	1·8 (0·2)	0·4 (0·0, 0·7)	17·0% (0·3, 31·3)	0·0463
Rhinoconjunctivitis TCS	6·1 (0·4)	5·2 (0·4)	0·8 (0·2, 1·5)	13·7% (2·6, 23·7)	0·0167

Week 8: placebo, n=705/731 (704/731 for TCS); SQ HDM SLIT-tablet, n=695/727; Week 16: placebo, n=709/731; SQ HDM SLIT-tablet, n=690/727. Scoring: rhinitis DSS, 0–12 points; rhinitis DMS, maximum 12 points; rhinoconjunctivitis TCS, 0–38 points. CI=confidence interval. DMS=daily medication score. DSS=daily symptom score. FAS=full analysis set. HDM=house dust mite. n=number of subjects in the FAS with data contributing to the analysis. SE=standard error. SLIT=sublingual immunotherapy. TCRS=total combined rhinitis score. TCS=total combined score.

Table S9: Pre-specified asthma-related outcomes during the primary efficacy assessment period (FAS, observed cases)

	Placebo (n=280)	SQ HDM SLIT-tablet (n=251)	Absolute difference (95% CI)	Relative difference (95% CI)	Odds ratio (95% CI)	p-value
**Adjusted mean (SE)**						
Asthma DSS	0·4 (0·0)	0·3 (0·0)	0·1 (0·0, 0·2)	27·8% (3·9, 46·5)	··	0·026
Number of as-needed SABA puffs per week*	1·5 (0·2)	1·0 (0·2)	0·4 (-0·1, 1·0)	29·6% (-10·8, 57·4)	··	0·13
**Proportion**						
SABA-free days	0·986	0·992	··	··	1·8 (1·0, 3·3)	0·053
Nocturnal awakenings requiring SABA use	0·008	0·005	··	··	0·6 (0·4, 1·0)	0·028

*The average weekly number of puffs of as-needed SABA use was calculated as seven times the average of the daily number of puffs of as-needed SABA use. CI=confidence interval. DSS=daily symptom score. FAS=full analysis set. HDM=house dust mite. n=number of subjects in the FAS with data contributing to the analysis. SABA=short-acting beta agonist. SE=standard error. SLIT=sublingual immunotherapy.

Figure S3: Mean change from baseline to the end-of-trial visit in immunological parameters (FAS, Canada and Poland)

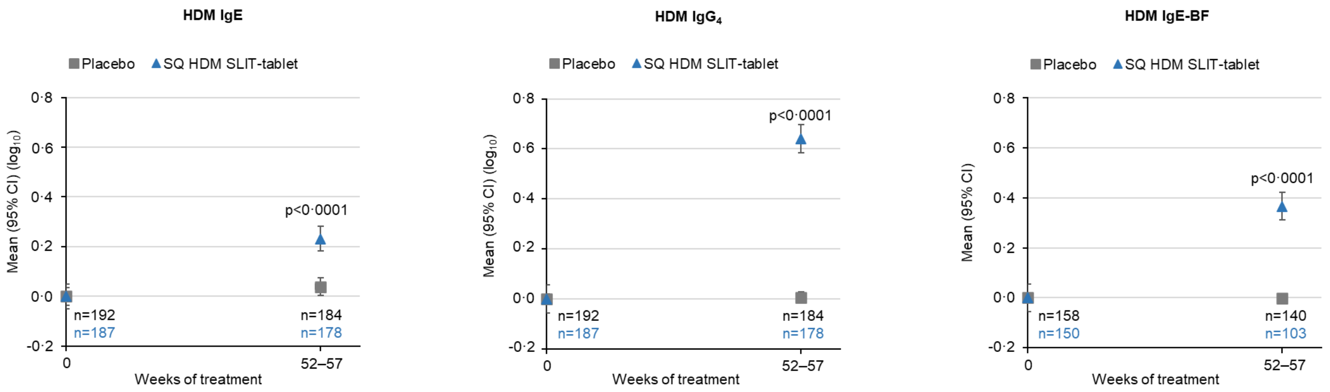

Immunological parameters were evaluated from children recruited in Canada and Poland only. Data are for *D. pteronyssinus*; similar results were observed for *D. farinae* for HDM IgE and HDM IgG_4 (HDM IgE-BF not evaluated for *D. farinae*). P-values reflect the between-group differences. *D. farinae*=*Dermatophagoides farinae*. *D. pteronyssinus*=*Dermatophagoides pteronyssinus*. CI=confidence interval. FAS=full analysis set. HDM=house dust mite. IgE=immunoglobulin type E. IgE-BF=immunoglobulin type E-blocking factor. IgG_4=immunoglobulin type G, class 4. n=number of subjects contributing to the mean values. SLIT=sublingual immunotherapy.

Table S10: Summary of the most frequent TRAEs reported in at least 2% of subjects in the SQ HDM SLIT-tablet group (safety analysis set)

	**Placebo (N=731)**	**SQ HDM SLIT-tablet (N=727)**
**All TRAEs**	391 (53·5%)	548 (75·4%)
Oral pruritus	175 (23·9%)	414 (56·9%)
Throat irritation	227 (31·1%)	397 (54·6%)
Ear pruritus	123 (16·8%)	238 (32·7%)
Abdominal pain upper	118 (16·1%)	207 (28·5%)
Lip swelling	37 (5·1%)	148 (20·4%)
Glossodynia	38 (5·2%)	140 (19·3%)
Nausea	66 (9·0%)	115 (15·8%)
Taste disorder	105 (14·4%)	115 (15·8%)
Swollen tongue	19 (2·6%)	99 (13·6%)
Mouth swelling	24 (3·3%)	97 (13·3%)
Mouth ulceration	46 (6·3%)	71 (9·8%)
Pharyngeal swelling	21 (2·9%)	67 (9·2%)
Diarrhoea	44 (6·0%)	59 (8·1%)
Tongue ulceration	25 (3·4%)	47 (6·5%)
Vomiting	17 (2·3%)	34 (4·7%)

Data presented are n (%). TRAEs are TEAEs reported as ‘possibly’ related to trial medication by the investigator. HDM=house dust mite. N=number of subjects in the safety analysis set. SLIT=sublingual immunotherapy. TEAE=treatment-emergent adverse event. TRAE=treatment-related adverse event.

Table S11: Summary of TEAEs (safety analysis set)

	Placebo (N=731)		SQ HDM SLIT-tablet (N=727)	
	**Number of subjects**	**Number of events**	**Number of subjects**	**Number of events**
All events	585 (80·0%)	3423 (100·0%)	632 (86·9%)	6489 (100·0%)
**Severity**				
Mild	557 (76·2%)	3016 (88·1%)	624 (85·8%)	5886 (90·7%)
Moderate	205 (28·0%)	401 (11·7%)	227 (31·2%)	580 (8·9%)
Severe	6 (0·8%)[*]	6 (0·18%)	15 (2·1%)[*]	23 (0·35%)
Serious	6 (0·8%)[†]	6 (0·18%)	16 (2·2%)[†]	18 (0.28%)
**Outcome**[‡]				
Recovered/resolved	584 (79·9%)[⁋]	3407 (99·5%)	631 (86·8%)[⁋]	6451 (99·4%)
Recovered/resolved with sequelae	0 (0·0%)[⁋]	0 (0·0%)	2 (0·3%)[⁋]	2 (<0·1%)
Not recovered/not resolved	13 (1·8%)[⁋]	14 (0·4%)	20 (2·8%)[⁋]	25 (0·4%)
Unknown	2 (0·3%)[⁋]	2 (<0·1%)	10 (1·4%)[⁋]	11 (0·2%)
**Changes to trial medication due to TEAE**[†]				
None	527 (72·1%)[⁋]	2957 (86·4%)	599 (82·4%)[⁋]	5892 (90·8%)
Trial medication interrupted	237 (32·4%)[⁋]	458 (13·4%)	234 (32·2%)[⁋]	558 (8·6%)
Trial medication withdrawn	7 (1·0%)[⁋]	8 (0·2%)	17 (2·3%)[⁋]	35 (0·5%)
Not applicable	0 (0·0%)[⁋]	0 (0·0%)	4 (0·6%)[⁋]	4 (<0·1%)

HDM=house dust mite. N=number of subjects in the safety analysis set. SLIT=sublingual immunotherapy. TEAE=treatment-emergent adverse event. [*]Placebo: abdominal pain, appendicitis, pneumonia, testicular torsion (all one event in one subject), rhinitis allergic (two events in two subjects); SQ HDM SLIT-tablet: abdominal pain upper, laryngitis, tonsilitis, ankle fracture, fracture, attention deficit hyperactivity disorder, hallucinations mixed, immune system disorder, migraine, allergic rhinitis (all one event in one subject), glossodynia (two events in one subject), influenza (two events in two subjects), ear pruritus, oral pruritus (three events in one subject), lip swelling (three events in two subjects). [†]Placebo: appendicitis, nasopharyngitis, carbon monoxide poisoning, testicular torsion (all one event in one subject), pneumonia (two events in two subjects); SQ HDM SLIT-tablet: COVID-19, campylobacter gastroenteritis, influenza, laryngitis, pneumonia, pseudomonas bronchitis, tonsilitis, contusion, fracture, attention deficit hyperactivity disorder, hallucinations mixed, nausea, vomiting, non-cardiac chest pain, immune system disorder, angioedema (all one event in one subject), gastroenteritis norovirus (two events in two subjects). [‡]Some subjects reported several events, each with different outcomes. [⁋]Percentages calculated from the total number of subjects (placebo, N=731; SQ HDM SLIT-tablet, N=727).

Table S12: Onset and duration of the three most frequent TRAEs occurring in at least 2% of subjects in the SQ HDM SLIT-tablet group (safety analysis set)

	Placebo (N=731)			SQ HDM SLIT-tablet (N=727)		
	Time to onset on first day (mins)*	Time to onset (days)†	Duration (days)‡	Time to onset on first day (mins)*	Time to onset (days)†	Duration (days)‡
**Oral pruritus**	8·0 (0–480)	3·0 (1–72)	1·0 (1–154)	3·0 (0–270)	1·5 (1–141)	2·0 (1–393)
**Throat irritation**	5·0 (0–257)	3·0 (1–211)	1·0 (1–170)	5·0 (0–575)	2·0 (1–28)	2·0 (1–383)
**Ear pruritus**	15·0 (1–40)	4·0 (1–28)	1·0 (1–67)	6·0 (1–110)	4·0 (1–27)	1·0 (1–167)

Data presented are median (range). TRAEs are TEAEs reported as 'possibly' related to trial medication by the investigator. Number of subjects per event: ear pruritus – placebo n=123, SQ HDM SLIT-tablet n=238; throat irritation – placebo n=227, SQ HDM SLIT-tablet n=397; oral pruritus – placebo n=174, SQ HDM SLIT-tablet n=414. Number of events: ear pruritus – placebo n=284, SQ HDM SLIT-tablet n=533; throat irritation – placebo n=488, SQ HDM SLIT-tablet n=1059; oral pruritus – placebo n=409, SQ HDM SLIT-tablet n=1135. *For events that started on the first day of trial medication. †For each subject, only the first event within a category is included. ‡Calculated as the number of days elapsed from the start to the end date of the AE. AE=adverse event. HDM=house dust mite. N=number of subjects in the safety analysis set. SLIT=sublingual immunotherapy. TEAE=treatment-emergent adverse event. TRAE=treatment-related adverse event.

Table S13: Summary of the most frequent TEAEs reported in at least 2% of subjects in the SQ HDM SLIT-tablet group (safety analysis set)

	**Placebo (N=731)**	**SQ HDM SLIT-tablet (N=727)**
**All TEAEs**	585 (80·0%)	632 (86·9%)
Oral pruritus	185 (25·3%)	419 (57·6%)
Throat irritation	236 (32·3%)	401 (55·2%)
Abdominal pain upper	164 (22·4%)	243 (33·4%)
Ear pruritus	135 (18·5%)	242 (33·3%)
Nasopharyngitis	164 (22·4%)	185 (25·4%)
Lip swelling	38 (5·2%)	151 (20·8%)
Glossodynia	41 (5·6%)	142 (19·5%)
Nausea	81 (11·1%)	136 (18·7%)
Taste disorder	116 (15·9%)	123 (16·9%)
Mouth swelling	27 (3·7%)	99 (13·6%)
Swollen tongue	20 (2·7%)	99 (13·6%)
Diarrhoea	74 (10·1%)	95 (13·1%)
Mouth ulceration	53 (7·3%)	93 (12·8%)
Pharyngeal swelling	22 (3·0%)	68 (9·4%)
Tongue ulceration	27 (3·7%)	50 (6·9%)
Vomiting	33 (4·5%)	49 (6·7%)
Tooth loss	35 (4·8%)	40 (5·5%)
COVID-19	38 (5·2%)	39 (5·4%)
Pharyngitis	37 (5·1%)	38 (5·2%)
Bronchitis	45 (6·2%)	36 (5·0%)
Respiratory tract infection	24 (3·3%)	26 (3·6%)
Influenza	24 (3·3%)	24 (3·3%)
Upper respiratory tract infection	37 (5·1%)	22 (3·0%)
Tonsilitis	17 (2·3%)	16 (2·2%)

Data presented are n (%). COVID-19=COronaVIrus Disease of 2019. HDM=house dust mite. N=number of subjects in the safety analysis set. SLIT=sublingual immunotherapy. TEAE=treatment-emergent adverse event.

Narratives for subjects reporting anaphylactic/systemic allergic reactions

Anaphylactic/systemic allergic reactions classified as ESIs were reported for three subjects in the SQ HDM SLIT-tablet group and two subjects in the placebo group.

Possibly related to trial medication

Subject 1: an 11-year-old male experienced itchy confluent urticarial rash on the chest and abdomen of mild intensity at Day 184 post-treatment initiation. The subject was treated with oral loratadine (10 mg). The event lasted for 30 minutes and was recurrent for the next 66 days, at which point the subject recovered. There was no change in SQ HDM SLIT-tablet treatment.

Subject 2: an 11-year-old male experienced epigastric pain and malaise of moderate intensity 6 minutes after treatment initiation. The subject was treated with one dose of oral loratadine (10 mg) and recovered the same day. Treatment with SQ HDM SLIT-tablet was discontinued.

Subject 3: a 6-year-old male experienced acute urticaria (rash on trunk) 10 days after the first intake of placebo, was treated with oral desloratadine (2.5 mg daily) and recovered after 6 days. There was no change in placebo treatment.

Unlikely related to trial medication

Anaphylactic/systemic allergic reactions classified as ESIs and considered unlikely related to trial medication were reported for one subject in the SQ HDM SLIT-tablet group and one subject in the placebo group.

Subject 1: a 5-year-old male experienced angioedema 12 hours after the last intake of SQ HDM SLIT-tablet (51 days after initiating treatment). Moderate facial swelling developed, the subject was hospitalised for treatment with dexamethasone (8 mg, intramuscular), budesonide (unknown dose, inhaled), chloropyramine (10 mg, intramuscular), ipratropium bromide (15 drops, inhalation), and cetirizine (5 mg, oral), and he recovered after 1 day. Treatment with SQ HDM SLIT-tablet was temporarily interrupted and the subject’s consent for study participation was withdrawn 17 days after the event.

Subject 2: a 5-year-old male in the placebo group experienced an anaphylactic reaction with swelling to the face and erythema following ingestion of nuts; the subject had a known nut allergy. Treatment was with oral desloratadine (dose unknown) and the subject recovered after 6 days. Treatment with placebo was temporarily interrupted for 4 days.
